# Tracking SARS-CoV-2 introductions in Mozambique using pandemic-scale phylogenies: a retrospective observational study

**DOI:** 10.1016/S2214-109X(23)00169-9

**Published:** 2023-05-16

**Authors:** Francisco José Martínez-Martínez, Arsenia J Massinga, Áuria De Jesus, Rita M Ernesto, Pablo Cano-Jiménez, Álvaro Chiner-Oms, Inmaculada Gómez-Navarro, Marina Guillot-Fernández, Caterina Guinovart, António Sitoe, Delfino Vubil, Rubão Bila, Rufino Gujamo, Sónia Enosse, Santiago Jiménez-Serrano, Manuela Torres-Puente, Iñaki Comas, Inácio Mandomando, Mariana G López, Alfredo Mayor

**Affiliations:** a Tuberculosis Genomics Unit, Instituto de Biomedicina de Valencia, Consejo Superior de Investigaciones Científicas, Valencia, Spain; b Centro de Investigação em Saúde de Manhiça, Maputo, Mozambique; c ISGlobal, Hospital Clínic - Universitat de Barcelona, Barcelona, Spain; d Hospital Distrital da Manhiça, Marracuene, Mozambique; e Instituto Nacional de Saúde, Marracuene, Mozambique; f Centro de Investigación Biomédica en Red en Epidemiología y Salud Pública (CIBERESP), Madrid, Spain; g Department of Physiologic Sciences, Faculty of Medicine, Universidade Eduardo Mondlane, Maputo, Mozambique

## Abstract

**Background:**

From the start of the SARS-CoV-2 outbreak, global sequencing efforts have generated an unprecedented amount of genomic data. Nonetheless, unequal sampling between high-income and low-income countries hinders the implementation of genomic surveillance systems at the global and local level. Filling the knowledge gaps of genomic information and understanding pandemic dynamics in low-income countries is essential for public health decision making and to prepare for future pandemics. In this context, we aimed to discover the timing and origin of SARS-CoV-2 variant introductions in Mozambique, taking advantage of pandemic-scale phylogenies.

**Methods:**

We did a retrospective, observational study in southern Mozambique. Patients from Manhiça presenting with respiratory symptoms were recruited, and those enrolled in clinical trials were excluded. Data were included from three sources: (1) a prospective hospital-based surveillance study (MozCOVID), recruiting patients living in Manhiça, attending the Manhiça district hospital, and fulfilling the criteria of suspected COVID-19 case according to WHO; (2) symptomatic and asymptomatic individuals with SARS-CoV-2 infection recruited by the National Surveillance system; and (3) sequences from SARS-CoV-2-infected Mozambican cases deposited on the Global Initiative on Sharing Avian Influenza Data database. Positive samples amenable for sequencing were analysed. We used Ultrafast Sample placement on Existing tRees to understand the dynamics of beta and delta waves, using available genomic data. This tool can reconstruct a phylogeny with millions of sequences by efficient sample placement in a tree. We reconstructed a phylogeny (~7·6 million sequences) adding new and publicly available beta and delta sequences.

**Findings:**

A total of 5793 patients were recruited between Nov 1, 2020, and Aug 31, 2021. During this time, 133 328 COVID-19 cases were reported in Mozambique. 280 good quality new SARS-CoV-2 sequences were obtained after the inclusion criteria were applied and an additional 652 beta (B.1.351) and delta (B.1.617.2) public sequences were included from Mozambique. We evaluated 373 beta and 559 delta sequences. We identified 187 beta introductions (including 295 sequences), divided in 42 transmission groups and 145 unique introductions, mostly from South Africa, between August, 2020 and July, 2021. For delta, we identified 220 introductions (including 494 sequences), with 49 transmission groups and 171 unique introductions, mostly from the UK, India, and South Africa, between April and November, 2021.

**Interpretation:**

The timing and origin of introductions suggests that movement restrictions effectively avoided introductions from non-African countries, but not from surrounding countries. Our results raise questions about the imbalance between the consequences of restrictions and health benefits. This new understanding of pandemic dynamics in Mozambique can be used to inform public health interventions to control the spread of new variants.

**Funding:**

European and Developing Countries Clinical Trials, European Research Council, Bill & Melinda Gates Foundation, and Agència de Gestió d’Ajuts Universitaris i de Recerca.

## Introduction

Since the publication of the first SARS-CoV-2 genome (January, 2020), several studies have tracked the evolution of the virus worldwide.^1^ Next-generation sequencing of SARS-CoV-2 allows a better understanding of transmission dynamics and potential mechanisms of immune evasion, as well as associations with pathogenicity and vaccine efficacy.^2^, ^3^, ^4^, ^5^ These continued surveillance efforts have identified the emergence of several variants of global concern (VOCs), including alpha (B.1.1.7), first identified in the UK; beta (B.1.351), first identified in South Africa; gamma (P.1), first identified in Brazil; delta (B.1.617·2), first identified in India; and omicron (B.1.1.529), also first identified in South Africa, among others. These VOCs were associated with rapid expansion both in the regions they were first reported in and other nations, leading to an increase of COVID-19 cases, for instance, as seen in Europe in August**,** 2020, where introductions from other countries were associated with the second wave.^6^

**Research in context**

**Evidence before this study**

Mozambique constitutes an entry point from South Africa for SARS-CoV-2 variants which can have major repercussions for the entire African continent. We searched PubMed for publications, without language restrictions, published from March 1, 2020, until Jan 13, 2022, using the search terms (“SARS-CoV-2” OR “COVID-19”) AND “genomic surveillance” AND “Africa”. The search showed the scarce data regarding the epidemiology and genetic characteristics of SARS-CoV-2 in Africa. Of the 46 results obtained in the search, eight studies implemented some sort of genomic epidemiology analysis in Africa, but there are no specific studies from Mozambique. The most relevant study found in the search used a dataset of 8746 genomes from 33 African countries and two overseas territories, and described the detection of variants in South Africa and their spread into neighbouring countries.

**Added value of this study**

For the first time using data from Africa, we used a maximum parsimony approach (termed Ultrafast Sample Placement on Existing Trees) for the rapid sample placement in a phylogeny based on more than 7·6 million sequences publicly available until Feb 9, 2022, and generated here, allowing us to minimise sampling biases. This first genomic surveillance study of SARS-CoV-2 in Mozambique identified B.1.351 (beta) and B.1.617·2 (delta) as the major lineages during the COVID-19 waves in January–March and June–September, 2021, respectively. Almost 190 independent introductions of the beta variant were identified throughout the January–March 2021 wave, with about 80% of them occurring in 7 months and mostly coming from South Africa. By contrast, the 220 independent introductions of delta identified throughout the June–September, 2021 wave, with about 80% of them occurring within 4 months, mainly originated from the UK, India, and South Africa. The fast replacement of beta by delta in Mozambique might be due to the known higher transmissibility of delta and an increasing contribution of community transmission.

**Implications of all the available evidence**

SARS-CoV-2 was predominantly transmitted in Mozambique through regional migrations during the beta wave and international migrations during the delta wave. Restrictions in movement between Mozambique and other countries as well as visa issuance suspensions probably helped avoid the introduction of variants from non-African countries, but not from surrounding countries, potentially due to porous borders between Mozambique and neighbouring countries. Thus, the measures implemented had less benefits in terms of virus containment than expected.

Although many countries have developed extensive genetic surveillance and reporting systems, several regions across the globe remain crucially undersampled.^7^ Most African countries, with the exception of South Africa,^8^ have reported only a handful of sequences.^9^, ^10^, ^11^, ^12^ To date, no studies have reported on SARS-CoV-2 genomic lineages in Mozambique and how, when, and from where they were initially introduced.^13^ Importantly, identification and quantification of import and export events is highly conditioned by the dataset used to compare. Phylogenetic tools are now available to incorporate the entire deposited Global Initiative on Sharing Avian Influenza Data (GISAID) database,^14^ which includes millions of SARS-CoV-2 sequences to evaluate and understand local epidemics. However, very few studies have taken advantage of these developments and still use biased datasets (eg, regional or country-restricted data) because of the computational need of subsampling, or pre-conceptions on which countries’ datasets are more relevant.

The first COVID-19 case was reported in Mozambique on March 22, 2020 in a 75-year-old patient who returned from the UK; since then, the country has experienced four waves.^13^ Concern about the potential spread of SARS-CoV-2 was elevated in late March, 2020, when more than 14 000 Mozambicans returned from South Africa over the Ressano Garcia border, as South Africa declared a lockdown. By June 2, 2021, Mozambique had reported a total of 70 850 confirmed cases and 836 deaths, with community-driven transmission. A continuous effort to sequence a representative number of those cases emerging in the country is required to detect new and emerging variants that might have increased pathogenicity, as well as to identify potential routes of transmission. In this study, we performed next-generation sequencing to understand the SARS-CoV-2 genomes that circulated in Mozambique during the peak of transmission between November, 2020, and August, 2021.^15^ We quantified transmission groups and estimated the number, origin, and time span of introductions. We reconstructed phylogenies with more than 7·6 million sequences using Ultrafast Sample placement on Existing tRees (UshER) to minimise sampling bias,^14^ and evaluate Mozambican genomic data in the context of all the worldwide available SARS-CoV-2 diversity.

## Methods

### Study design and data sources

This genomic epidemiology study covered Mozambique. Data were included from three different sources: (1) a prospective hospital-based surveillance study (MozCOVID; appendix pp 1–35), conducted by Centro de Investigação em Saúde de Manhiça (CISM), recruiting patients living in Manhiça district, southern Mozambique, attending the Manhiça district hospital, and fulfilling the criteria of suspected COVID-19 case according to WHO; (2) symptomatic and asymptomatic individuals living in Manhiça recruited by the National Surveillance system; and (3) sequences from SARS-CoV-2-infected Mozambican cases deposited on the GISAID database public repository until Jan 11, 2022. Health personnel completed a questionnaire for each participant of the MozCOVID study (appendix pp 36–39). Study design is expanded on in the appendix (p 71). All participants provided written informed consent. The study was approved by the CISM institutional ethics committee and the Mozambican Ministry of Health National Bioethics Committee (reference number, 80/CNBS/2020). The study protocol has previously been published.

### Procedures

Nasopharyngeal swabs were collected from all patients to detect SARS-CoV-2 by real-time polymerase chain reaction (qPCR). Only those with a cycle threshold of less than 27 were selected for sequencing. Whole viral genome was amplified following ARTIC nCoV-2019 sequencing protocol (version 3) and libraries were prepared for sequencing on the Illumina MiSeq platform (appendix p 71).

Sequence data were analysed following the SeqCOVID-Spain consortium pipeline^16^ (appendix pp 71–72). Briefly, the raw reads were demultiplexed using bcl2fastq (version 2.20, Illumina). The reads were used to generate a consensus sequence. A confident call was defined as having at least a 20 times depth of coverage. Lineages were assigned with the Pangolin tool (version 3.1.1.6). All sequences were aligned against the reference Wuhan Hu-1 (GenBank MN908947.3). Sequences are freely available on GISAID.

### Data and statistical analysis

We used UShER (v.0.5.2) to perform the phylogenetic placement of sequences onto an existing tree (eTree) using a maximum parsimony score.^14^ The eTree included 7 657 161 SARS-CoV-2 worldwide sequences and was downloaded from UCSC FTP server repository on Feb 9, 2022. We performed the phylogenetic placement of the new sequences, along with global beta and delta sequences from GISAID (also downloaded on Feb 9, 2022) that were absent in the eTree. Consensus sequences were transformed into VCF files (appendix p 72). The phylogeny was converted into a format suitable for visualisation on Taxonium (v1) with MatUtils (v 0.5.2).^17^ To make phylogenies manageable, beta and delta clades were pruned from the complete phylogeny.

Beta and delta introductions were estimated from the subtrees obtained. First, transmission groups and unique sequences were defined, and then the potential origin of the introductions was inferred from the closest non-Mozambique sequence considering a diagnosis date happening within 3 months (appendix pp 72–73).^16^

We evaluated the accuracy of the UShER approach in identifying transmission groups and unique sequences, by comparing the beta subtree against a reconstructed phylogeny using IQ-TREE (v.1.6.10). Topology consistency was evaluated by performing the Shimodaira-Hasegawa test (p<0·05; appendix p 73). Graphics were created with the ggplot2 package from R (v.41.2). Scripts were coded in R and python (v 3). Inference of introductions was compared against those from the phylogeographical tool RASP (v 4.3; appendix p 73).

### Role of the funding source

The funders of the study had no role in study design, data collection, data analysis, data interpretation, or writing of the report.

## Results

A total of 5793 patients were recruited between Nov 1, 2020, and Aug 31, 2021. Demographic data, including sex (male or female; collected via survey), age, symptoms, and comorbidities are available in the appendix (pp 40–42).

**Figure 1:**
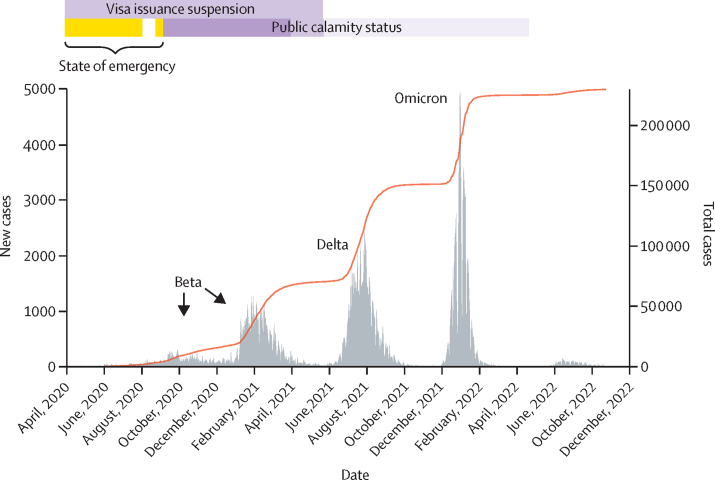
SARS-CoV-2 waves in Mozambique and cumulative total cases Public calamity status began on Sept 7, 2020, replacing the State of emergency. Movement restrictions included: no travel and limited internal movement; during the state of emergency, travels were allowed with mandatory quarantine in Mozambique. Measures were gradually alleviated by April and by the end of May, 2021, when internal movement and restricted social events were permitted.

Mozambique has experienced four SARS-CoV-2 waves (figure 1). The number of cases during the waves increased throughout the pandemic, reaching a maximum in both the third and fourth waves with 70 000 cases. On Sept 9, 2022, Mozambique had reported more than 230 145 infections and 2200 deaths attributed to SARS-CoV-2. Modelling by WHO's Regional Office for Africa suggested that due to under-reporting, the true cumulative number of infections by the end of 2021 was about 14 million (1·2% reported) and the number of deaths was 6222.^18^

**Figure 2:**
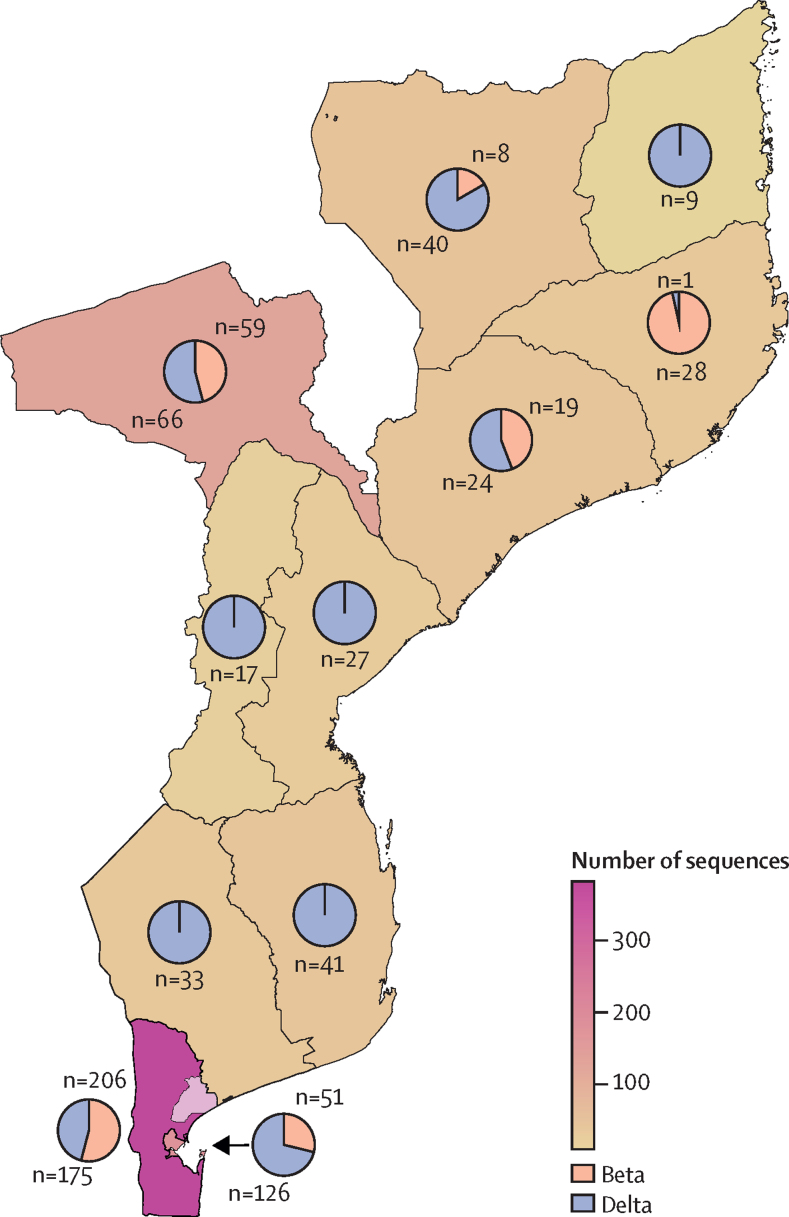
Number of sequenced beta and delta samples per region in Mozambique The map includes 280 samples from this study all taken in Manhiça district, represented with a lighter colour in the Maputo region, in the south of the country, and the Mozambique samples from Global Initiative on Sharing Avian Influenza Data.

Between Nov 1, 2020, and Aug 31, 2021, 133 328 SARS-CoV-2 infections were reported in Mozambique. We obtained 280 good quality new SARS-CoV-2 sequences after the inclusion criteria were applied and we included an additional 652 beta and delta public sequences from Mozambique (figure 2).

**Figure 3:**
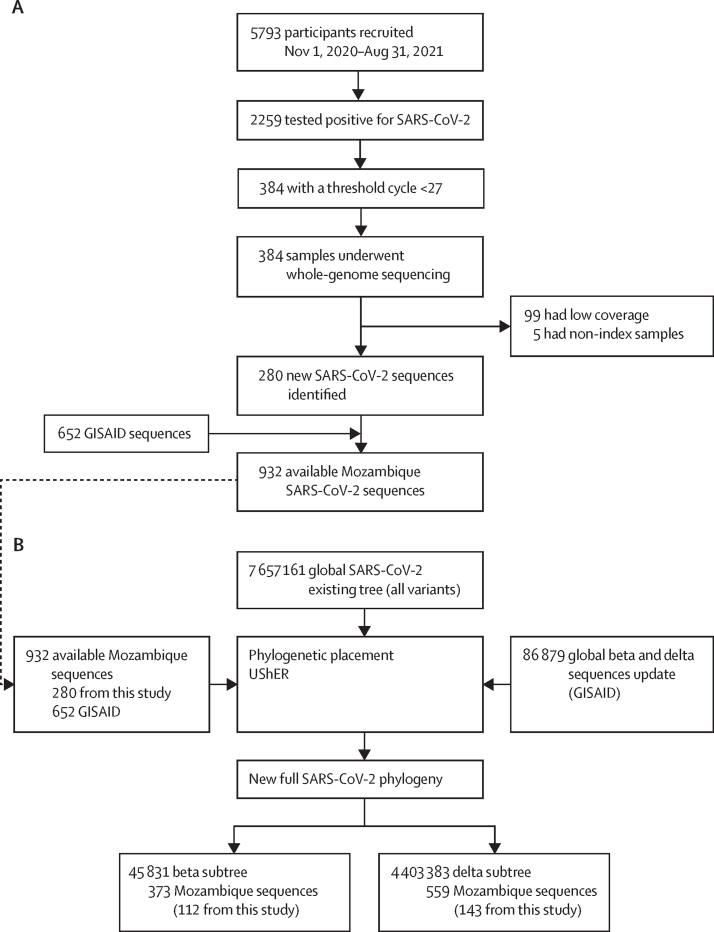
Sample inclusion and sequencing criteria (A) and Mozambique and GISAID enrichment of the eTree and subtree pruning results (B) GISAID=Global Initiative on Sharing Avian Influenza Data. UShER=Ultrafast Sample placement on Existing tRees. Low coverage samples are samples with low sequencing quality. Non-index samples are sequences from a second sample from the same patient.

280 new SARS-CoV-2 sequences were eventually available for sequencing and deposited on GISAID (figure 3A; appendix pp 40–42). These sequences were combined with all the available sequences in GISAID to perform the phylogenetic placement (figure 3B). The mean age of the patients was 33·9 years (SD 17·2; range 0–93), with a median of 34 years (21–44). Sex was almost equally represented with 135 (48%) female participants and 145 (52%) male participants (table).

**Table:** Demographic data and lineages in the dataset

	**Sequences (n=280)**	**Variant**	**Distribution**
**Lineage**			
AY.10	2 (1%)	Delta	Few countries worldwide
AY.19	1 (<1 %)	Delta	Few countries worldwide
AY.29*	2 (1%)	Delta	More prevalent in Japan
AY.37†	1 (<1 %)	Delta	More prevalent in Liberia
AY.6	20 (7%)	Delta	More prevalent in Mozambique
B.1	2 (1%)	No assigned variant	More prevalent in Africa and Saudi Arabia
B.1.1	1 (<1 %)	No assigned variant	Worldwide high prevalence in Madagascar
B.1.1.1	1 (<1 %)	No assigned variant	Many countries worldwide
B.1.1.375	13 (5%)	No assigned variant	High prevalence in Mozambique
B.1.1.448	1 (<1 %)	No assigned variant	More prevalent in South Africa
B.1.1.54	1 (<1 %)	No assigned variant	More prevalent in Botswana and South Africa
B.1.351	112 (40%)	Beta	Worldwide
B.1.617.2	117 (42%)	Delta	Worldwide
C.1.1	1 (<1%)	No assigned variant	Only detected in Mozambique
C.1.2†	4 (1%)	No assigned variant	Few countries worldwide
C.6†	1 (<1 %)	No assigned variant	USA, Peru, South Africa
**Sex**			
Female	135 (48%)	..	..
Male	145 (52%)	..	..
**Age, years**			
Median	34 (21–44)	..	..

Data are n (%) or median (IQR). Lineage assignment was performed with Pangolin. Distribution of lineages was obtained from outbreak.info and GISAID. GISAID=Global Initiative on Sharing Avian Influenza Data.

* First reported in Africa, Distribution was obtained from GISAID.

† First reported in Mozambique.

The most prevalent variant in our dataset was delta, especially lineage B.1.617.2, followed by beta (lineage B.1.351). Furthermore, lineages AY.37, C.1.2, and C.6 are reported for the first time in Mozambique; and lineage AY.29 is reported for the first time in Africa (table; appendix p 72). We focused on the introduction and dynamics of beta and delta since they were the most common and circulating variants in Mozambique during the study period.

Beta phylogeny (~34K sequences) took almost a month to be reconstructed with IQ-TREE, while the phylogenetic placement of beta and delta sequences (~87K) took only 10 days with UShER. By comparing both approaches, we obtained an overall match of 88% for introductions, with 319 of 361 sequences equally classified. Regarding topology, the Shimodaira-Hasegawa p value was 0·251, suggesting that UShER and IQ-TREE phylogenies are not significantly different. Most Reconstruct Ancestral State in Phylogenies-defined origins coincide with those obtained with our script (appendix p 74).

We used the beta and delta obtained subtrees to identify groups within Mozambique and unique sequences. For beta, we identified 150 (40%) of 373 sequences distributed in 42 transmission groups, with a size ranging between two and 17 samples (mean, 3·6; median, 2), 33 of which include only cases from Mozambique. Additionally, 145 (39%) of 373 unique sequences were identified. For delta, we identified 324 (58%) of 559 sequences distributed in 49 transmission groups, with a size ranging between two and 97 samples (mean, 6·6; median, 3), 38 of which were exclusively from Mozambique. 171 (31%) of 559 unique sequences were also identified. A proportion of sequences remained unclassified, with 78 (21%) of 373 for beta and 64 (11%) of 559 for delta (appendix pp 43–67). All of them fell on polytomies due to the low SARS-CoV-2 diversity. Additionally, we found a few possible exportations from Mozambique to other countries for both variants. These exports appear as a clade whose basal sample is from Mozambique and have a collection date up to 3 months before the rest of most or all the samples. Beta might have been exported at least once from Mozambique to Slovenia and India, whereas delta might have been exported to the UK at least once (appendix pp 78–79). There are routes to Mozambique in less than 24 h from almost any part of the world, which allows these exportations to occur infrequently. Some of the cases are likely to have occurred via a missing link (a missing case in a country between the country of departure of the virus and its arrival to the country where it is finally reported) according to the phylogenies.

At least 187 independent introductions were identified for beta, most of them with few descendants in Mozambique (90% of transmission groups with less than ten cases from Mozambique and 145 singletons). A higher value was observed for delta with 220 introductions, with a large transmission group including 97 samples, 60 of which were from Mozambique. Nevertheless, most introductions generated few descendants (88% of transmission groups with less than ten cases from Mozambique and 171 singletons). Unclassified samples were not considered in the counting due to limitations in determining the exact origin (appendix pp 43–67).

**Figure 4:**
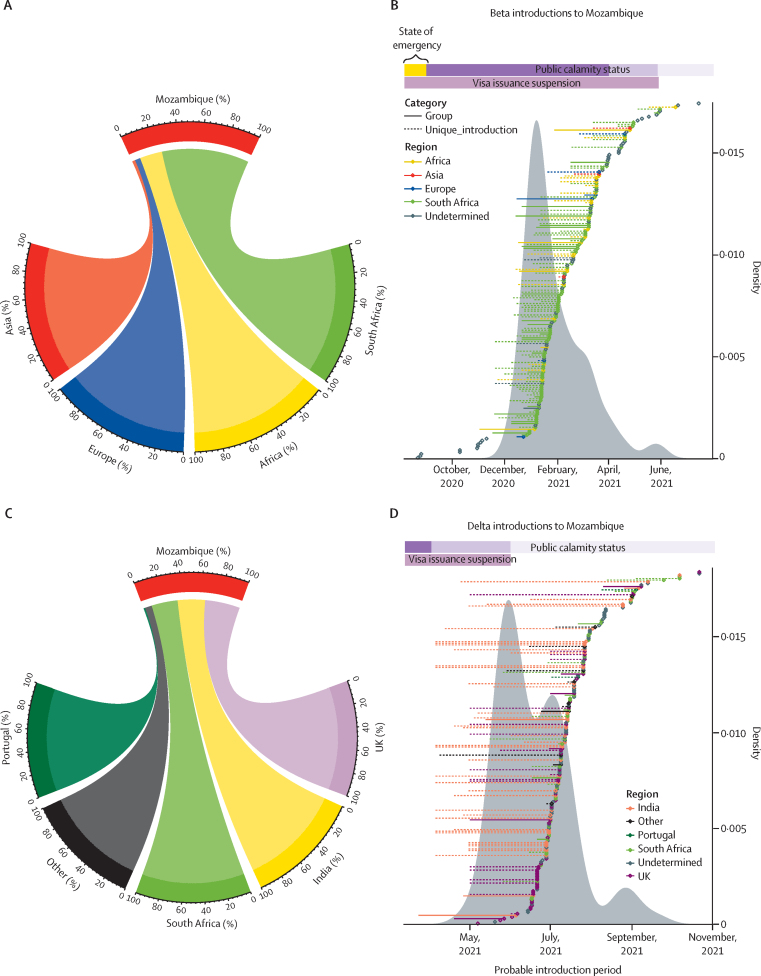
SARS-CoV-2 introductions in Mozambique (A) Circos plot for beta variant. (B) Dot plot for beta variant. (C) Circos plot for delta variant. (D) Dot plot for delta variant. The circos plots indicate the origin of the introductions to Mozambique and the contribution of each country or region. For beta, 74% of introductions came from South Africa, 19% from other African countries, 5% from Europe, and 2% from Asia. For delta, 36% of introductions came from the UK, 28% from India, 27% from South Africa, 1% from Portugal, and 7% from other countries. Dot plots display introduction periods for transmission groups and unique sequences. Density plots indicate introductions distribution. Public calamity status began on Sept 7, 2020, right after the State of emergency. Movement restrictions included: no travel and limited internal movement. During the state of emergency, travels were allowed with mandatory quarantine in Mozambique. Measures were gradually alleviated by April 2 and by May 26, 2021, when internal movement and restricted social events were permitted.

Beta was mainly introduced from South Africa (9 [74%] of 131) and other African countries (25 [19%] of 131), with some introductions from European (six [5%] of 131) and Asian countries (three [2%] of 131; figure 4A). Beta transmission groups mostly arose from South African introductions (26 [62%] of 42) and other African countries (five [12%] of 42). Regarding unique sequences, 71 (49%) of 145 were introduced from South Africa, or other African countries (20 [14%] of 145). The rest were undetermined. The beta variant was probably first introduced around the end of August, 2020, from an undetermined origin, but the wave of continuous introductions happened between November, 2020, and mid-April, 2021, reaching 80% by March 21 (about 7 months after the first introduction; figure 4B). The first case did not generate descendants (unique), but the next two cases generated transmission groups giving rise to a further six and ten cases within Mozambique. The biggest transmission group (n=17; appendix p 43) was introduced early between mid-November, 2020, and January, 2021, from South Africa.

Delta was introduced to Mozambique almost equally from the UK (56 [36%] of 155), India (44 [28%] of 155), and South Africa (42 [27%] of 155); with minor contributions from Portugal (two [1%] of 155) and other countries (11 [7%] of 155; figure 4C). Notably, most transmission groups were introduced from the UK (17 [35%] of 49), including the largest group (97 cases), introduced around mid-April, 2021, and those groups with more than ten cases within Mozambique (5 of 7 groups). However, the smallest groups came from South Africa (13 [27%] of 49) and India (four [8%] of 49), and 27% (13 of 49) had an undetermined origin. Regarding unique sequences, 23% (40 of 171) were introduced from India, 23% (39 of 171) from the UK, 17% (29 of 171) from South Africa, and occasionally from different European or other African countries, or the Americas (11 [6%] of 171). 30% (52 of 171) remained as undetermined origins (appendix pp 52–67).

The first delta introduction occurred by March, 2021, but the majority of introductions took place between May and July, 2021, reaching 80% of total introductions by the end of July (4 months since the first introduction; figure 4D). Notably, uncertainty regarding the introduction date was higher for the first delta introductions coming from India followed by the UK. Few later introductions with shorter periods came from South Africa, followed by Portugal (figure 4D). Considering the high movement of people and connections between Mozambique and Portugal and Mozambique and South Africa, both connected to the UK, we further inspected the waves of delta for those countries (appendix p 80). First, we compared the sequencing effort of the four countries by plotting the number of sequences deposited on GISAID between April, 2020, and October, 2021 (appendix p 80). Delta was first detected in late 2020 in the UK, the country with the highest sequencing effort; then in South Africa on Jan 5, 2021, a country with a similar sequencing effort as Portugal, where it was reported one month before (April 18, 2021) it was identified in Mozambique (May, 2021), the country with the lowest sequencing effort. Additionally, we compared the variants’ profiles obtained from the covariant platform. Variants’ profiles are very similar between Mozambique and South Africa (appendix p 80); and between Portugal and the UK (appendix p 80).

## Discussion

In this study, we analysed the SARS-CoV-2 population structure and dynamics of the waves occurring in Mozambique during 2021. The second wave (January–March, 2021) was mainly caused by the beta variant, whereas the third wave (June–September, 2021) was dominated by delta. We quantified the introductions of both variants, estimated their time span and origin, and evaluated the local transmission and its contribution to fuelling the local pandemic. We used a new tool, UShER, along with a high amount of sequence data publicly available.^19^, ^20^

Almost 190 independent introductions of beta were identified throughout the second wave, with 80% occurring until the end of March, 2021. Most came from South Africa or other African countries, indicating that beta was introduced from the region where the variant was first detected. The few introductions from European and Asian countries occurred at the end of the wave, thus having a low effect on the amount of secondary cases generated. In March–April, 2021, alpha was the dominating variant in Europe, absent in our dataset, while beta prevailed in South Africa and rapidly became dominant in Mozambique by February, 2021,^8^, ^21^ suggesting that international movement restrictions had little effect on stopping introductions into Mozambique from neighbouring countries.^8^, ^13^ Despite the international travel and tourism restrictions, some essential movements between Mozambique and South Africa were allowed (repatriations, transport of essential services or goods, medical needs). Additionally, regional trade in southern Africa returned to pre-pandemic levels by December, 2020,^21^ and clandestine movement between neighbouring countries might also have been a source of introductions.^13^ These results are in concordance with other studies from different settings, which have also found numerous introductions with and without travel bans.^22^, ^23^ A clear example is the early variant 20E (EU1) which was first detected in Spain and was redistributed among other EU countries irrespective of the border measures implemented around August, 2020.^24^

By April, 2021, the Mozambican Government alleviated the internal movement restrictions and, by the end of May, 2021, visa issuance was restored,^25^, ^26^ allowing people to enter the country. Following these events, the third COVID-19 wave started, dominated by delta, the most prevalent variant in the world at that time.^27^, ^28^ We estimated about 220 independent introductions of delta, but with different wave dynamics than beta. Delta introductions happened in a shorter period, with about 80% of them occurring in the first 4 months, by contrast to the 7 months it took for beta introductions to occur. The fast replacement of beta by delta in Mozambique was probably due to the higher transmissibility of delta, with most of the sequences in groups (about 58% *vs* about 31% unique), suggesting elevated community transmission.^29^ The origin of delta introductions showed almost equal contributions from the UK, India, and South Africa. The introductions from South Africa are explained by the connections between both countries, but the links with the UK were unexpected. Notably, the first introductions came from the UK and India and display the highest timing uncertainty which probably reflects the presence of indirect links, due to uneven sampling across countries. Considering the connections between South Africa and Mozambique and the very similar pandemic profile, it is possible introductions linked to the UK entered Mozambique via South Africa, and the lower sequencing effort of South Africa in comparison with the UK prevented the identification of the full link. Notably, before the pandemic, more than 50 000 UK travellers flew monthly to South Africa. These arrivals had decreased to less than 1000 when South Africa reopened its borders in September, 2020, increasing to about 40 000–50 000 during October–December, 2020. South Africa might have acted as a bridge for SARS-CoV-2 entry to the continent, mainly from Europe.^21^ However, this might reflect incomplete sampling in other African countries. Introductions from India are more easily explained by the strong commercial relationship with Mozambique since 2015.^29^, ^30^ Finally, the few introductions from Portugal, observed at the end of the delta wave, could reflect the restrictions imposed by the Portuguese Government between April and June, 2021, when only flights to and from European countries were allowed, with the exception of essential travels and repatriation from African countries, as detailed in the 45-C/2021 and 74-A/2021 resolutions of the Council of Ministers. Additionally, most of the sequences from Portugal were uploaded to GISAID between July and August, 2021.

This study has some limitations. First, new cases were recruited from only one hospital in the Manhiça district. However, the study covered the whole country by using all the sequences from Mozambique available in public repositories. Second, metadata was missing in a considerable number of samples, limiting the integration of genomic and epidemiological data. Third, sampling bias is an insurmountable limitation in this type of study because of the following: (1) sequencing effort is highly different worldwide, thus many origins remained undetermined or were erroneously assigned; (2) low genetic diversity of the virus causes many unclassified cases; and (3) a small proportion of cases, only symptomatic and samples with low Ct values, were amenable for sequencing, making it the most common limitation in SARS-CoV-2 studies. Nevertheless, this has not prevented important inferences being made about virus introductions and spread at different levels.^31^ These limitations might have skewed the observed lineage and phylogenetic patterns, and also underestimated the community transmission. However, contrary to most SARS-CoV-2 studies, we were able to mitigate this issue by analysing all the available genomic evidence, considering millions of sequences, by using UShER. The high concordance between UShER and the reconstructed phylogeny observed for beta, allowed us to confidently use this tool for delta, with about 4 million sequences, which would have otherwise been impossible to analyse due to time consumption and computing resources. We were able to reveal unexpected imports and exports to and from Mozambique, and to narrow down the timing of the events, which is not possible in more sparsely sampled phylogenies. Thus, even with the limitations exposed, our analysis can inform public health interventions regarding management of the variants in the country.

From a public health perspective, Mozambican restrictions in people movement and visa issuance suspensions probably helped avoid the importation of variants, but only from non-African countries, which is supported by the non-detection of alpha in Mozambique even when it was prevalent in Europe during part of the study period. By contrast, beta and delta variants mirrored what was happening in South Africa with some delay. This probably reflects the porosity of the Mozambican borders, particularly with South Africa. Thus, the implemented measures had less health benefits than expected, as variants finally became dominant in the country even though movement restrictions were in place. Finally, thanks to the use of pandemic-scale phylogenies, we were able to reveal unexpected imports and exports to and from Mozambique, and to narrow down the timing of the events.

## Data sharing

Demographic data of the participants are available in the appendix (pp 40–42). Genome sequences are publicly available on GISAID. All scripts used for the analysis, and beta and delta subtree files are publicly available at https://gitlab.com/tbgenomicsunit/mozcovid. The study protocol (appendix pp 1–35) and clinical questionnaire (appendix pp 36–39) will be available with publication.

## Declaration of interests

We declare no competing interests.
