# Supplementary material for: Tracking SARS-CoV-2 introductions in Mozambique using pandemic-scale phylogenies: a retrospective observational study
Source: Lancet Glob Health. 2023 May 16;11(6):e933–41. doi: 10.1016/S2214-109X(23)00169-9 (PMC10187989; doi:10.1016/S2214-109X(23)00169-9)
Supplement: Supplementary appendix [file mmc1.pdf]

THE LANCET
Global Health

Supplementary appendix

This appendix formed part of the original submission and has been peer reviewed. We post it as supplied by the authors.

Supplement to: Martínez-Martínez FJ, Massinga AJ, De Jesus A, et al. Tracking SARS-CoV-2 introductions in Mozambique using pandemic-scale phylogenies: a retrospective observational study. *Lancet Glob Health* 2023; **11:** e933–41.

Coronavirus Disease 2019 Surveillance in Manhiça and Magude Districts, Southern Mozambique for a prompt and effective response against the epidemic Acronym: MozCOVID

PROTOCOL Version#*1.6* – DATE: *10 de Maio*, 2022

Principal Investigator

CISM: Inácio Mandomando, DVM, PhD

Co-Principal Investigator ISGlobal:

Alfredo Mayor, PhD

Co-investigators:

CISM: Sérgio Massora, PhD; Nelson Tembe, PhD; Tacilta Nhampossa, MD, PhD; Francisco Saute, MD, PhD; Eusebio Macete, MD, PhD; Delfino Vubil, PhD; Khatia Munguambe, PhD; Ariel Nhacolo, MSc; Augusto Messa Junior, DVM; Chenjerai Jairoce, MSc; Arsénia Massinga, MSc; Pio Vitorino, MD; António Sitoe, MSc; Áuria de Jesus, MD; Amilcar Magaço, BSc; and Contardo Ambrósio, BSc.;

Instituto Nacional de Saúde: Sónia Enosse, PhD; Rufino Gujamo, PhD

ISGlobal: Quique Bassat, MD, PhD; Azucena Bardaji, MD, PhD; Clara Menendez, MD, PhD; Llorenç Quintó, MSc; Carlota Dobaño, PhD; Alberto García-Basteiro, MD, PhD; Raquel González, MD, PhD; Caterina Guinovart, MD, MSc, PhD

OMS: Jason Mwenda Mathiu ; Carlos Funzamo MD, MIPH; Catherine Hannah Lewis ; Isabel Bergeri.

Imperial College: Patrick Walker, PhD

FUNDERS:

Emory Global Health Institute through Child Health and Mortality Prevention Surveillance (CHAMPS)

LIST OF INVESTIGATORS AND THEIR RESPONSIBILITIES

No. order	Investigators	Responsibilities
1	Inacio Mandomando	Protocol writing,<br>Overall study implementation and supervision Data analysis, Write & Review of manuscript and reports
2	Alfredo Mayor	Protocol writing, overall study supervision, data analysis, and manuscript writing and review
3	Tacilta Nhampossa	Protocol writing, overall study support, data analysis, and assistance with manuscript writing and review
4	Delfino Vubil	Laboratory study coordination and sample processing
5	Augusto Messa Jr.	Laboratory study coordination and sample processing
6	Arsénia Massinga	Laboratory study coordination and sample processing
7	Pio Vitorino	Clinical study coordination, data collecting and cleaning
8	Ariel Nhacolo	Protocol review and overall demography data support
9	Amilcar Magaço	Social component
10	Khátia Munguambe	Protocol writing, overall study support, data analysis, and assistance with manuscript writing and review
11	Arsénio Nhacolo	Data manager and analysis
12	Francisco Saúte	Protocol writing, overall study support, data analysis, and assistance with manuscript writing and review
13	Eusébio Macete	Protocol writing, overall study support, data analysis, and assistance with manuscript writing and review

14	Augusto Messa Junior	Protocol writing, overall study support, data analysis, and assistance with manuscript writing and review
15	Chenjerai Jairoce	Luminex expertise
16	Quique Bassat	Protocol writing, overall study support, data analysis, and assistance with manuscript writing and review
17	Azucena Bardají	Protocol writing, overall study support, data analysis, and assistance with manuscript writing and review
18	Clara Menendez	Protocol review, overall technical support and assistance with manuscript writing and review
19	Raquel González	Protocol writing, overall study support, data analysis, and assistance with manuscript writing and review
20	Llorenç Quintó	Statistics analysis
21	Patrick Walker	Modelling
22	Sérgio Massora	Protocol writing, overall study support, data analysis, and assistance with manuscript writing and review
23	António Sitoe	Clinical study coordination, data collection and cleaning
24	Áuria de Jesus	Clinical study coordination, data collecting and cleaning
25	Sónia Enosse	Data sharing and dissemination with Ministry of Health and Other stakeholders
26	Rufino Gujamo	Data sharing and dissemination with Ministry of Health and Other stakeholders
27	Jason Muthui	Overall study support, assistance in cross-sectional component
28	Catherine Lewis	Overall study support, assistance in cross-sectional component
29	Carlos Funzamo	Overall study support, assistance in cross-sectional component

30	Isabel Bergeri	Overall study support, assistance in cross-sectional component

LIST OF COLLABORATORS INSTITUTIONS INVOLVED IN THE STUDY

N. Orden	INSTITUTION	RESPOSANBILITIES
1	Hospital Distrital de Manhiça, Maputo, Moçambique	COVID-19 surveillance site (Children and Adults enrolment)
2	Centro de Investigação em Saude da Manhiça, Maputo, Mozambique	Study implementer
3	ISGlobal, Barcelona Institute for Global Health, Hospital Clínic - Universitat de Barcelona, Barcelona, Spain	Overall Epidemiological, Laboratory and statistical analysis support
4	Imperial College (London)	Modelling
5	Instituto Nacional de Saúde (INS), Maputo, Mozambique	Data sharing and dissemination

ABREVIATIONS

AIDS - Acquired immunodeficiency syndrome

BMGF - Bill & Melinda Gates Foundation

CDC - Center for Disease Control and Prevention

CISM - Centro de Investigação em Saúde de Manhiça

CHAMPS - Child Health and Mortality Prevention Surveillance

COVID-19 - Coronavirus Disease 2019

FIEBRE - Febrile illness evaluation in a broad range of endemicities

FNI - Fundo Nacional de Investigação

HDSS - Health Demographic Surveillance System

GAVI - Global Alliance for Vaccines and Immunization

HIV - Human immunodeficiency virus

IRB - Institutional review board

MDH - Manhiça District Hospital

MITS - Minimally Invasive Tissue Sampling

PCR - Polymerase chain reaction

RSV - Respiratory syncytial virus

SARS-CoV-2 - Severe acute respiratory syndrome coronavirus 2

TB - Tuberculosis

USAID - United States Agency for International Development

WHO- World Health Organization

TABLE OF CONTENTS

1. PROTOCOL SUMMARY 6

2. CONFIDENTIALITY STATEMENT 7

3. INTRODUCTION 8

4. OBJECTIVES 9

5. METHODOLOGY 10

5.1. Project Setting 10

5.2. Definitions 12

5.3. Project Population 13

5.4. Inclusion Criteria 13

5.5. Exclusion Criteria 14

5.6. Study Design 14

5.7. Laboratory Sample Collection and Processing 17

5.8. Patient follow up for outcome determination in prognostic biomarker component **……….…**

5.9. Data Collection Forms, Data Entry and Data Analysis 20

5.10. Sample Size Estimation 22

6. CAPACITY BUILDING 24

7. RESULTS DISSEMINATION 24

8. PROTECTION OF HUMAN SUBJECTS 24

9. BUDGET 26

10. TIMELINE 27

11. REFERENCES 28

12. APPENDICES 30

1. PROTOCOL SUMMARY

The current Coronavirus Disease 2019 (COVID-19) pandemic is the paradigmatic example on how infectious diseases may menace the world's health and economy, and particularly contribute to enhance current inequities in health related to wealth. Although Mozambique has so far had few confirmed cases of COVID-19, it is unknown if low testing rates and other factors such ***as*** the warm climatic conditions may explain the apparently low spread of the virus. The present study aims to rapidly gain an understanding of the epidemic curve and natural history of severe acute respiratory syndrome coronavirus 2 (SARS-CoV-2) in Manhiça district, a rural area in southern Mozambique, through a series of epidemiological indicators obtained at both the health facility and community levels, so as to provide evidence based recommendations for managing and mitigating the COVID19 epidemic. Age-specific incidence of severe COVID-19 obtained at health facilities during a period of 6 months together with seroprevalence data obtained from the community will be used to determine SARS-CoV-2 reproduction number and effects of interventions through mathematical modelling. Viral, serological and clinical progression will be assessed in a longitudinal cohort of symptomatic SARS-CoV-2 infected individuals. Community awareness of COVID-19 and of project aims will be fostered through engagement activities among community leaders and members. COVID-19 surveillance capacities in Mozambique will be strengthened by developing SARS-CoV-2 diagnostic approaches (molecular testing and an innovative immunoassay to detect antibodies against the virus), as well as SARS-CoV-2 genome sequencing approaches to identify transmission chains. Finally, the project will pursue the creation of a bank of serum, virus materials and data which will be open for immediate public health purposes, including the development of point-of-care diagnostic tests.

2. CONFIDENTIALITY STATEMENT

The information presented in this protocol is confidential. Therefore, it should not be disclosed or shared with third parties without specific permission provided in writing by the principal

investigators or when disclosure is required by regulatory authorities. This disclosure restriction shall also apply to information provided at a later date that is privileged or confidential.

Once printed and signed by the investigator(s) and other authorized subscribers, the protocol cannot be changed informally. Protocol amendments have the same need for approval and must follow all mandatory steps to be reviewed and approved before they are implemented.

By signing this document, the researcher undertakes to carry out the study according to the protocol, the applicable guidelines according to the Helsinki Declaration and in a manner consistent with international scientific standards, as well as all applicable regulatory requirements. The researcher also undertakes to demonstrate efforts to carry out the study within the designated deadlines.

Principal Investigator

Name PI: ______________________________

Signature: ______________________________

Date: ____________

3. INTRODUCTION

It has been estimated that an unmitigated epidemic would lead to 17.6 million people needing hospitalization, 3.2 million of which sufficiently severe to need critical care (1). Although Sub-Saharan Africa has so far had few confirmed COVID-19 cases compared with most other parts of the world, with the number ranging from thousands (South Africa), to few hundreds (Cameroon and Burkina Faso) or dozens (Mozambique) by April 2020 (2), it is expected that the pandemic will hit the continent with unprecedented virulence and impact. Some factors that may explain the apparently low spread of the virus are the low level of urbanization, the warm climatic conditions, the very different population pyramids to those of China or Europe, the delays in the arrival and spread of cases in the continent giving countries more time to prepare, as well as a clear lack of testing capability. However, now that the virus has made its way into Africa, countries need to cautiously prepare for greater action to contain its spread, especially if it follows a seasonal pattern.

The fragility of the health systems in Africa, particularly in periurban and rural areas, is an enormous matter of concern with regards to how the countries will be able to deal with severe COVID-19 cases, and design their containment and emergency responses. Importantly, the previous experiences of the Ebola outbreaks point to the need for countries with high burden of co-existing infectious diseases (malaria, TB, HIV etc.) to consider preventive measures against not only the COVID-19 threat but also its likely impact on existing infectious diseases control efforts. The detection and spread of an emerging respiratory pathogen such as SARS-CoV-2 are accompanied by uncertainty over the key epidemiological, clinical and virological characteristics of the novel pathogen and particularly its ability to spread in the human population and its virulence. Research on early identified cases has led to estimates of key epidemiological parameters of COVID-19 disease, such as the basic reproduction number (2.2) (3), delays between infection and illness onset (5–6 days) (4), and case fatality ratios (1.414% among hospitalized cases (5; 6; 7). Case series studies (8) have illustrated different clinical and biological types of evolution of COVID-19 infection and disease, including symptomatic cases with high viral load, young mild cases progressing to pneumonia and severe disease by days 10–11, and older patient with a rapid evolution towards critical disease with a long and sustained persistence of SARS-CoV-2. Several factors could affect these epidemiological estimates, especially in contexts where interactions with other infectious (i.e., malaria) and non-infectious conditions (i.e, malnutrition) can affect the risk of infection and disease progression.

Therefore, there is a need for further research to more accurately characterize estimates for the epidemiological parameters underlying the transmission dynamics of COVID-19 in sub-Saharan Africa.

4. OBJECTIVES

Aim

The overall goal of this project is to rapidly gain an understanding of the epidemic dynamics and natural history of SARS-CoV-2 in Southern Mozambique through the evaluation of epidemiological clinical and laboratory-based indicators obtained at both the health facility and

community levels, so as to provide evidence-based recommendations for managing and mitigating the COVID19 epidemic in Manhiça, southern Mozambique.

Primary objectives

1. To set up a COVID-19 passive surveillance system at the health facility level to estimate age-specific incidence of COVID-19 cases and evaluate epidemic dynamics in the Manhiça area.
2. To determine the age-specific seroprevalence of IgM and IgG antibodies against SARS-CoV-2 infection in the general population at baseline and 6 months later.

Secondary objectives

1. To describe SARS-CoV-2 clinical presentation and associated mortality in all age patients, in the context of the existing co-morbidities endemic in the country.
2. To describe the viral, serological and clinical progression of symptomatic SARS-CoV-2 infected individuals, and to assess the potential prognostic capacity of a variety of plasmatic biomarkers on arrival in terms of predicting oxygen need and adverse outcomes.
3. To estimate SARS-CoV-2 basic reproduction number using mathematical modelling based in demographic Surveillance System (DSS) and surveillance data.
4. To establish a biorepository of viral RNA and plasma samples for future characterization of COVID-19 disease or immune response when new tools become available.
5. To track local transmission versus imported cases and identify local transmission chains through the whole genome sequencing of SARS-CoV-2 isolates.

5. METHODOLOGY

5.1. Project Setting

The study will be conducted by the Manhiça Health Research Centre (CISM) in the Manhiça District, a semi-rural area Southern Mozambique. The CISM has been conducting research there for more than 20 years which will facilitate the implementation of the project.

Since 1996, the CISM has been running a Health Demographic Surveillance System (HDSS) in the population living in Manhiça. Initially, the area under demographic surveillance was covering less than half of the district population. From 2014, the area under demographic

surveillance was expanded to cover the entire Manhiça district. Manhiça district has a total population of 204,357 and 47.061 households and 27.560 children under five years (CISM demography unpublished data). Each person living within the DSS study area is issued a unique Permanent Identification number that describes the geographic localization of his/her household. Some demographic indicators in 2015 are: birth cohort of 3,946, life expectancy at birth 51 years, neonatal, infant and under five mortalities are 22.5/1,000 live births, 55.8 and 99.0, respectively. Children under the age five represent 18% (29,700) of the total population (9).

In 1998, CISM established a morbidity surveillance system at Manhiça District Hospital (MDH) and five other peripheral health posts (10), to document paediatric morbidity and mortality. Morbidity surveillance includes the systematic collection (using standardized forms) of demographic, clinical history, clinical exam, outcome and treatments for all children <15 years of age visiting the outpatient department or being admitted to hospital. Data on over 75,000 paediatric admissions and > 1.2 million outpatient visits have been collected over the past 18 years. As part of this morbidity surveillance, malaria screening (for all children with documented fever >=37.5ºC or a history of fever in the preceding 24 hours) and microbiological surveillance are also routinely conducted, and blood cultures are systematically collected for all admissions <2 years of age, and for older children with suspected severe disease. The COVID-19 will be integrated in the ongoing CISM morbidity surveillance.

Additionally, CISM has other ongoing studies that will serve as a platform for COVID-19 surveillance, namely:

Respiratory syncytial virus (RSV) infection in children and pregnant women in the Manhiça District (RESPIRE study): This is a health facility-based prospective observational study aimed to investigating the epidemiology of RSV, funded by La Marató TV3 Foundation using passive surveillance in all under-five children hospitalized with WHO-defined clinical criteria for severe pneumonia, and all pregnant women attending the outpatient, inpatient and ANC services with clinical signs of acute upper or acute lower respiratory infections.

Febrile illness evaluation in southern Mozambique (FIEBRE study): Patients (inpatient and outpatient) of all ages (two months and older) who present with fever at the Manhiça

District Hospital are being included in this platform, funded by the UK Aid agency (DFID), with the objective of identifying the causes of fever, and the antimicrobial susceptibility of bacterial pathogens causing fever. Patients are being recruited if they present with tympanic or axillary temperature ≥37.5°C.

Child Health and Mortality Prevention Surveillance (CHAMPS): The CHAMPS study (funded by BMGF) in Manhiça has been prospectively conducting mortality surveillance in stillbirths and children <5 for the past 3 years, using the innovative minimally invasive tissue sampling (MITS) post-mortem technique. We aim to expand the population under study to in-hospital adult deaths, either through MITS or a simplified postmortem oral/nasopharyngeal swabs

PCV impact assessment for pneumococcal disease (PNEUMO-PCV): CISM has carried out surveillance for invasive bacterial diseases, including *pneumococcus,* in children <15 years of *age* since 2001 and for paediatric pneumonia < 5 years of age since 2004 at MDH.
Since 2019, this study includes pneumonia surveillance in all adults ≥15 years of age admitted to hospital with clinical signs of severe pneumonia.
This surveillance has been used for vaccine impact evaluations. It has been supported by funds from several partners such as Gavi, The Vaccine Alliance, USAID Mozambique mission, the Center for Disease Control and Prevention (CDC) and BMGF.

CISM is adjacent to Manhiça District Hospital (MDH). The hospital has a maternity ward, a surgery room (where caesarean sections can be performed, together with basic emergency surgery), a fully digital (film-free) X-ray machine, and a clinical trials unit. It has been estimated that around 85% of the deliveries in the area (+/-5000 per year) are institutional deliveries, and a facility ("waiting home") is available at MDH for pregnant women with risk factors for a complicated delivery to settle by the hospital in attendance of labour, facilitating a supervised delivery. MDH's morgue, which has been recently refurbished, is where Minimally Invasive Tissue Sampling (MITS) procedures are routinely conducted for deaths occurring at the Manhiça District, as part of the Child Health and Mortality Prevention Surveillance (CHAMPS) project general procedures. The morgue has two dissection tables, and 9 body fridges for the conservation of corpses.

5.2. Definitions

COVID-19 Case definition and identification (11; 12)

Suspected case requiring diagnostic testing

1) A patient with acute respiratory tract infection (sudden onset of at least one of the following: cough, fever, shortness of breath) AND with no other aetiology that fully explains the clinical presentation AND with a history of travel or residence in a country/area reporting local or community transmission during the 14 days prior to symptom onset;

OR

2) A patient with any acute respiratory illness AND having been in close contact with a confirmed or probable COVID-19 case in the last 14 days prior to onset of symptoms;

OR

3) A patient with severe acute respiratory infection (fever and at least one sign/symptom of respiratory disease (e.g., cough, fever, shortness breath) AND requiring hospitalization (SARI) AND with no other aetiology that fully explains the clinical presentation.

Probable case

A suspected case for whom testing for virus causing COVID-19 is inconclusive (according to the test results reported by the laboratory) or for whom testing was positive on a pancoronavirus assay.

Confirmed case

A person with laboratory confirmation of virus causing COVID-19 infection, irrespective of clinical signs and symptoms.

COVID-19-associated in-hospital mortality

The proportion of cases of COVID19 condition that are fatal within a specified time per total of confirmed cases.

5.3. Project Population

The COVID-19 surveillance will include persons residing in the Manhiça HDSS area.

5.4. Inclusion Criteria

The study has five components and therefore the inclusion criteria may vary from one component to another.

a) COVID-19 morbidity surveillance

- Individuals of any age admitted to MDH or pregnant women attending the Maragra health centre meeting the COVID-19 case definition as defined above after the project start date.
- Resident of Manhiça DSS area
- Written informed consent from a parent or guardian if a child, and from participant if an adult prior recruitment.

b) COVID-19 mortality surveillance

- All deaths in children under the age of 5 (including stillbirths), occurring at the health facility or at the community;
- In hospital adult deaths occurring at the MDH;
- Resident of Manhiça DSS area;
- Written informed consent from the relative of the dead person.

c) COVID-19 population-based serosurveys

- Individuals of all age groups residing in Manhiça DSS surveillance area
- Signed informed parental permission from a parent or guardian if a child and from participant if an adult.

d) Natural history of SARS-CoV-2 infection

- Participants who fulfil eligible criteria for activity a) and with laboratory confirmed SARS-CoV-2 infection.

e) Clinical presentation and factors associated with SARS-CoV-2

- Participants who fulfil eligible criteria for activity a) and c).

5.5. Exclusion Criteria

- Participation in an interventional clinical trial at the time of enrolment.
- For the biomarker component, patients <18 years of age .

5.6. Study Design

COVID-19 morbidity surveillance

The study will be conducted through the passive detection of severe COVID-19 cases during a period of 6 months. All patients (or parents/guardians on behalf of minors) attending the Manhiça District Hospital (MDH) and pregnant women attending the Maragra Health Centre, with acute respiratory syndrome fulfilling severity criteria will be invited to participate in the study. Written informed consent will be obtained from patients (or parents/guardians on behalf of minors) prior to recruitment. Assent will be requested for parents or caretaker of participants aged 15-18 years old.

Pre-screening of patients attending sentinel health care will be carried out at the points of entry of the health unit, separately for adults and children. At each entry point, patients will be screened and then classified according to the WHO definition of a suspected case. In these places, the "cough agent", receptionist or other personnel responsible for the reception will pre-screen all individuals seeking for health care for any reason (consultations, pharmacy and SMI, PAV, etc.). Individuals without respiratory symptoms will follow the normal circuit already known, separated from patients with respiratory symptoms. Patients with respiratory signs or symptoms will be taken to the waiting area for further observation at the "COVID office". In this office, additional screening for COVID-19 and medical consultation will be carried out. The additional screening will consist of filling out a specific form to identify suspected cases of COVID-19. This form (in physical or electronic format), after being properly filled out, must be sent to CISM.

Regarding Child Morbidity Surveillance, the outpatient form (OPD) will be completed first by the receptionist to collect identification and demographic data. Then, the clinical component will be completed by clinicians in both offices (COVID and normal offices).

In patients with symptoms suggestive of COVID-19, on an outpatient basis, the identification of their contacts should be carried out, as well as the collection of samples from pre-identified places in the health unit. These samples must be sent to the CISM laboratory for COVID-19 testing in accordance with MISAU standards.

Patients with respiratory symptoms who present with signs of severity should be sent to a specific location, along with other patients on the respiratory forum. In these patients, in case of clinical suspicion of COVID-19, testing for COVID-19 should be performed. While waiting for the results, these patients must remain isolated and receive hospital treatment according to their clinical condition as recommended by MISAU. If the test is positive, patients should be transferred to referral centres for the treatment of COVID-19. If this is not possible, they should be isolated in pre-identified places at the health unit level. Patients with a negative test should follow normal clinical treatment according to their clinical condition.

In peripheral health facilities due to the small number of health professionals and limited space, all patients will be seen in the same office. Clinicians should observe all patients with respiratory symptoms using personal protection equipment (PPE) and ensure an airy environment in the room. Pre-screening should be done to separate the two groups in the waiting room. Patients with respiratory symptoms will be prioritized. During the consultation period, the clinician must frequently track newly arrived patients to the health unit.

COVID-19 mortality surveillance

All deaths in children under the age of 5 (including stillbirths), occurring at the health facility or at the community will be eligible for MITS, as part of the CHAMPS routine activities, and as such will be approached for consent. Moreover, in hospital adult deaths occurring at the MDH will have a much simpler post mortem procedure to screen for SARS-CoV-2 infection, based on obtaining a nasopharyngeal swab if no MITS agreed, but full MITS if agreed within

the first hours after death. Written informed consent from the relative of the dead person prior to any study procedure.

COVID-19 population-based serosurveys

The extent of age-specific infection and the fraction of asymptomatic infection will be estimated through four cross-sectional seroprevalence community surveys, one at the beginning of the study and the 2 after will have intervals of 2 months between (or at least 30 days), when we estimate most of the cases will have already occurred (Figure 4 in Annex), to monitor changes in transmission. The ***fourth cross-sectional will be implemented with an additional follow-up group of the seropositive participants from the third cross-sectional to evaluate the duration of SARS-CoV-2 antibodies previously detected in plasma***The sample of individuals from the community will be recruited using existing demographic surveillance system (DSS) infrastructure by generating a list of randomly selected people within the age group of interest. The list of potential participants will include the target sample size + 20% in case replacement is necessary due to potential refusals.

Natural history of SARS-CoV-2 infection

A cohort of SARS-CoV-2 acutely infected (RT-qPCR confirmed) symptomatic and asymptomatic individuals will be followed-up during 6 months. Participants of the activity a) mentioned above, with positive for SARS-CoV-2 in the RT-qPCR, as well as a same number of positive individuals with no symptoms at recruitment will be included in the sub-study. We will adapt the open-access questionnaires and procedures from the "International Severe Acute Respiratory and emerging Infection Consortium" ***(https://isaric.org/)***, supported by WHO (36), which will be updated in response to COVID-19.

Clinical presentation and factors associated with SARS-CoV-2

Sero-positivity, severe COVID19 and COVID-19-associated in-hospital mortality in Mozambican all-age patients will be analysed in the context of the existing comorbidities

endemic in the country (malaria, TB, HIV, malnutrition, etc.). The information collected through the hospital surveillance (activity a) above) and the community surveys (activity c) above), which will include sociodemographic information, signs and symptoms, comorbidities (including malaria infection) and ongoing medication will be analysed to 1) describe the clinical presentation, and 2) assess factors associated with severe COVID-19 cases sero-positivity and COVID-19-associated in-hospital mortality. These data will be complemented with information of community-based mortality captured through the DSS in placed at the Manhiça District, because were such data believed to under-represented in confirmed deaths at hospitals and health centres. In addition, we will assess on arrival a variety of plasmatic biomarkers with known predictive capacity for other respiratory infections, with the intention to evaluate their prognostic capacity to predict oxygen requirements, admission, severity and adverse outcomes.

***Rationale for the biomarker component:** Having the ability to prognosticate the need for supplemental oxygen (the main treatment available in a setting such as Manhiça) through measurement of parameters available at the time of arrival at a healthcare facility would strengthen the capability to identify those patients presenting with moderate symptoms that can be safely discharged away from the facility. Based on recent estimates, only 20% of all symptomatic patients with COVID-19 develop a requirement for supplemental oxygen.

Although existing prognostic scores have yielded disappointing results in patients with SARS-CoV-2 infection, a number of demographic, clinical and laboratory parameters are associated with a more severe disease course and worse patient outcomes. However, to our knowledge few studies have examined the performance of prognostic markers in patients who do not require supplemental oxygen at presentation. Only one of these studies included outpatient or ambulatory care settings and none were conducted in resource-limited settings. Hence, whether measurement of these parameters can inform the decision to safely discharge a patient away from a health facility is as yet unclear. We propose to assess the following biomarkers (see table) easily detectable in plasma to evaluate their prognostic role in COVID-19:

**Of note: The prognostic biomarker component will only be assessed in adult participants aged 18 years or more, which are positive for SARS-CoV-2 infection and that were previously selected to the Natural History component.

Biomarker	Evidence in COVID-19	Stage of development for near-patient test
**Neutrophil:Lymphocyte ratio (NLR)**	Multiple studies have identified elevated neutrophil counts and/or decreased lymphocyte counts as poor prognostic indicators in patients with COVID-19.[4,9,20]	Quantitative near-patient tests such as the HemoCue WBC DIFF can measure differential leukocyte counts using 10µL of whole blood, with a turnaround time of 5 minutes.
**C-reactive protein (CRP)**	CRP has been found to be a poor prognostic indicator in hospitalised patients with COVID-19 in China and Germany.[9,10,21] Elevated CRP is being used to enrich study populations in trials of novel therapeutics (e.g. TACTIC trial: ISRCTN 11188345) and to inform access to newly licensed treatments (e.g. Remdesivir, UK MHRA).	Numerous commercial POCTs exist for quantitative measurement of CRP, for example the Nycocard CRP test manufactured by Alere, which has a 3 minute turnaround time and requires 5µL of whole blood. It is widely implemented in many resource-limited settings.
**D-dimer**	Coagulopathy, including multi-system arterial and venous thromboses, is emerging as an important component of COVID-19 pathophysiology. Several studies have established D-dimer as a predictor of poor outcomes in hospitalised patients with COVID-19 and measurement of D-dimer is recommended by the International Society of Thrombosis and Haemostasis in the triage of COVID-19 patients.[4,22-24]	Several commercial D-dimer POCTs are available. The RAMP D-dimer test is a quantitative lateral flow assay with a turnaround time of 15 minutes that can be run on whole blood. The DIAcheck C2 machine has also been validated for use in LMIC contexts.
**Procalcitonin (PCT)**	A number of studies including a recent meta-analysis have indicated that patients with elevated PCT levels at admission are more likely to have severe COVID-19 infection and require ICU admission, although questions remain as to whether raised PCT reflects the severity of SARS-CoV-2 infection or secondary bacterial infections.[25-27]	There are a number of near-patient tests available for measurement of PCT that are compatible with whole blood samples and can deliver a quantitative result within 15 minutes.
**Soluble urokinase-type plasminogen activator receptor (suPAR)**	suPAR has demonstrated prognostic utility in adults with COVID-19 in the UK, Denmark and Greece. It has been shown to identify patients at high risk of respiratory failure and those safe for discharge from hospital.[11,28,29]	The suPARnostic® Quick Triage Test which is commercially available from Virogates measures suPAR quantitatively. The test needs 10µL of plasma. Each assay delivers a quantitative result in 25 minutes and costs USD 20.
**Interleukin-6 (IL-6)**	Several studies, including a recent meta-analysis, have demonstrated that elevated IL-6 levels predict the risk of severe COVID-19 infection, ARDS and respiratory failure.[4,10,27,30] In addition, IL-6 was identified as the best predictor of oxygen requirement in a cohort of Swiss patients (van Singer, in submission). Many clinical trials are investigating the utility of anti-IL-6 therapy in patients with COVID-19.	Quantitative IL-6 POCTs are available, for example the Milenia QuickLine IL-6 rapid lateral flow assay. Each test costs 20 USD, requires 100µL of plasma or serum and has a 20 minute turnaround time. The sensitivity of the Milenia IL-6 test is 50pg/mL, however some studies suggest that the threshold for predicting severe COVID-19 might be lower (e.g. 35pg/mL). Prototypes for other rapid assays are currently being evaluated.[31]
**Soluble triggering receptor expressed on myeloid cells-1 (sTREM-1)**	sTREM-1 has been shown to predict respiratory failure in hospitalised patients with COVID-19 (van Singer, in submission).	Prototype quantitative lateral flow assays for sTREM-1 are being developed (FIND, personal communication).

Table 1: Table 1. Biochemical biomarkers for batched retrospective measurement in the study, and stage of readiness for point-of-care or near-patient tests. Green = test in routine use in LMIC settings; Orange = test available but not in routine use in LMIC settings; Red = prototype test available. A number of other biochemical markers (IL-1Ra, IL-8, IL-10, CXCL-10/IP-10 and Ang-2) have been identified as poor prognostic indicators in patients with COVID-19. Whilst near-patient tests do not yet exist for these markers, a number of them have shown promise as early markers of severity in patients with SARS-CoV-2 infections and we will include these on the multi-analyse panel used in this study.

5.7. Laboratory Sample Collection and Processing

5.7.1. Sample Collection

Using standardized methods, a nasopharyngeal (NP) swab will be collected from all children (2 months -14 years old) and adults, including pregnant women, at enrolment according to standard methodology. In infants less than 2 months of age a nasopharyngeal aspirate (NPA) will be collected using NPAK kits (M-Pro, Michigan, USA). Detection of SARCV-CoV-2 will be performed by RT-qPCR.

A Capilary blood, 500 uL will also be collected and placed in microtainer EDTA (13; 14) and will send to CISM laboratory for detection of IgM and IgG against SARS-CoV2.

To study prognostic biomarkers, a venous 10mL blood sample will be collected among participants attending the hospital and subject to clinical evaluation. To study the natural history of SARS-CoV-2 infection, a nasopharyngeal swab as well as fingerprick or venous blood (8ml) will be collected as indicated in the table below to detect and quantify viral load as well as IgM and IgG antibody titers against the virus and cellular immune responses. A peripheral blood sample will be collected at month 3 to 6 to assess the longevity of antibody responses. If feasible, saliva, urine and stool samples will be also collected, and together with blood fractions (plasma and immune cells) will be stored in the sample biorepository at CISM for COVID-19 research. Asymptomatic patients with two RT-PCR negative nasopharyngeal samples at least 48 h apart will be discharged. The sampling will be performed at the Manhiça District Hospital for admitted patients or in the household for asymptomatic participants using adequate personal protective equipments for both healthcare and participants

	Month									
	1				***2***	***3***	***4***	***5***	***6***	***If qPCR+****
	d0	***d7***	***d14***	***d28***	***d56***	***d84***	***d112***	***d140***	***d168***	
Nasopharyngeal swab	***X***	***X***	***X***	***X***	***X***	***X***	***X***	***X***	***X***	***X***
Venous blood (8 ml)	***X***			***X***					***X***	
Venous blood (10mL) for biomarkers (only adults)	***X***									

Fingerprick (microtainer)		***X***	***X***		***X***	***X***	***X***	***X***		
Saliva	***X***	***X***	***X***	***X***	***X***	***X***	***X***	***X***	***X***	***X***
Faeces					***X***	***X***			***X***	
Outcome ascertainment (possible through phone call)		***X***	***X***							
Extraction of information from clinical records	***X***	***X***	***X***							

***7 days after any positive PCR**

MITS will be performed among hospitalized children with poor outcome. Nasopharyngeal swab and lung biopsy will be collected and shipped to CISM for laboratory for processing as previously described (15).

5.7.2. Sample Processing

<u>Detection of SARCV-CoV-2 by RT-PCR</u>

After extraction viral RNA from nasopharyngeal swabs using the QIAamp Viral RNA Mini Kit (QIAGEN), the TaqMan SARS-CoV-2 Assay Kit v2 (Thermo Fisher) and the Applied Biosystems 7500/7500 Fast Real Time PCR platforms will be used for the detection of viral RNA. This TaqMan® probe based multiplex RT-qPCR assay targets SARS-CoV-2 Orf1ab, S (spike) and N (nucleocapsid) genes, as well as the human RNase P gene (internal control). The multiplex format allows a reduction in the number of reactions to be set-up per sample, and increases the number of samples that can be tested in a single run (in the singleplex protocol, 4 wells are required per sample to test for all targets individually). To increase the throughput of the RT-qPCR, a pooling strategy will be considered after estimating the effectiveness of such strategy based on the expected viral positive rate and sensitivity of the approach.

<u>SARS-CoV2 genome sequencing</u> rRNA obtained from patient samples confirmed by RT-qPCR (those with Ct values below ***27*** to ensure enough viral RNA for sequencing) will be retrotranscribed using random hexamers. cDNA will be then amplified using 2 overlapping

primers pools to guarantee the maximum genome coverage. Depending on the number of viral isolates, we will use Oxford Nanopore Technologies (MinION) third generation sequencers or second-generation Illumina sequencers (MiSeq) available in the Institute of Biomedicine of Valencia, IBV-CSIC (Dr Iñaki Comas, Spain), which leads the Spanish sequencing effort for SARS-CoV-2, the later having a longer work-around preparation time (of about 5 days) but a huge multiplexing power. Primary data analysis will be carried out using Artic Network bioinformatics protocol (https://artic.network/ncov-2019), a global initiative funded by the Wellcome Trust which is making available molecular biology and bioinformatic protocols. Reads will be mapped against the last reference genome (SARS-CoV-2 isolate Wuhan-Hu-1. GenBank: MN908947.3), followed by variant and consensus calling. The consensus genome sequence of the virus obtained will be aligned and compared against reference sequences from a global database (GISAID) using the Nextstrain workflow to identify imported versus local transmission events. Local transmission chains will be identified as monophyletic groups consisting in very closely related genomes all belonging to Mozambique cases. Phylogenetic dating and phylodynamic approaches will be used to identify the earliest introduction in Mozambique and the age of the local transmission groups. The genomic data produced will be shared with the community through public repositories and uploaded to GISAID coupled with automatic analysis in Nextstrain.

For post mortem diagnosis MITS will be performed under the CHAMPS protocol as described elsewhere (16). Briefly, the body is transported to a designated MITS procedure room within a local facility, and a series of tissue (i.e, both lungs,) and nontissue specimens (i.e, blood, respiratory secretions via nasopharyngeal/oropharyngeal swabs) are collected.

<u>Biomarker panel determination for prognostic determination</u>

Venous blood samples will be collected via venepuncture upon recruitment by research staff. The maximum total blood volume collected will be 10 ml. Samples will be collected in EDTA tubes and stored at 2-8°C. Full blood count samples will be batched and run retrospectively within 24 hours. Biochemical biomarker samples will be stored for a maximum of four hours prior to centrifugation. Within 24 hours, centrifuged plasma will be stored at -20°C or below without freeze-thaw. Sample aliquots will be thawed overnight at between 2-8°C and aliquoted

at room temperature prior to assay performance. We will attempt to measure samples at CISM laboratories. For this, samples will be batched and biochemical biomarkers will be quantitatively measured using an instrumented multi-analyte immunoassay on the Ella platform. suPAR will also be measured using a quantitative near-patient lateral flow assay. Should CISM's laboratory not receive the equipment (ELLA machine) on time to analyse samples locally, samples will be sent to an outside laboratory for centralized analysis.

Should it finally be required to ship samples abroad, samples will be sent to:

Mahidol Oxford Tropical Medicine Research Unit (MORU),

Faculty of Tropical Medicine, Mahidol University,

3/F, 60th Anniversary Chalermprakiat Building, 420/6 Rajvithi Road,

Bangkok 10400 Thailand

Detection of IgM and IgG against SARS-CoV2

We will use the XMAP technology (Luminex) for the detection of IgGs (marker of exposure and immunity) and IgMs (marker of acute infection) against the viral spike (S, including the Receptor Binding Domain RBD in S1), membrane (M), envelope (E) and/or nucleocapsid (N). Antigens (in house produced by Dr. Luis Izquierdo at ISGlobal or available commercially or by collaboration) will be coupled to magnetic microspheres and an immunoassay optimized following in-house methods previously developed and established at ISGlobal laboratories (Figure 2 in Annex) (13; 14). Antigens from *P. falciparum* will be added to the array in order to simultaneously detect active and recent exposure to malaria parasites (17). Plasmas from confirmed positive patients will be used to standardize the immunoassay and plasmas from unexposed individuals will be used to establish the positivity threshold (mean plus three standard deviations).

After genome sequencing of the virus the leftover rRNA or cDNA, serum or plasma samples will be stored up to 20 years for further characterization as currently little is known about this new emerging virus.

*5.*8 Patient follow up for outcome determination in prognostic biomarker component

Participants will be followed-up telephonically at day 7 (window period: +2 days) and day 14 (window period: +4 days) from date of enrolment to determine outcome, with the primary endpoint being development of an oxygen requirement (reported and/or documented peripheral oxygen saturation ≤ 93% or respiratory rate > 30 breaths per minute or clinical indication to give supplemental oxygen). Participants who are admitted at the study site will be followed-up daily during admission to determine whether they progress to meet the primary endpoint.

If telephone contact attempts are unsuccessful for the D14 follow-up, a home visit will be attempted and/or relevant information will be extracted from their medical record if they have sought care since enrolment, or have been transferred elsewhere. If the outreach visit for the D14 follow-up is unsuccessful participants will be declared lost-to-follow-up (LTFU) and further follow-up attempts will not be made. However, if it is established that the reason for inability to contact the participant is that they have been admitted to a health care facility, they will not be declared LTFU until the study team are able to establish contact and determine if the participant has met the primary endpoint or not (i.e. received or required oxygen supplementation).

5.9. Data Collection Forms, Data Entry and Data Analysis

5.9.1. Data collection forms and data entry

Prior to any study procedure, consent will be sought (Appendix A 1, 2, 3, 4 and 5). Clinical information will be extracted from the outpatient (Appendix B) and inpatients (Appendix C) questionnaire for children or specific form for identification of covid-19 suspected cases in children and adults (Appendix D). These forms will contain information on demographics, clinical history and physical exam at admission, underlying conditions, and laboratory results. At hospitals, appendix B and C, which contains clinical data for children, will be collected using paper forms. All data collection forms will be sent to CISM data centre to be double entered into a computerized database in OpenClinica open source software, version 3.0. Copyright© OpenClinica-LLC. The original questionnaire will be stored locally at CISM for

data cleaning. All other clinical data will be collected using standardized questionnaires integrated into ODK program for tablets.

For the three time points of patient contact in the prognostic biomarker component (baseline, D7 and D14), clinical data will be abstracted from clinical records, if available, and outcome data will be verified with the DSS databases. Data will be collected using specific standardized questionnaires. Similarly, specific questionnaires will be developed for COVID-19 population-based serosurveys and all the household members will be asked whether they have had contacts with suspected people during the past 30 days. After this baseline survey, the community members will be asked to phone the Call Centre at CISM's office in Manhiça to notify the study team in case they had a contact with a person that had symptoms of respiratory illness. The Call centre personnel will have a questionnaire to fill in, and advises to give to the participants

CISM laboratory data on SARS-CoV-2 detection will be directly entered in an electronic database where specific data of the study will be extracted for data analysis. An electronic copy containing all study data will be maintained by CISM investigator on a secure computer. Only project personnel will have access to the data, and data available for analysis will be de-identified.

5.8.2. Data Analysis

All statistical analyses will be specified a priori in a formally developed Statistical Analysis Plan.

In brief, the age-specific incidence of severe COVID-19 in the MDH catchment area population will be estimated as the weighted number of cases (weighted by the month specific positivity rate among the collected swabs, see details in the sample size section) over the total number of person-time under surveillance during the study period. Negative binomial regression models will be estimated to identify characteristics associated to COVID-19 positivity and mortality among patients with severe acute respiratory syndrome attending to the hospital. The age-specific seroprevalence of IgM and IgG antibodies against SARS-CoV-2 infection in the general population will be assessed by the proportion of seropositive subjects recruited through the two cross-sectional seroprevalence community surveys. All estimations will be presented with 95% CIs for each age-group and overall.

Mathematical modelling

a) Reproduction number estimation: Estimates of COVID-19 incidence, prevalence and seroprevalence will be used to estimate ongoing transmission and effects of interventions within the local area, with analyses adjusted to the availability of data and stage of the pandemic. Branching process-based estimators of the effective reproduction number will be applied to temporal patterns within the data (particularly number of observed COVID-19 deaths per day). These estimates will be calibrated to data on the timings of interventions and data on movement patterns from sources such as DSS mortality data including pre-COVID period (2015 – 2020) and google community mobility reports (18; 19). Comparing data on observed mortality within the health system and that reported within the community will allow adjustment for under-reporting of deaths and provide insight into national level trends.

b) Refining projections of severity and need for ongoing mitigation measures: Data on patterns of severity by age and according to potential comorbidities such as HIV, malaria and malnutrition, will be integrated into models being developed at Imperial College to provide much more granular model projections of severity at both a national and, regional and continental level (18; 19; 20; 21; 22). In particular, estimates of seroprevalence will be used to understand the denominator population of infections from which severe cases and deaths are being observed (incorporating models of the delay between infections, symptom onset and death which are being increasingly well characterized across a multitude of contexts). This will allow both much more accurate estimation of the stage of the epidemic and the extent to which non-pharmaceutical interventions such as social distancing remain necessary in Mozambique and other settings with similar demographics and where such interventions, if maintained, are highly likely to be causing high economic and societal costs.

5.9. Sample Size Estimation

Sample size for PCR in nasopharyngeal swabs: The objective of the COVID-19 morbidity surveillance is to estimate age specific (<15 and ≥15 years) incidence of severe COVID-19 in

the MDH catchment area population. However, given that a) we do not know how the epidemic curve will evolve and it is difficult to estimate the number of patients with severe acute respiratory syndrome that will attend the hospital each week, and b) we have a limited budget that only covers around 2500 PCRs, we might have to select a sub-sample of nasopharyngeal swabs every month to be analysed by PCR. Should there be months during which the numbers of swabs we collect is larger than the number of PCRs we can conduct, we will estimate the number of severe COVID-19 cases by multiplying the number of patients with severe acute respiratory syndrome by the positivity rate (calculated in those samples that can be analysed by PCR). The positivity rate of the PCR will be calculated by month and age group (<15 and ≥15 years), for which we estimate that a sample size of 200 samples per month and age group will allow to be 95% confident that the positivity rate of SARS-Cov-2 infection by PCR is 10-90% with a maximum admissible error of 4-7% (halfwidth of the confidence interval). Those 400 samples per month will be distributed to ensure we obtain around 100 samples per week (50 per age-group and week). Thus, during weeks during which the number of severe acute respiratory syndrome patients is less than 100, we will analyse all swabs by PCRs, and during weeks where the number is more than 100, we will only analyse the first 100, although the number of unanalysed samples will be counted for the weighted analysis.

<u>Sample size for COVID-19 population-based serosurveys:</u> The sampling will be stratified by age group (<15 and ≥15 years) and will be done by simple random sampling using the demographic surveillance system database as the sampling frame. The age specific group categories might be extended to ***0-19, 20-39, 40-59 and >=60*** if additional co-funding is secured by World Health Organization. For each survey, a ***sample size of 300 individuals*** per age group will be recruited to estimate an age-specific seroprevalence of 10-90% with 3-5% precision and 95% confidence level. ***In the 4th survey will have additionally to the 1200 participants, the number of participants who tested positive in the 3rd survey to do a follow-up visit, totalizing 1900 participants for this 4thserosurvey.*** Randomly selected individuals will be approached and invited to participate in the study. The objectives and procedures of the study will be explained and participants will be included after signing an informed consent. After informed consent a standardized questionnaire will be administrated to participants to

collect socio-demographic information, questions about signs and symptoms suggestive of COVID-19 during the previous 2 months, comorbidities and ongoing medication. A finger-prick sample will also be collected onto filter papers for serological determinations.

Sample size for the study the natural history of SARS-CoV-2 infection**:** A sample size of 43 infected patients and same asymptomatic individuals will allow to estimate the mean time (weeks) to viral load clearance (PCR negativisation) per groups with 95% confidence while the marginal error in estimate does not exceed 2 days (0.30 weeks) assuming the range of PCR-positivity in the population is 2-6 weeks.

Assuming a loss to follow up of 15%, we will recruit 50 patients in each of the two groups (symptomatic and asymptomatic individuals).

6. CAPACITY BUILDING

The present project will strengthen the diagnostics capacities at CISM for the detection of SARS-CoV-2 RNA, the use of XMAP technology for the quantification of antibodies against the virus, and for the interpretation of genomic data. The capacity building process will start with a start-up stage to train CISM personnel on the study specific procedures and requirements. In addition, training will be performed on a regular basis to ensure that Good Clinical Practices (GCP) guidelines are followed and that study procedures are correctly implemented. In addition, laboratory training for COVID 19 PCR testing will be provided to the staff by the National Institute of Health (Instituto Nacional de Saúde – INS) of Mozambique. This will include the inter-laboratory comparison to ensure the quality control.

7. RESULTS SHARING AND DISSEMINATION

Results for immediate action will be shared with the Ministry of Health through COVID-19 data reporting platform. The participants will be notified for the results according to procedures established by the Ministry of Health for COVID-19 ensuring that infected individuals are followed accordingly as established at the District health authorities. Residency identification will be collected to facilitate the location of positive cases for proper follow up.

Results of this study will be presented to and discussed with public-health officials, policymakers, health professionals within the Ministry of Health in Mozambique, international public health organization, partners and other relevant institutions, at scientific meetings, presentations and publications. The Instituto Nacional de Saúde, will be coordinating the regular data meetings for data sharing with the Ministry of Health and other key local stakeholders

Results will also be disseminated through scientific meetings in Mozambique and outside country. Manuscripts of the results finding, will be written within 12 months of study finalization, led by local authors and respecting the definition for authorship as described by the International Committee of Medical Journal Editors (ICMJE).

8. PROTECTION OF HUMAN SUBJECTS

The protocol, consent form and data collection tools for this surveillance will be reviewed and approved by the CISM Institutional Bioethic Committee for Health (CIBS-CISM) and Mozambican Bioethic committee for health (CNBS).

The HDSS data collection has ethical approval from CIBS-CISM and the CNBS, (see annexes 2 and 3). In addition, all the heads of households and household members in Manhiça district have voluntarily agreed and signed a written detailed informed consent for providing their demographic and socio-economic data, including that of their households and their young dependants (children under de age of 18 years), in the context of HDSS. To protect confidentiality, all the data that may lead to the identification of the study participant or their relative will not be used during the publication or presentation of the results of this study.

A. All documents and databases will be maintained in locked cabinets and on a password accessible computer, to limit access to authorized study personnel.

B. Risk and strategies to minimize risks

We are aware of the existence of several studies underway at the MDH. However, due to the relevance of the study to the control of the COVID-19 epidemic in the country and the unique conditions existing in this hospital that we mentioned above, we believe it is the ideal place to carry out the study. So, if an individual is participating in other studies that are not clinical trials, we will offer informed consent to participate in this study. To reduce unnecessary discomfort to participants, we will share samples with ongoing studies.

There are no physical risks to the participants involved beyond normal clinical care at hospital. However, participants and/or parents or guardian of participants may feel embarrassed or uncomfortable over interview when discuss sensitive topics, such as past medical or vaccination history, etc.

- Questions and physical exam will be conducted in private areas.
- All documents and databases will be maintained in locked cabinets and on a password-accessible computer, to limit access to authorized study personnel.
- The evaluation presents minimal risk to the participants especially over blood and swab collection, which would cause light pain in the nose or site of blood collection.
- Enrollment of children or adults in the study will not result in a loss of privacy or confidentiality for the family. All health problems identified in the participants will be management properly according to the Good Clinical Practices.

C. Biosecurity procedures

Prevention of COVID-19 infection in investigation personnel: Samples will be collected following biosafety guidelines such as the use of masks and protective clothes. All personnel involved in the investigation will be trained in procedures for infection prevention and control. Samples will be transported in cool boxes from the point of collection to the testing laboratory. Shipment of samples to external laboratories for whole genome sequencing will follow standard recommendations (23).

All biological samples for detection of COVID-19 will be tested at CISM following international biosecurity recommendations (24; 25). CISM is equipped with Biosafety Level II Laboratory suitable for the diagnosis of COVID-19. All technical staff will use personal protective equipment. Furthermore, all procedures suspect to generate aerosols will be carried out in a validated class II Biosafety cabinet. Infectious waste material will be autoclaved following incineration according to the waste management procedure.

D. Benefits

The individual participant will not receive any direct personal benefit from participating in this research. However, the results of this study has the potential to produce an immediate beneficial public health impact at both regional and global level improving disease

management, prevention and control of COVID-19 outbreak in Mozambique, and other subSaharan African countries, by providing evidence-based information for translation into policy.

9. BUDGET

The estimated budget for the proposed project is $566,654.

ACTIVITES	YEAR 1	YAER 2	TOTAL
Personnel	$127,288.24	$27,357.38	$154,646
Consumables	$132,496.77	$126,749.56	$259,246
Equipment	$9,583.53	$0.00	$9,584
Travel	$0.00	$0.00	$0.00
Other Direct Costs	$30,261.02	$18,474.92	$48,736
**Subtotal Direct Cost**	**$299,629.56**	**$172,581.86**	**$472,211.42**
**Indirect Cost**	**$59,925.91**	**$34,516.37**	**$94,442.28**
**Total Budget**	**$359,555**	**$207,098**	**$566,654**

10. TIMELINE

	Key Activities	Year 1						Year 2											
		J	A	S	O	N	D	J	F	M	A	M	J	J	A	S	O	N	D
1	IRB Submission CCI																		
2	IRB Submission and approval CIBS																		
3	IRB Submission and approval CNBS																		
4	Administrative approval																		
5	Stand forms and databases																		
6	Staff identification																		
7	Training staff																		
8	Begin of study implementation																		
	Study specific activities																		
9	COVID-19 morbidity surveillance																		
10	COVID-19 mortality surveillance																		
11	COVID-19 population-based serosurveys																		
12	Longitudinal cohort of SARS-CoV-2 acutely infected symptomatic individuals																		
13	Sample processing at CISM laboratory																		
14	Data management and data entry regular cleaning																		
15	Result dissemination to share with MOH/EPI																		
16	Shipment of specimens for SARS-CoV-2 sequencing to ISGlobal																		
17	Result dissemination to share with MOH/EPI																		
18	Finally analysis																		
19	Dissemination results and publications																		

11. REFERENCES

1. **Team, I.C.C.-R.,.** *Report 12: The Global Impact of COVID-19 and Strategies for Mitigation and Suppression.* s.l. : https://www.imperial.ac.uk/mrc-global-infectious-disease-analysis/covid-19/report-12global-impact-covid19/.
2. **WHO.** *Coronavirus disease (COVID-2019) situation reports.* . s.l. : https://www.who.int/emergencies/diseases/novel-coronavirus-2019/situation-reports, , 2020.
3. **Li, Q., et al.,.** *Early Transmission Dynamics in Wuhan, China, of Novel Coronavirus-Infected Pneumonia.* . s.l. : N Engl J Med, 2020. 382(13): p. 1199-1207.
4. **Cowling BJ, Leung GM.** *Epidemiological research priorities for public health control of the ongoing global novel coronavirus (2019-nCoV) outbreak* . s.l. : Euro Surveill. 2020;25(6).
5. **Wu P, Hao X, Lau EHY, Wong JY, Leung KSM, Wu JT, et al.** *Real-time tentative assessment of the epidemiological characteristics of novel coronavirus infections in Wuhan, China, as at 22 January 2020.* s.l. : Euro Surveill. 2020;25(3).
6. **Wang Y, Liu Y, Liu L, Wang X, Luo N, Ling L.** *Clinical outcome of 55 asymptomatic cases at the time of hospital admission infected with SARS-Coronavirus-2 in Shenzhen, China.* s.l. : J Infect Dis. 2020.
7. **Wilson N, Kvalsvig A, Barnard L, Baker M.** *Estimating the Case Fatality Risk of COVID-19 using Cases from Outside* . s.l. : medRxiv. 2020.
8. **Lescure FX, Bouadma L, Nguyen D, Parisey M, Wicky PH, Behillil S, et al.** *Clinical and virological data of the first cases of COVID-19 in Europe: a case series.* s.l. : Lancet Infect Dis. 2020.
9. **Nhantumbo AA, Weldegebriel G, Katsande R, de Gouveia L, Comé CE, Cuco AZ, et al.** *Surveillance of impact of PCV-10 vaccine on pneumococcal meningitis in Mozambique, 2013 - 2015* . s.l. : PLoS One. 2017 Jun 12;12(6):e0177746. doi: 10.1371/journal.pone.0177746. eCol.
10. **Sigaúque B, Roca A, Mandomando I, Morais L, Quintó L, Sacarlal J, et al.** *Community-acquired bacteremia among children admitted to a rural hospital in Mozambique* . s.l. : Pediatr Infect Dis J. 2009 Feb;28(2):108–13.
11. **MISAU.** *http://www.misau.gov.mz/index.php/informacao-sobre-coronavirus-covid-19.*
12. **WHO.** *https://apps.who.int/iris/bitstream/handle/10665/331231/WHO-2019-nCoVSurveillanceGuidance-2020.4-por.pdf?sequence=33&isAllowed=y.*
13. **Fonseca AM, Gonzalez R, Bardaji A, Jairoce C, Ruperez M, Jimenez A, et al.** *VAR2CSA Serology to Detect Plasmodium falciparum Transmission Patterns in Pregnancy* . s.l. : Emerg Infect Dis. 2019;25(10):1851-60.
14. **Fonseca AM, Quinto L, Jimenez A, Gonzalez R, Bardaji A, Maculuve S, et al.** *Multiplexing detection of IgG against Plasmodium falciparum pregnancy-specific antigens.* s.l. : PLoS One. 2017;12(7):e0181150.
15. **Salzberg NT, Sivalogan K, Bassat Q, Taylor AW, Adedini S, et al.** *Mortality Surveillance Methods to Identify and Characterize Deaths in Child Health and Mortality Prevention Surveillance Network Sites.* s.l. : Clin Infect Dis. 2019 Oct 9;69(Supplement_4):S262-S273. doi: 10.1093/cid/ciz599.

16. **Rakislova N, Fernandes F, Lovane L, et al.** *Standardization of minimally invasive tissue sampling specimen collection and pathology training for the Child Health and Mortality Prevention Surveillance Network.* s.l. : Clin Infect Dis 2019; 69(Suppl 4):S302–10.

17. **Martianez-Vendrell X, Jimenez A, Vasquez A, Campillo A, Incardona S, Gonzalez R, et al.** *Quantification of malaria antigens PfHRP2 and pLDH by quantitative suspension array technology in whole blood, dried blood spot and plasma.* s.l. : Malar J. 2020;19(1):12.

18. **Team, Imperial College COVID-19 Response.** *Report 13 - Estimating the number of infections and the impact of non-pharmaceutical interventions on COVID-19 in 11 European countries .*

19. ***Google.*** *Mobility changes Mozambique.* s.l. : https://wwwgstaticcom/covid19/mobility/20200405_MZ_Mobility_Report_enpdf. 2020.

20. **Davies N, Klepac P, Liu Y, Prem K, Jit M, group CC-w, et al.** *Age-dependent effects in the transmission and control of COVID-19 epidemics.* . s.l. : medRxiv. 2020.

21. **Verity R, Okell LC, Dorigatti I, Winskill P, Whittaker C, Imai N, et al.** *Estimates of the severity of coronavirus disease 2019: a model-based analysis* . s.l. : Lancet Infect Dis. 2020.

22. T**eam, IHME COVID-19 health service utilization forecasting.** *Forecasting COVID-19 impact on hospital bed-days, ICU-days, ventilator-days and deaths by US state in the next 4 months.* s.l. : medRxiv. 2020.

23. **WHO.** *Guidance for laboratories shipping specimens to WHO reference laboratories that provide confirmatory testing for COVID-19 virus: interim guidance, 31 March 2020.* . s.l. : https://apps.who.int/iris/handle/10665/331639. 2020.

24. **WHO.** *Laboratory biosafety guidance related to coronavirus disease (COVID-19): interim guidance, 19 March 2020.* . s.l. : World Health Organization. https://appswhoint/iris/handle/10665/331500 2020.

25. **WHO.** *Laboratory testing for coronavirus disease (COVID-19) in suspected human cases: interim guidance, 19 March 2020.* s.l. : https://apps.who.int/iris/handle/10665/331501. 2020.

12. APPENDICES

Appendix A.1: Informed consent for parent/guardian of children

Appendix A.2: Informed consent for adult

Appendix A.3: Assent for children 12-17 years of age

Appendix B: Inpatient form for children (INPD4)

Appendix C: Outpatient form for children (OPD5)

Appendix D: Form for identification of covid-19 suspected cases

Appendix E: Questionnaire for community survey

ORIGINAL

CÓPIA

Vigilância Hospitalar e Pesquisa de Contactos	**Estudo:** COVID-19 Surveillance in Rural Mozambique for a prompt and effective response (MozCOVID)	
cism centro de investigação em saúde de manhiça	**Formulário para identificação de caso suspeito de COVID-19**<br>PEE_MozCOVID_001_A04_v02_PT	

DADOS GERAIS

1. Data de Inquérito (dd/mm/aaaa) |___|___|/|___|___|/|___|___|___|___|
2. Unidade Sanitária
   - ☐ Hospital Distrital da Manhiça
   - ☐ Centro de Saúde de Maragra
   - ☐ Hospital Rural de Xinavane
   - ☐ Centro de Saúde de Palmeiras
   - ☐ Centro de Saúde de Ilha Josina
   - ☐ Outro, Especifique ______________________________
3. Se amostra foi colhida na comunidade, especifique o Bairro/Povoação

   ____

DADOS DO UTENTE/PACIENTE

4. Número do estudo do participante |___|___|___|___|-|___|___|

5. Nome do utente/paciente
   |___|___|___|___|___|___|___|___|___|___|___|___|___|___|___|___|___|___|___|___|___|
6. N° de identificação, *perm_id* (se aplicável) |___|___|___|___|-|___|___|___|-|___|___|
7. N° de OPD, se aplicável ☐ N/A |___|___|___|___|___|___|___|
8. Nº de INPD, se aplicável ☐ N/A |___|___|___|___|___|___|___|___|
9. Data de nascimento (dd/mm/aaaa) |___|___|/|___|___|/|___|___|___|___|
   a Se adulto idade (em anos) |___|___|

10. Sexo ☐ Masculino ☐ Feminino
11. Se mulher, está grávida? ☐ Sim ☐ Não
12. Bairro/Povoação/Acampamento |___|___|___|___|___|___|___|___|___|___|___|___|___|
    11.1. Ponto de referência
    |___|___|___|___|___|___|___|___|___|___|___|
13. Telefone:
    12.1. |___|___|___|___|___|___|___|___|___| 12.2.|___|___|___|___|___|___|___|___|___|

INFORMAÇÃO CLÍNICA

14. Peso: |___|___|___|. |___| (Kg)
15. Altura: |___|___|___|. |___| (cm)
16. Temperatura: |___|___|. |___| (°c)
17. Frequência respiratória: |___|___|___| (ciclos por minuto)
18. Saturação O2: |___|___| (%)

Sintomas primários

19. Febre ☐ Sim ☐ Não N° de dias |___|___|

ORIGINAL

CÓPIA

20. Tosse seca	☐ Sim	☐ Não		Nº de dias	|__|__|
21. Tosse produtiva	☐ Sim	☐ Não		Nº de dias	|__|__|
22. Dificuldade respiratória/falta de ar	☐ Sim	☐ Não		Nº de dias	|__|__|

Sintomas adicionais

23. Dores musculares	☐ Sim	☐ Não	☐N/A	Nº de dias	|__|__|
24. Dores articulares	☐ Sim	☐ Não	☐N/A	Nº de dias	|__|__|
25. Dores de garganta	☐ Sim	☐ Não	☐N/A	Nº de dias	|__|__|
26. Dores de cabeça	☐ Sim	☐ Não	☐N/A	Nº de dias	|__|__|
27. Coriza/rinorreia	☐ Sim	☐ Não	☐N/A	Nº de dias	|__|__|
28. Fraqueza geral	☐ Sim	☐ Não	☐N/A	Nº de dias	|__|__|
29. Perda de olfato ou paladar	☐ Sim	☐ Não	☐N/A	Nº de dias	|__|__|
30. Náuseas ou vómitos	☐ Sim	☐ Não	☐N/A	Nº de dias	|__|__|
31. Diarreias	☐ Sim	☐ Não	☐N/A	Nº de dias	|__|__|

32. Outros, especificar: ______________________

HISTÓRICO CLÍNICO DE COVID-19

33. Já foi diagnosticado de COVID?	☐ Sim ☐ Não
34. Se sim, data de diagnóstico?	|__|__|/|__|__|/|__|__|__|__|
35. Já foi internado num hospital ou centro de saúde?	☐ Sim ☐ Não
36. Se sim, data de internamento?	|__|__|/|__|__|/|__|__|__|__|
37. Quantos dias de internamento?	|__|__|
38. Já foi diagnosticado sem ter tido sintomas?	☐ Sim ☐ Não
39. Se sim, data do diagnóstico?	|__|__|/|__|__|/|__|__|__|__|
40. Já foi vacinado contra COVID-19?	☐ Sim ☐ Não ☐N/A
41. Se sim, data de vacinação?	|__|__|/|__|__|/|__|__|__|__|
42. Teve alguma reacção adversa?	☐ Sim ☐ Não
43. Se sim, especifique	

____

FACTORES DE RISCO

44. Esteve em contacto com um caso confirmado por laboratório.
☐ Sim ☐ Não ☐ Desconhece

45. Esteve em contacto com casos considerados suspeitos ou prováveis? *Não confirmado por laboratório*
☐ Sim ☐ Não ☐ Desconhece

46. Esteve em um país com casos confirmados?
☐ Sim ☐ Não ☐ Desconhece
46.1. Se sim, indique o país?
☐ África do Sul
☐ Eswatini
☐ Portugal
☐ Outro, especificar: ______________

47. Esteve em contacto com indivíduos provenientes de países com casos confirmados?
☐ Sim ☐ Não ☐ Desconhece
47.1. Se sim, indique o país?
☐ África do Sul

ORIGINAL

☐ Eswatini
☐ Portugal
☐ Outro, especificar: ____________

48. Participou em um evento ou esteve um local com mais de 10 pessoas nos últimos 14 dias?
☐ Sim ☐ Não ☐ Desconhece
48.1 Se sim, especifica:
☐ Mercado
☐ Igreja
☐ Cerimonias fúnebres
☐ Meios de transporte (chapas ou machimbombo)
☐ Hospitais/centros de saúde
☐ Outro, especificar: ____________________

49. Esteve em contacto com um doente com infecção respiratória nos últimos 14 dias?
☐ Sim ☐ Não ☐ Desconhece
49.1 Se sim, indique a provável patologia?
☐ Infecção respiratória não complicada, sem internamento hospitalar
☐ Infecção respiratória que foi necessário internamento hospitalar
☐ Tuberculose
☐ Outra, especificar: ______________________

50. Participação em cerimónias fúnebres do falecido com infecção respiratória
☐ Sim ☐ Não ☐ Desconhece

51. Tem doenças crónicas conhecidas:
☐ HIV-SIDA
☐ Tuberculose
☐ Hipertensão
☐ Diabetes mellitus
☐ Asma
☐ Outra, especificar: ________________

52. Tem critérios de testagem laboratorial? ☐ Sim ☐ Não

Devem ser testados todos casos suspeitos e casos prováveis para COVID-19, segundo as definições abaixo.

Considerar **caso suspeito** para COVID-19, se apresentar sintomas respiratórios &

- História de contacto com casos confirmados; **OU**
- História de contacto com casos suspeitos ou prováveis; **OU**
- Em indivíduos que estiveram em países com casos confirmados; **OU**
- Em indivíduos que estiveram em contacto com pessoas provenientes de países com casos confirmados; **OU**
- Sem outra etiologia que justifique os sintomas; **OU**

*Considerar **caso provável** para COVID-19, ao caso suspeito de COVID19 em que o teste não pode ser realizado por algum motivo ou cujo resultado tenha sido inconclusivo

53. Colheu amostra respiratória? ☐ Sim ☐ Não
a Se sim, data: |___|___|/|___|___|/|___|___|___|___|

ORIGINAL

CÓPIA

b Tipo de amostras:
- ☐ Zaragatoa nasofaríngea
- ☐ Zaragatoa orofaríngea
- ☐ Outra, especificar: ____________________

54. NIDA____________________

55. Foram colhidas outras amostras? ☐ Sim ☐ Não
    - a Se sim, data: |__|__|/|__|__|/|__|__|__|__|
    - b Especificar tipo de amostras____________________________

56. Foi feito teste de malária? ☐ Sim ☐ Não
    - a Se sim, tipo de teste: ☐ TDR-malaria ☐microscopia
    - b Resultado: ☐ Positivo ☐ Negativo ☐ Indeterminado

Se não for contacto de um caso positivo, responder abaixo:

57. Diagnóstico na triagem:
    - a ____
    - b ____
    - c ____
    - d ____

58. Destino após a triagem:
    - ☐ Banco de socorro ou ICD
    - ☐ Alta
    - ☐ Abandono

59. Diagnóstico no ICD ou Banco de socorro:
    - a ____
    - b ____
    - c ____

60. Destino após ICD ou banco de socorro:
    - ☐ Internamento
    - ☐ Transferência
    - ☐ Óbito
    - ☐ Alta
    - ☐ Abandono

61. Código, assinatura e data: |__|__|__|__| ________________ |__|__|/|__|__|/|__|__|__|__|

REVISÃO FINAL DO FORMULÁRIO

62. Código, Assinatura e data: |__|__|__|__| ________________ |__|__|/|__|__|/|__|__|__|__|

List of positive SARS-CoV-2 samples from Mozambique sequenced in this study. The variant each sequence belongs to is detailed.

Variant	ID	Reference Center	Hospital date (yyyy/mm/dd)	Sample type	Ct/Cq PCR ORF1ab	Ct/Cq PCR gen E	Ct/Cq PCR gen N	Ct/Cq PCR gen S	Ct/Cq PCR RdRP	PCR concentration (ng/ul)	Coverage	Median depth	Sequencing technology	Sequencing library	GISAID ID	GISAID EPI	ENA accession	Gender	Age	Residence city	Residence province	Residence country	Lineage	Mutations	Percent SarsCov2	COVID 19 Symptoms	Comorbidities
Beta	COV025276	Instituto de Salud Global - ISGLOBAL (CAT_ISG)	2021-02-01	RNA		19.7	4		21.07	65.5	0.9908	2365	Illumina MiSeq	ARTICV3 + Nextera Flex	hCoV-19/Mozambique/MZ-L-IBV-97025276/2021	EPI_ISL_2442084	ERR6018062	Female	31-45	Manhiça	Maputo	Mozambique	B.1.351	E484K_S(1.0)&N501Y_S(1.0)&D614G_S(1.0)&A701V_S(1.0)&K417N_S(0.91)	98.82	NA	NA
Beta	COV025278	Instituto de Salud Global - ISGLOBAL (CAT_ISG)	2021-03-03	RNA		30.38	20		31.47	4.3	0.9392	939	Illumina MiSeq	ARTICV3 + Nextera Flex	hCoV-19/Mozambique/MZ-L-IBV-97025278/2021	EPI_ISL_2442085	ERR6018063	Female	46-60	Manhiça	Maputo	Mozambique	B.1.351	E484K_S(1.0)&N501Y_S(1.0)&D614G_S(1.0)&A701V_S(1.0)&K417N_S(0.96)	80.01	NA	NA
Beta	COV025279	Instituto de Salud Global - ISGLOBAL (CAT_ISG)	2021-03-04	RNA		32.57	23		33.03	2.9	0.9338	642	Illumina MiSeq	ARTICV3 + Nextera Flex	hCoV-19/Mozambique/MZ-L-IBV-97025279/2021	EPI_ISL_3246530	ERR6018064	Male	15-30	Manhiça	Maputo	Mozambique	B.1.351	E484K_S(1.0)&N501Y_S(1.0)&D614G_S(1.0)&T716I_S(0.15)&A701V_S(1.0)&K417N_S(0.84)	50.35	NA	NA
Beta	COV025280	Instituto de Salud Global - ISGLOBAL (CAT_ISG)	2021-03-03	RNA		31.32	21		33.15	2.6	0.9666	1058	Illumina MiSeq	ARTICV3 + Nextera Flex	hCoV-19/Mozambique/MZ-L-IBV-97025280/2021	EPI_ISL_2442086	ERR6018065	Male	31-45	Manhiça	Maputo	Mozambique	B.1.351	E484K_S(1.0)&N501Y_S(1.0)&D614G_S(1.0)&A701V_S(1.0)&K417N_S(0.98)	68.43	NA	NA
Beta	COV025281	Instituto de Salud Global - ISGLOBAL (CAT_ISG)	2021-03-03	RNA		21.4	10		22.56	78.5	0.99	1123	Illumina MiSeq	ARTICV3 + Nextera Flex	hCoV-19/Mozambique/MZ-L-IBV-97025281/2021	EPI_ISL_2442087	ERR6018066	Female	46-60	Manhiça	Maputo	Mozambique	B.1.351	E484K_S(1.0)&N501Y_S(1.0)&D614G_S(1.0)&A701V_S(1.0)&K417N_S(0.91)	98.63	NA	NA
Beta	COV025285	Instituto de Salud Global - ISGLOBAL (CAT_ISG)	2021-02-01	RNA		25.11	7		26.25	29.7	0.9537	1014	Illumina MiSeq	ARTICV3 + Nextera Flex	hCoV-19/Mozambique/MZ-L-IBV-97025285/2021	EPI_ISL_2442088	ERR6018067	Female	46-60	Manhiça	Maputo	Mozambique	B.1.351	L18F_S(1.0)&E484K_S(1.0)&N501Y_S(1.0)&D614G_S(1.0)&A701V_S(1.0)&K417N_S(0.91)	96.59	NA	NA
Beta	COV025290	Instituto de Salud Global - ISGLOBAL (CAT_ISG)	2021-02-01	RNA			9			10.3	0.9375	1081	Illumina MiSeq	ARTICV3 + Nextera Flex	hCoV-19/Mozambique/MZ-L-IBV-97025290/2021	EPI_ISL_2442089	ERR6018068	Male	31-45	Manhiça	Maputo	Mozambique	B.1.351	E484K_S(1.0)&N501Y_S(1.0)&D614G_S(1.0)&A701V_S(1.0)&K417N_S(0.92)	91.81	NA	NA
Beta	COV025296	Instituto de Salud Global - ISGLOBAL (CAT_ISG)	2021-02-20	RNA			5			165	0.9901	1262	Illumina MiSeq	ARTICV3 + Nextera Flex	hCoV-19/Mozambique/MZ-L-IBV-97025296/2021	EPI_ISL_2442090	ERR6018073	Female	46-60	Manhiça	Maputo	Mozambique	B.1.351	E484K_S(1.0)&N501Y_S(1.0)&D614G_S(1.0)&A701V_S(1.0)&K417N_S(0.86)	98.85	NA	NA
Beta	COV025299	Instituto de Salud Global - ISGLOBAL (CAT_ISG)	2021-03-04	RNA			7			100.5	0.985	1193	Illumina MiSeq	ARTICV3 + Nextera Flex	hCoV-19/Mozambique/MZ-L-IBV-97025299/2021	EPI_ISL_2442091	ERR6018075	Male	>60	Manhiça	Maputo	Mozambique	B.1.351	E484K_S(1.0)&N501Y_S(1.0)&D614G_S(1.0)&A701V_S(1.0)&K417N_S(0.91)	98.51	NA	NA
Beta	COV025302	Instituto de Salud Global - ISGLOBAL (CAT_ISG)	2021-02-20	RNA			20			2.7	0.9135	726	Illumina MiSeq	ARTICV3 + Nextera Flex	hCoV-19/Mozambique/MZ-L-IBV-97025302/2021	EPI_ISL_2442092	ERR6018076	Female	15-30	Manhiça	Maputo	Mozambique	B.1.351	E484K_S(1.0)&N501Y_S(1.0)&D614G_S(1.0)&A701V_S(1.0)&K417N_S(0.91)&V367F_S(0.21)	79.2	NA	NA
Beta	COV025303	Instituto de Salud Global - ISGLOBAL (CAT_ISG)	2021-02-17	RNA			23			5.3	0.9057	374	Illumina MiSeq	ARTICV3 + Nextera Flex	hCoV-19/Mozambique/MZ-L-IBV-97025303/2021	EPI_ISL_2442093	ERR6018077	Male	15-30	Manhiça	Maputo	Mozambique	B.1.351	L18F_S(0.83)&E484K_S(1.0)&N501Y_S(1.0)&D614G_S(1.0)&A701V_S(1.0)&K417N_S(0.81)	39.53	NA	NA
Beta	COV025304	Instituto de Salud Global - ISGLOBAL (CAT_ISG)	2021-02-19	RNA			20			3	0.9115	688	Illumina MiSeq	ARTICV3 + Nextera Flex	hCoV-19/Mozambique/MZ-L-IBV-97025304/2021	EPI_ISL_2442094	ERR6018078	Male	>60	Manhiça	Maputo	Mozambique	B.1.351	E484K_S(1.0)&N501Y_S(1.0)&D614G_S(1.0)&A701V_S(1.0)&K417N_S(0.93)	69.55	NA	NA
Beta	COV025305	Instituto de Salud Global - ISGLOBAL (CAT_ISG)	2021-02-17	RNA			17			24.4	0.9617	1149	Illumina MiSeq	ARTICV3 + Nextera Flex	hCoV-19/Mozambique/MZ-L-IBV-97025305/2021	EPI_ISL_2442095	ERR6018079	Female	15-30	Manhiça	Maputo	Mozambique	B.1.351	E484K_S(1.0)&N501Y_S(1.0)&D614G_S(1.0)&A701V_S(1.0)&K417N_S(0.93)	93.67	NA	NA
Beta	COV025308	Instituto de Salud Global - ISGLOBAL (CAT_ISG)	2021-02-12	RNA			11			104	0.9891	1341	Illumina MiSeq	ARTICV3 + Nextera Flex	hCoV-19/Mozambique/MZ-L-IBV-97025308/2021	EPI_ISL_2442096	ERR6018080	Male	31-45	Manhiça	Maputo	Mozambique	B.1.351	E484K_S(1.0)&N501Y_S(1.0)&D614G_S(1.0)&A701V_S(1.0)&K417N_S(0.88)	98.46	NA	NA
Beta	COV025309	Instituto de Salud Global - ISGLOBAL (CAT_ISG)	2021-02-17	RNA			10			81.5	0.9898	1321	Illumina MiSeq	ARTICV3 + Nextera Flex	hCoV-19/Mozambique/MZ-L-IBV-97025309/2021	EPI_ISL_2442097	ERR6018081	Female	31-45	Manhiça	Maputo	Mozambique	B.1.351	E484K_S(1.0)&N501Y_S(1.0)&D614G_S(1.0)&A701V_S(1.0)&K417N_S(0.88)	98.69	NA	NA
Beta	COV025310	Instituto de Salud Global - ISGLOBAL (CAT_ISG)	2021-02-17	RNA			15			21.9	0.9841	1283	Illumina MiSeq	ARTICV3 + Nextera Flex	hCoV-19/Mozambique/MZ-L-IBV-97025310/2021	EPI_ISL_2442098	ERR6018086	Female	31-45	Manhiça	Maputo	Mozambique	B.1.351	E484K_S(1.0)&N501Y_S(1.0)&D614G_S(1.0)&A701V_S(1.0)&K417N_S(0.94)	97.08	NA	NA
Beta	COV025311	Instituto de Salud Global - ISGLOBAL (CAT_ISG)	2021-02-12	RNA			17			45	0.9834	1357	Illumina MiSeq	ARTICV3 + Nextera Flex	hCoV-19/Mozambique/MZ-L-IBV-97025311/2021	EPI_ISL_2442099	ERR6018088	Male	31-45	Manhiça	Maputo	Mozambique	B.1.351	E484K_S(1.0)&N501Y_S(1.0)&D614G_S(1.0)&A701V_S(1.0)&K417N_S(0.93)	97.75	NA	NA
Beta	COV025315	Instituto de Salud Global - ISGLOBAL (CAT_ISG)	2021-02-12	RNA			20			13.4	0.9629	1250	Illumina MiSeq	ARTICV3 + Nextera Flex	hCoV-19/Mozambique/MZ-L-IBV-97025315/2021	EPI_ISL_2442100	ERR6018089	Female	15-30	Manhiça	Maputo	Mozambique	B.1.351	L18F_S(0.94)&E484K_S(1.0)&N501Y_S(1.0)&D614G_S(1.0)&A701V_S(1.0)&K417N_S(0.97)	94.57	NA	NA
Beta	COV025321	Instituto de Salud Global - ISGLOBAL (CAT_ISG)	2021-02-12	RNA			18			3.8	0.9232	748	Illumina MiSeq	ARTICV3 + Nextera Flex	hCoV-19/Mozambique/MZ-L-IBV-97025321/2021	EPI_ISL_2442101	ERR6018090	Female	15-30	Manhiça	Maputo	Mozambique	B.1.351	L18F_S(0.5)&E484K_S(1.0)&N501Y_S(1.0)&D614G_S(1.0)&A701V_S(1.0)&K417N_S(0.96)	66.87	NA	NA
Beta	COV025326	Instituto de Salud Global - ISGLOBAL (CAT_ISG)	2021-02-18	RNA			10			76.5	0.985	1282	Illumina MiSeq	ARTICV3 + Nextera Flex	hCoV-19/Mozambique/MZ-L-IBV-97025326/2021	EPI_ISL_2442103	ERR6018092	Female	<15	Manhiça	Maputo	Mozambique	B.1.351	L18F_S(1.0)&E484K_S(1.0)&N501Y_S(1.0)&D614G_S(1.0)&A701V_S(1.0)&K417N_S(0.92)	98.55	NA	NA
Beta	COV025327	Instituto de Salud Global - ISGLOBAL (CAT_ISG)	2021-02-24	RNA			11			59.5	0.9853	1305	Illumina MiSeq	ARTICV3 + Nextera Flex	hCoV-19/Mozambique/MZ-L-IBV-97025327/2021	EPI_ISL_2442104	ERR6018093	Male	31-45	Manhiça	Maputo	Mozambique	B.1.351	E484K_S(1.0)&N501Y_S(1.0)&D614G_S(1.0)&A701V_S(1.0)&K417N_S(0.91)	98.28	NA	NA
Beta	COV025340	Instituto de Salud Global - ISGLOBAL (CAT_ISG)	2021-02-01	RNA			15			9.46	0.9451	1078	Illumina MiSeq	ARTICV3 + Nextera Flex	hCoV-19/Mozambique/MZ-L-IBV-97025340/2021	EPI_ISL_2442106	ERR6018095	Male	31-45	Manhiça	Maputo	Mozambique	B.1.351	L18F_S(0.8)&E484K_S(1.0)&N501Y_S(1.0)&D614G_S(1.0)&A701V_S(1.0)&G1219V_S(0.07)&K417N_S(0.97)	87.78	NA	NA
Beta	COV025341	Instituto de Salud Global - ISGLOBAL (CAT_ISG)	2021-02-09	RNA			18			2.55	0.916	821	Illumina MiSeq	ARTICV3 + Nextera Flex	hCoV-19/Mozambique/MZ-L-IBV-97025341/2021	EPI_ISL_3246522	ERR6018097	Male	15-30	Manhiça	Maputo	Mozambique	B.1.351	L18F_S(0.27)&E484K_S(1.0)&N501Y_S(1.0)&D614G_S(1.0)&A701V_S(1.0)&K417N_S(0.95)	68.28	NA	NA
Beta	COV025342	Instituto de Salud Global - ISGLOBAL (CAT_ISG)	2021-02-09	RNA			10			88	0.9896	1489	Illumina MiSeq	ARTICV3 + Nextera Flex	hCoV-19/Mozambique/MZ-L-IBV-97025342/2021	EPI_ISL_2442107	ERR6018098	Male	31-45	Manhiça	Maputo	Mozambique	B.1.351	E484K_S(1.0)&N501Y_S(1.0)&D614G_S(1.0)&A701V_S(1.0)&K417N_S(0.92)	98.32	NA	NA
Beta	COV025343	Instituto de Salud Global - ISGLOBAL (CAT_ISG)	2021-02-02	RNA			3			142.5	0.9902	1254	Illumina MiSeq	ARTICV3 + Nextera Flex	hCoV-19/Mozambique/MZ-L-IBV-97025343/2021	EPI_ISL_2442108	ERR6018099	Male	46-60	Manhiça	Maputo	Mozambique	B.1.351	E484K_S(1.0)&N501Y_S(1.0)&D614G_S(1.0)&A701V_S(1.0)&K417N_S(0.84)	98.86	NA	NA
Beta	COV025345	Instituto de Salud Global - ISGLOBAL (CAT_ISG)	2021-02-09	RNA			3			134	0.9904	1446	Illumina MiSeq	ARTICV3 + Nextera Flex	hCoV-19/Mozambique/MZ-L-IBV-97025345/2021	EPI_ISL_2442109	ERR6018100	Male	31-45	Manhiça	Maputo	Mozambique	B.1.351	L18F_S(1.0)&E484K_S(1.0)&N501Y_S(1.0)&D614G_S(1.0)&A701V_S(1.0)&K417N_S(0.86)	98.88	NA	NA
Beta	COV025346	Instituto de Salud Global - ISGLOBAL (CAT_ISG)	2021-02-01	RNA			2			60	0.9888	1466	Illumina MiSeq	ARTICV3 + Nextera Flex	hCoV-19/Mozambique/MZ-L-IBV-97025346/2021	EPI_ISL_2442110	ERR6018101	Male	15-30	Manhiça	Maputo	Mozambique	B.1.351	L18F_S(1.0)&E484K_S(1.0)&N501Y_S(1.0)&D614G_S(1.0)&A701V_S(1.0)&K417N_S(0.92)	98.4	NA	NA
Beta	COV025347	Instituto de Salud Global - ISGLOBAL (CAT_ISG)	2021-02-25	RNA			16			13.9	0.9685	1486	Illumina MiSeq	ARTICV3 + Nextera Flex	hCoV-19/Mozambique/MZ-L-IBV-97025347/2021	EPI_ISL_2442111	ERR6018105	Female	46-60	Manhiça	Maputo	Mozambique	B.1.351	E484K_S(1.0)&N501Y_S(1.0)&D614G_S(1.0)&A701V_S(1.0)&K417N_S(0.92)	92.71	NA	NA
Beta	COV025349	Instituto de Salud Global - ISGLOBAL (CAT_ISG)	2021-02-01	RNA			9			63	0.9898	1211	Illumina MiSeq	ARTICV3 + Nextera Flex	hCoV-19/Mozambique/MZ-L-IBV-97025349/2021	EPI_ISL_2442112	ERR6018107	Male	15-30	Manhiça	Maputo	Mozambique	B.1.351	L18F_S(0.99)&E484K_S(1.0)&N501Y_S(1.0)&D614G_S(1.0)&A701V_S(1.0)&K417N_S(0.9)	98.61	NA	NA
Beta	COV025350	Instituto de Salud Global - ISGLOBAL (CAT_ISG)	2021-01-14	RNA			8			117.5	0.9901	1246	Illumina MiSeq	ARTICV3 + Nextera Flex	hCoV-19/Mozambique/MZ-L-IBV-97025350/2021	EPI_ISL_2442113	ERR6018108	Female	15-30	Manhiça	Maputo	Mozambique	B.1.351	E484K_S(1.0)&N501Y_S(1.0)&D614G_S(1.0)&A701V_S(1.0)&K417N_S(0.88)	98.8	NA	NA
Beta	COV025351	Instituto de Salud Global - ISGLOBAL (CAT_ISG)	2021-01-12	RNA			15			38	0.9596	1043	Illumina MiSeq	ARTICV3 + Nextera Flex	hCoV-19/Mozambique/MZ-L-IBV-97025351/2021	EPI_ISL_2442114	ERR6018109	Female	31-45	Manhiça	Maputo	Mozambique	B.1.351	E484K_S(1.0)&N501Y_S(1.0)&D614G_S(1.0)&A701V_S(1.0)&K417N_S(0.93)	97.1	NA	NA
Beta	COV025352	Instituto de Salud Global - ISGLOBAL (CAT_ISG)	2021-01-12	RNA			4			155	0.9905	1345	Illumina MiSeq	ARTICV3 + Nextera Flex	hCoV-19/Mozambique/MZ-L-IBV-97025352/2021	EPI_ISL_2442115	ERR6018110	Male	31-45	Manhiça	Maputo	Mozambique	B.1.351	E484K_S(1.0)&N501Y_S(1.0)&D614G_S(1.0)&A701V_S(1.0)&K417N_S(0.84)	98.85	NA	NA
Beta	COV025353	Instituto de Salud Global - ISGLOBAL (CAT_ISG)	2021-01-14	RNA			9			190	0.9902	1344	Illumina MiSeq	ARTICV3 + Nextera Flex	hCoV-19/Mozambique/MZ-L-IBV-97025353/2021	EPI_ISL_2442116	ERR6018111	Female	46-60	Manhiça	Maputo	Mozambique	B.1.351	L18F_S(1.0)&E484K_S(1.0)&N501Y_S(1.0)&D614G_S(1.0)&A701V_S(1.0)&K417N_S(0.89)	98.77	NA	NA
Beta	COV025354	Instituto de Salud Global - ISGLOBAL (CAT_ISG)	2021-01-14	RNA			4			137.5	0.9872	1386	Illumina MiSeq	ARTICV3 + Nextera Flex	hCoV-19/Mozambique/MZ-L-IBV-97025354/2021	EPI_ISL_2442117	ERR6018112	Female	31-45	Manhiça	Maputo	Mozambique	B.1.351	L18F_S(0.99)&E484K_S(1.0)&N501Y_S(1.0)&D614G_S(1.0)&A701V_S(1.0)&K417N_S(0.82)	98.79	NA	NA
Beta	COV025356	Instituto de Salud Global - ISGLOBAL (CAT_ISG)	2021-01-12	RNA			16			15.6	0.971	1233	Illumina MiSeq	ARTICV3 + Nextera Flex	hCoV-19/Mozambique/MZ-L-IBV-97025356/2021	EPI_ISL_3246523	ERR6018113	Female	31-45	Manhiça	Maputo	Mozambique	B.1.351	L18F_S(0.93)&E484K_S(1.0)&N501Y_S(1.0)&D614G_S(1.0)&A701V_S(1.0)&K417N_S(0.95)	95.64	NA	NA
Beta	COV025357	Instituto de Salud Global - ISGLOBAL (CAT_ISG)	2021-01-08	RNA			18			148.5	0.9821	1399	Illumina MiSeq	ARTICV3 + Nextera Flex	hCoV-19/Mozambique/MZ-L-IBV-97025357/2021	EPI_ISL_2442118	ERR6018114	Female	15-30	Manhiça	Maputo	Mozambique	B.1.351	E484K_S(1.0)&N501Y_S(1.0)&D614G_S(1.0)&A701V_S(1.0)&K417N_S(0.88)	98.84	NA	NA
Beta	COV025358	Instituto de Salud Global - ISGLOBAL (CAT_ISG)	2021-02-09	RNA			18			3.27	0.9059	646	Illumina MiSeq	ARTICV3 + Nextera Flex	hCoV-19/Mozambique/MZ-L-IBV-97025358/2021	EPI_ISL_3246524	ERR6018120	Female	46-60	Manhiça	Maputo	Mozambique	B.1.351	L18F_S(0.08)&E484K_S(1.0)&N501Y_S(0.99)&D614G_S(1.0)&A701V_S(1.0)&K417N_S(0.92)	56.04	NA	NA
Beta	COV025359	Instituto de Salud Global - ISGLOBAL (CAT_ISG)	2021-02-09	RNA			13			35.9	0.9617	1168	Illumina MiSeq	ARTICV3 + Nextera Flex	hCoV-19/Mozambique/MZ-L-IBV-97025359/2021	EPI_ISL_2442119	ERR6018121	Female	46-60	Manhiça	Maputo	Mozambique	B.1.351	E484K_S(1.0)&N501Y_S(1.0)&D614G_S(1.0)&A701V_S(1.0)&K417N_S(0.95)	97.12	NA	NA
Beta	COV025361	Instituto de Salud Global - ISGLOBAL (CAT_ISG)	2021-01-12	RNA			17			5.88	0.9598	999	Illumina MiSeq	ARTICV3 + Nextera Flex	hCoV-19/Mozambique/MZ-L-IBV-97025361/2021	EPI_ISL_2442120	ERR6018123	Female	31-45	Manhiça	Maputo	Mozambique	B.1.351	L18F_S(0.77)&E484K_S(1.0)&N501Y_S(1.0)&D614G_S(1.0)&A701V_S(1.0)&K417N_S(0.97)	92.75	NA	NA
Beta	COV025362	Instituto de Salud Global - ISGLOBAL (CAT_ISG)	2021-01-06	RNA			22			39.4	0.9761	1287	Illumina MiSeq	ARTICV3 + Nextera Flex	hCoV-19/Mozambique/MZ-L-IBV-97025362/2021	EPI_ISL_3246525	ERR6018124	Male	31-45	Manhiça	Maputo	Mozambique	B.1.351	L18F_S(0.35)&E484K_S(0.72)&N501Y_S(0.73)&D614G_S(1.0)&A653V_S(0.19)&A701V_S(0.78)&K417N_S(0.8)	97.09	NA	NA
Beta	COV025364	Instituto de Salud Global - ISGLOBAL (CAT_ISG)	2021-01-23	RNA			15			56	0.9896	1195	Illumina MiSeq	ARTICV3 + Nextera Flex	hCoV-19/Mozambique/MZ-L-IBV-97025364/2021	EPI_ISL_2442122	ERR6018126	Male	46-60	Manhiça	Maputo	Mozambique	B.1.351	L18F_S(0.99)&E484K_S(1.0)&N501Y_S(1.0)&D614G_S(1.0)&A701V_S(1.0)&K417N_S(0.92)	97.65	NA	NA
Beta	COV025365	Instituto de Salud Global - ISGLOBAL (CAT_ISG)	2021-01-23	RNA			11			110.5	0.99	1150	Illumina MiSeq	ARTICV3 + Nextera Flex	hCoV-19/Mozambique/MZ-L-IBV-97025365/2021	EPI_ISL_2442123	ERR6018127	Male	>60	Manhiça	Maputo	Mozambique	B.1.351	E484K_S(1.0)&N501Y_S(1.0)&D614G_S(1.0)&A701V_S(1.0)&K417N_S(0.95)	98.31	NA	NA
Beta	COV025366	Instituto de Salud Global - ISGLOBAL (CAT_ISG)	2021-01-20	RNA			9			102	0.9902	1301	Illumina MiSeq	ARTICV3 + Nextera Flex	hCoV-19/Mozambique/MZ-L-IBV-97025366/2021	EPI_ISL_2442124	ERR6018128	Male	15-30	Manhiça	Maputo	Mozambique	B.1.351	L18F_S(0.58)&E484K_S(1.0)&N501Y_S(1.0)&D614G_S(1.0)&A701V_S(1.0)&K417N_S(0.88)	98.7	NA	NA
Beta	COV025367	Instituto de Salud Global - ISGLOBAL (CAT_ISG)	2021-01-23	RNA			15			37.4	0.9763	1313	Illumina MiSeq	ARTICV3 + Nextera Flex	hCoV-19/Mozambique/MZ-L-IBV-97025367/2021	EPI_ISL_2442125	ERR6018131	Male	15-30	Manhiça	Maputo	Mozambique	B.1.351	L18F_S(0.97)&E484K_S(1.0)&N501Y_S(1.0)&D614G_S(1.0)&A653V_S(1.0)&A701V_S(1.0)&K417N_S(0.96)	96.8	NA	NA
Beta	COV025368	Instituto de Salud Global - ISGLOBAL (CAT_ISG)	2021-01-23	RNA			13			55.5	0.9903	2423	Illumina MiSeq	ARTICV3 + HackFlex	hCoV-19/Mozambique/MZ-L-IBV-97025368/2021	EPI_ISL_2442127	ERR6018132	Male	<15	Manhiça	Maputo	Mozambique	B.1.351	L18F_S(1.0)&E484K_S(1.0)&N501Y_S(1.0)&D614G_S(1.0)&A701V_S(1.0)&K417N_S(0.96)	97.81	NA	NA
Beta	COV025370	Instituto de Salud Global - ISGLOBAL (CAT_ISG)	2021-01-24	RNA			14			63.5	0.9746	1064	Illumina MiSeq	ARTICV3 + Nextera Flex	hCoV-19/Mozambique/MZ-L-IBV-97025370/2021	EPI_ISL_2442126	ERR6018133	Female	15-30	Manhiça	Maputo	Mozambique	B.1.351	E484K_S(1.0)&N501Y_S(1.0)&D614G_S(1.0)&A701V_S(1.0)&K417N_S(0.93)	97.97	NA	NA
Beta	COV025371	Instituto de Salud Global - ISGLOBAL (CAT_ISG)	2021-01-23	RNA			8			158.5	0.9898	1273	Illumina MiSeq	ARTICV3 + HackFlex	hCoV-19/Mozambique/MZ-L-IBV-97025371/2021	EPI_ISL_2442128	ERR6018134	Female	15-30	Manhiça	Maputo	Mozambique	B.1.351	L18F_S(1.0)&E484K_S(1.0)&N501Y_S(1.0)&D614G_S(1.0)&A701V_S(1.0)&K417N_S(0.93)	98.58	NA	NA
Beta	COV025373	Instituto de Salud Global - ISGLOBAL (CAT_ISG)	2021-01-23	RNA		24.19	12			51.5	0.9809	1202	Illumina MiSeq	ARTICV3 + Nextera Flex	hCoV-19/Mozambique/MZ-L-IBV-97025373/2021	EPI_ISL_2442129	ERR6018135	Female	31-45	Manhiça	Maputo	Mozambique	B.1.351	L18F_S(1.0)&E484K_S(1.0)&N501Y_S(1.0)&D614G_S(1.0)&A701V_S(1.0)&K417N_S(0.93)	98.03	NA	NA
Beta	COV025374	Instituto de Salud Global - ISGLOBAL (CAT_ISG)	2021-01-23	RNA		21.35	10			88.5	0.9741	1481	Illumina MiSeq	ARTICV3 + Nextera Flex	hCoV-19/Mozambique/MZ-L-IBV-97025374/2021	EPI_ISL_2442130	ERR6018136	Male	<15	Manhiça	Maputo	Mozambique	B.1.351	L18F_S(1.0)&E484K_S(1.0)&N501Y_S(1.0)&D614G_S(1.0)&A701V_S(1.0)&K417N_S(0.86)	98.21	NA	NA
Beta	COV025375	Instituto de Salud Global - ISGLOBAL (CAT_ISG)	2021-01-23	RNA		32.35	17			1	0.9051	734	Illumina MiSeq	ARTICV3 + Nextera Flex	hCoV-19/Mozambique/MZ-L-IBV-97025375/2021	EPI_ISL_3246526	ERR6018137	Female	31-45	Manhiça	Maputo	Mozambique	B.1.351	D614G_S(1.0)&A701V_S(1.0)&K417N_S(0.99)	66.48	NA	NA
Beta	COV025376	Instituto de Salud Global - ISGLOBAL (CAT_ISG)	2021-01-23	RNA		29.31	17			3.2	0.9472	1171	Illumina MiSeq	ARTICV3 + Nextera Flex	hCoV-19/Mozambique/MZ-L-IBV-97025376/2021	EPI_ISL_2442131	ERR6018138	Female	46-60	Manhiça	Maputo	Mozambique	B.1.351	L18F_S(0.89)&E484K_S(0.99)&N501Y_S(0.99)&D614G_S(1.0)&A701V_S(1.0)&K417N_S(0.96)	87.86	NA	NA
Beta	COV025377	Instituto de Salud Global - ISGLOBAL (CAT_ISG)	2021-01-23	RNA		17.01	5			152.5	0.9905	1494	Illumina MiSeq	ARTICV3 + Nextera Flex	hCoV-19/Mozambique/MZ-L-IBV-97025377/2021	EPI_ISL_2442132	ERR6018143	Female	31-45	Manhiça	Maputo	Mozambique	B.1.351	L18F_S(1.0)&E484K_S(1.0)&N501Y_S(1.0)&D614G_S(1.0)&A653V_S(1.0)&A701V_S(1.0)&K417N_S(0.87)	98.58	NA	NA
Beta	COV025378	Instituto de Salud Global - ISGLOBAL (CAT_ISG)	2021-01-23	RNA		25.28	14			31.4	0.9507	1178	Illumina MiSeq	ARTICV3 + Nextera Flex	hCoV-19/Mozambique/MZ-L-IBV-97025378/2021	EPI_ISL_2442133	ERR6018145	Male	46-60	Manhiça	Maputo	Mozambique	B.1.351	E484K_S(1.0)&N501Y_S(1.0)&D614G_S(1.0)&A701V_S(1.0)&K417N_S(0.92)	97.57	NA	NA
Beta	COV025380	Instituto de Salud Global - ISGLOBAL (CAT_ISG)	2021-01-24	RNA		25.12	16			38.5	0.9737	1480	Illumina MiSeq	ARTICV3 + Nextera Flex	hCoV-19/Mozambique/MZ-L-IBV-97025380/2021	EPI_ISL_2442135	ERR6018147	Female	15-30	Manhiça	Maputo	Mozambique	B.1.351	E484K_S(1.0)&N501Y_S(1.0)&D614G_S(1.0)&A701V_S(1.0)&K417N_S(0.94)	97.58	NA	NA
Beta	COV025381	Instituto de Salud Global - ISGLOBAL (CAT_ISG)	2021-01-24	RNA		11.03	2			147.5	0.9906	1308	Illumina MiSeq	ARTICV3 + Nextera Flex	hCoV-19/Mozambique/MZ-L-IBV-97025381/2021	EPI_ISL_2442136	ERR6018148	Female	15-30	Manhiça	Maputo	Mozambique	B.1.351	L18F_S(1.0)&E484K_S(1.0)&N501Y_S(1.0)&D614G_S(1.0)&A701V_S(1.0)&K417N_S(0.81)	98.71	NA	NA
Beta	COV025383	Instituto de Salud Global - ISGLOBAL (CAT_ISG)	2021-01-24	RNA		26.83	16			11	0.9435	1155	Illumina MiSeq	ARTICV3 + Nextera Flex	hCoV-19/Mozambique/MZ-L-IBV-97025383/2021	EPI_ISL_3246527	ERR6018149	Male	15-30	Manhiça	Maputo	Mozambique	B.1.351	E484K_S(1.0)&N501Y_S(1.0)&D614G_S(1.0)&A701V_S(1.0)&K417N_S(0.94)	95.79	NA	NA
Beta	COV025385	Instituto de Salud Global - ISGLOBAL (CAT_ISG)	2021-01-24	RNA		24.15	14			89	0.9835	1345	Illumina MiSeq	ARTICV3 + Nextera Flex	hCoV-19/Mozambique/MZ-L-IBV-97025385/2021	EPI_ISL_2442137	ERR6018150	Female	15-30	Manhiça	Maputo	Mozambique	B.1.351	L18F_S(0.99)&E484K_S(1.0)&N501Y_S(1.0)&D614G_S(1.0)&A701V_S(1.0)&K417N_S(0.92)	97.68	NA	NA
Beta	COV025386	Instituto de Salud Global - ISGLOBAL (CAT_ISG)	2021-01-24	RNA		22.91	14			80	0.9875	1461	Illumina MiSeq	ARTICV3 + Nextera Flex	hCoV-19/Mozambique/MZ-L-IBV-97025386/2021	EPI_ISL_2442138	ERR6018151	Male	15-30	Manhiça	Maputo	Mozambique	B.1.351	L18F_S(0.71)&E484K_S(1.0)&N501Y_S(1.0)&D614G_S(1.0)&A701V_S(1.0)&K417N_S(0.89)	98.08	NA	NA
Beta	COV025387	Instituto de Salud Global - ISGLOBAL (CAT_ISG)	2021-01-24	RNA		26.06	16			22.85	0.9438	1156	Illumina MiSeq	ARTICV3 + Nextera Flex	hCoV-19/Mozambique/MZ-L-IBV-97025387/2021	EPI_ISL_2442139	ERR6018156	Female	15-30	Manhiça	Maputo	Mozambique	B.1.351	E484K_S(1.0)&N501Y_S(1.0)&D614G_S(1.0)&A701V_S(1.0)&K417N_S(0.94)	96.93	NA	NA
Beta	COV025388	Instituto de Salud Global - ISGLOBAL (CAT_ISG)	2021-01-24	RNA		18.56	7			123	0.9895	1409	Illumina MiSeq	ARTICV3 + Nextera Flex	hCoV-19/Mozambique/MZ-L-IBV-97025388/2021	EPI_ISL_2442140	ERR6018158	Male	15-30	Manhiça	Maputo	Mozambique	B.1.351	L18F_S(1.0)&E484K_S(1.0)&N501Y_S(1.0)&D614G_S(1.0)&A701V_S(1.0)&K417N_S(0.87)	98.55	NA	NA
Beta	COV025389	Instituto de Salud Global - ISGLOBAL (CAT_ISG)	2021-01-24	RNA		24.86	13			36.25	0.976	1657	Illumina MiSeq	ARTICV3 + Nextera Flex	hCoV-19/Mozambique/MZ-L-IBV-97025389/2021	EPI_ISL_3246528	ERR6018159	Male	15-30	Manhiça	Maputo	Mozambique	B.1.351	L18F_S(0.95)&E484K_S(1.0)&N501Y_S(1.0)&D614G_S(1.0)&A701V_S(1.0)&K417N_S(0.94)	97.63	NA	NA
Beta	COV025390	Instituto de Salud Global - ISGLOBAL (CAT_ISG)	2021-01-24	RNA		28.01	16			8	0.9463	1190	Illumina MiSeq	ARTICV3 + Nextera Flex	hCoV-19/Mozambique/MZ-L-IBV-97025390/2021	EPI_ISL_2442141	ERR6018160	Male	15-30	Manhiça	Maputo	Mozambique	B.1.351	L18F_S(0.77)&E484K_S(0.99)&N501Y_S(0.99)&D614G_S(1.0)&A701V_S(1.0)&K417N_S(0.97)	93.53	NA	NA
Beta	COV025391	Instituto de Salud Global - ISGLOBAL (CAT_ISG)	2021-01-24	RNA		26.45	16			25.95	0.9845	1579	Illumina MiSeq	ARTICV3 + Nextera Flex	hCoV-19/Mozambique/MZ-L-IBV-97025391/2021	EPI_ISL_2442142	ERR6018161	Female	15-30	Manhiça	Maputo	Mozambique	B.1.351	L18F_S(0.16)&E484K_S(1.0)&N501Y_S(1.0)&D614G_S(1.0)&A701V_S(1.0)&K417N_S(0.97)	97	NA	NA
Beta	COV025393	Instituto de Salud Global - ISGLOBAL (CAT_ISG)	2021-01-25	RNA		28.62	16			10.7	0.9403	1148	Illumina MiSeq	ARTICV3 + Nextera Flex	hCoV-19/Mozambique/MZ-L-IBV-97025393/2021	EPI_ISL_2442143	ERR6018162	Female	31-45	Manhiça	Maputo	Mozambique	B.1.351	E484K_S(1.0)&N501Y_S(1.0)&D614G_S(1.0)&A701V_S(1.0)&K417N_S(0.96)	91.25	NA	NA
Beta	COV025394	Instituto de Salud Global - ISGLOBAL (CAT_ISG)	2021-01-24	RNA		29.76	19			3.2	0.9341	1114	Illumina MiSeq	ARTICV3 + Nextera Flex	hCoV-19/Mozambique/MZ-L-IBV-97025394/2021	EPI_ISL_2442144	ERR6018163	Female	15-30	Manhiça	Maputo	Mozambique	B.1.351	E484K_S(1.0)&N501Y_S(0.99)&D614G_S(1.0)&A701V_S(1.0)&K417N_S(0.96)	88.7	NA	NA
Beta	COV025395	Instituto de Salud Global - ISGLOBAL (CAT_ISG)	2021-01-24	RNA		14.64	4			150.5	0.9906	1581	Illumina MiSeq	ARTICV3 + Nextera Flex	hCoV-19/Mozambique/MZ-L-IBV-97025395/2021	EPI_ISL_2442145	ERR6018164	Male	15-30	Manhiça	Maputo	Mozambique	B.1.351	L18F_S(1.0)&E484K_S(1.0)&N501Y_S(1.0)&D614G_S(1.0)&A701V_S(1.0)&K417N_S(0.84)	98.58	NA	NA
Beta	COV025396	Instituto de Salud Global - ISGLOBAL (CAT_ISG)	2021-01-24	RNA		21.86	10			80	0.988	1292	Illumina MiSeq	ARTICV3 + Nextera Flex	hCoV-19/Mozambique/MZ-L-IBV-97025396/2021	EPI_ISL_2442146	ERR6018168	Male	15-30	Manhiça	Maputo	Mozambique	B.1.351	L18F_S(1.0)&E484K_S(1.0)&N501Y_S(1.0)&D614G_S(1.0)&A701V_S(1.0)&K417N_S(0.89)	98.31	NA	NA
Beta	COV025398	Instituto de Salud Global - ISGLOBAL (CAT_ISG)	2021-01-25	RNA		17.27	7			143.5	0.9905	1212	Illumina MiSeq	ARTICV3 + Nextera Flex	hCoV-19/Mozambique/MZ-L-IBV-97025398/2021	EPI_ISL_3246531	ERR6018171	Male	46-60	Manhiça	Maputo	Mozambique	B.1.351	E484K_S(1.0)&N501Y_S(1.0)&D614G_S(1.0)&A701V_S(1.0)&K417N_S(0.85)	98.54	NA	NA
Beta	COV025399	Instituto de Salud Global - ISGLOBAL (CAT_ISG)	2021-01-22	RNA		21.35	14			82	0.9791	1431	Illumina MiSeq	ARTICV3 + Nextera Flex	hCoV-19/Mozambique/MZ-L-IBV-97025399/2021	EPI_ISL_2442147	ERR6018173	Male	>60	Manhiça	Maputo	Mozambique	B.1.351	E484K_S(1.0)&N501Y_S(1.0)&D614G_S(1.0)&A701V_S(1.0)&K417N_S(0.94)	98.23	NA	NA
Beta	COV025401	Instituto de Salud Global - ISGLOBAL (CAT_ISG)	2021-01-13	RNA		18.35	19			90.5	0.9905	1232	Illumina MiSeq	ARTICV3 + Nextera Flex	hCoV-19/Mozambique/MZ-L-IBV-97025401/2021	EPI_ISL_2442148	ERR6018174	Male	15-30	Manhiça	Maputo	Mozambique	B.1.351	L18F_S(0.99)&E484K_S(0.99)&N501Y_S(1.0)&D614G_S(1.0)&A701V_S(1.0)&K417N_S(0.91)	98.57	NA	NA
Beta	COV025402	Instituto de Salud Global - ISGLOBAL (CAT_ISG)	2021-01-19	RNA		19.3	19			129.5	0.9905	1617	Illumina MiSeq	ARTICV3 + Nextera Flex	hCoV-19/Mozambique/MZ-L-IBV-97025402/2021	EPI_ISL_2442149	ERR6018175	Female	15-30	Manhiça	Maputo	Mozambique	B.1.351	L18F_S(1.0)&E484K_S(1.0)&N501Y_S(1.0)&D614G_S(1.0)&A701V_S(1.0)&K417N_S(0.9)	98.5	NA	NA
Beta	COV025403	Instituto de Salud Global - ISGLOBAL (CAT_ISG)	2021-01-21	RNA		23.34	20			58.5	0.9828	1267	Illumina MiSeq	ARTICV3 + Nextera Flex	hCoV-19/Mozambique/MZ-L-IBV-97025403/2021	EPI_ISL_2442150	ERR6018176	Female	31-45	Manhiça	Maputo	Mozambique	B.1.351	E484K_S(1.0)&N501Y_S(1.0)&D614G_S(1.0)&A701V_S(1.0)&K417N_S(0.92)	97.73	NA	NA
Beta	COV025405	Instituto de Salud Global - ISGLOBAL (CAT_ISG)	2021-01-05	RNA		12.24	18			140.5	0.9897	1291	Illumina MiSeq	ARTICV3 + Nextera Flex	hCoV-19/Mozambique/MZ-L-IBV-97025405/2021	EPI_ISL_2442152	ERR6018178	Male	15-30	Manhiça	Maputo	Mozambique	B.1.351	E484K_S(1.0)&N501Y_S(1.0)&D614G_S(1.0)&A701V_S(1.0)&K417N_S(0.8)	98.71	NA	NA
Beta	COV025409	Instituto de Salud Global - ISGLOBAL (CAT_ISG)	2021-01-13	RNA		14.71	11			159	0.9836	1362	Illumina MiSeq	ARTICV3 + Nextera Flex	hCoV-19/Mozambique/MZ-L-IBV-97025409/2021	EPI_ISL_2442155	ERR6018191	Female	31-45	Manhiça	Maputo	Mozambique	B.1.351	D614G_S(1.0)&A701V_S(1.0)&K417N_S(1.0)	98.52	NA	NA
Beta	COV025410	Instituto de Salud Global - ISGLOBAL (CAT_ISG)	2021-01-13	RNA		21.42	20			101	0.9902	1379	Illumina MiSeq	ARTICV3 + Nextera Flex	hCoV-19/Mozambique/MZ-L-IBV-97025410/2021	EPI_ISL_2442156	ERR6018195	Male	46-60	Manhiça	Maputo	Mozambique	B.1.351	E484K_S(1.0)&N501Y_S(1.0)&D614G_S(1.0)&A653V_S(0.06)&A701V_S(1.0)&K417N_S(0.88)	98.55	NA	NA
Beta	COV025411	Instituto de Salud Global - ISGLOBAL (CAT_ISG)	2021-01-13	RNA		23.86	22			47	0.9826	1102	Illumina MiSeq	ARTICV3 + Nextera Flex	hCoV-19/Mozambique/MZ-L-IBV-97025411/2021	EPI_ISL_2442157	ERR6018196	Female	<15	Manhiça	Maputo	Mozambique	B.1.351	E484K_S(1.0)&N501Y_S(1.0)&D614G_S(1.0)&A701V_S(1.0)&K417N_S(0.96)	98.35	NA	NA
Beta	COV025412	Instituto de Salud Global - ISGLOBAL (CAT_ISG)	2021-01-13	RNA		19.28	16			134	0.9823	1278	Illumina MiSeq	ARTICV3 + Nextera Flex	hCoV-19/Mozambique/MZ-L-IBV-97025412/2021	EPI_ISL_2442158	ERR6018198	Female	15-30	Manhiça	Maputo	Mozambique	B.1.351	L18F_S(1.0)&E484K_S(1.0)&N501Y_S(1.0)&D614G_S(1.0)&A701V_S(1.0)&K417N_S(0.88)	98.83	NA	NA
Beta	COV025413	Instituto de Salud Global - ISGLOBAL (CAT_ISG)	2021-01-21	RNA		20.83	20			95.5	0.9885	1394	Illumina MiSeq	ARTICV3 + Nextera Flex	hCoV-19/Mozambique/MZ-L-IBV-97025413/2021	EPI_ISL_2442159	ERR6018199	Male	31-45	Manhiça	Maputo	Mozambique	B.1.351	E484K_S(1.0)&N501Y_S(1.0)&D614G_S(1.0)&A701V_S(1.0)&K417N_S(0.9)	98.69	NA	NA
Beta	COV025414	Instituto de Salud Global - ISGLOBAL (CAT_ISG)	2021-01-13	RNA		25.35	2			35	0.9562	1108	Illumina MiSeq	ARTICV3 + Nextera Flex	hCoV-19/Mozambique/MZ-L-IBV-97025414/2021	EPI_ISL_2442160	ERR6018200	Male	<15	Manhiça	Maputo	Mozambique	B.1.351	E484K_S(1.0)&N501Y_S(0.99)&D614G_S(1.0)&A701V_S(1.0)&K417N_S(0.97)	97.21	NA	NA
Beta	COV025415	Instituto de Salud Global - ISGLOBAL (CAT_ISG)	2021-01-13	RNA		18.86	17			112	0.9827	1369	Illumina MiSeq	ARTICV3 + Nextera Flex	hCoV-19/Mozambique/MZ-L-IBV-97025415/2021	EPI_ISL_2442161	ERR6018207	Male	31-45	Manhiça	Maputo	Mozambique	B.1.351	L18F_S(1.0)&E484K_S(1.0)&N501Y_S(1.0)&D614G_S(1.0)&A701V_S(1.0)&K417N_S(0.9)	98.68	NA	NA
Beta	COV025416	Instituto de Salud Global - ISGLOBAL (CAT_ISG)	2021-01-15	RNA		17.09	20			132	0.9905	1319	Illumina MiSeq	ARTICV3 + Nextera Flex	hCoV-19/Mozambique/MZ-L-IBV-97025416/2021	EPI_ISL_2442162	ERR6018212	Male	15-30	Manhiça	Maputo	Mozambique	B.1.351	E484K_S(1.0)&N501Y_S(1.0)&D614G_S(1.0)&A701V_S(1.0)&K417N_S(0.86)	98.77	NA	NA
Beta	COV025417	Instituto de Salud Global - ISGLOBAL (CAT_ISG)	2021-01-14	RNA		10.24	17			173	0.9888	1246	Illumina MiSeq	ARTICV3 + Nextera Flex	hCoV-19/Mozambique/MZ-L-IBV-97025417/2021	EPI_ISL_2442163	ERR6018216	Male	15-30	Manhiça	Maputo	Mozambique	B.1.351	E484K_S(1.0)&N501Y_S(1.0)&D614G_S(1.0)&A701V_S(1.0)&K417N_S(0.84)	98.77	NA	NA
Beta	COV025418	Instituto de Salud Global - ISGLOBAL (CAT_ISG)	2021-01-24	RNA		26.67	15			21.5	0.9363	1073	Illumina MiSeq	ARTICV3 + Nextera Flex	hCoV-19/Mozambique/MZ-L-IBV-97025418/2021	EPI_ISL_2442164	ERR6018217	Female	15-30	Manhiça	Maputo	Mozambique	B.1.351	E484K_S(1.0)&N501Y_S(1.0)&D614G_S(1.0)&A701V_S(1.0)&K417N_S(0.96)	96.98	NA	NA
Beta	COV025419	Instituto de Salud Global - ISGLOBAL (CAT_ISG)	2021-01-18	RNA		19.04	15			116	0.9905	1317	Illumina MiSeq	ARTICV3 + Nextera Flex	hCoV-19/Mozambique/MZ-L-IBV-97025419/2021	EPI_ISL_2442165	ERR6018220	Male	31-45	Manhiça	Maputo	Mozambique	B.1.351	L18F_S(1.0)&E484K_S(1.0)&N501Y_S(1.0)&D614G_S(1.0)&A701V_S(1.0)&K417N_S(0.87)	98.46	NA	NA
Beta	COV025420	Instituto de Salud Global - ISGLOBAL (CAT_ISG)	2021-01-19	RNA		25.06	13			32.25	0.9377	883	Illumina MiSeq	ARTICV3 + Nextera Flex	hCoV-19/Mozambique/MZ-L-IBV-97025420/2021	EPI_ISL_2442166	ERR6018221	Female	46-60	Manhiça	Maputo	Mozambique	B.1.351	E484K_S(1.0)&N501Y_S(1.0)&D614G_S(1.0)&A701V_S(1.0)&K417N_S(0.95)	97.34	NA	NA
Beta	COV025421	Instituto de Salud Global - ISGLOBAL (CAT_ISG)	2021-01-17	RNA		20.49	10			105.5	0.9894	1350	Illumina MiSeq	ARTICV3 + Nextera Flex	hCoV-19/Mozambique/MZ-L-IBV-97025421/2021	EPI_ISL_2442167	ERR6018222	Female	>60	Manhiça	Maputo	Mozambique	B.1.351	L18F_S(0.99)&E484K_S(1.0)&N501Y_S(1.0)&D614G_S(1.0)&A701V_S(1.0)&K417N_S(0.94)	98.24	NA	NA
Beta	COV025422	Instituto de Salud Global - ISGLOBAL (CAT_ISG)	2021-01-19	RNA		14.71	3			144	0.9811	1644	Illumina MiSeq	ARTICV3 + Nextera Flex	hCoV-19/Mozambique/MZ-L-IBV-97025422/2021	EPI_ISL_2442168	ERR6018227	Male	15-30	Manhiça	Maputo	Mozambique	B.1.351	E484K_S(1.0)&N501Y_S(1.0)&D614G_S(1.0)&A701V_S(1.0)	98.78	NA	NA
Beta	COV025423	Instituto de Salud Global - ISGLOBAL (CAT_ISG)	2021-01-19	RNA		11.99	3			130	0.9904	1253	Illumina MiSeq	ARTICV3 + Nextera Flex	hCoV-19/Mozambique/MZ-L-IBV-97025423/2021	EPI_ISL_2442169	ERR6018229	Female		Manhiça	Maputo	Mozambique	B.1.351	E484K_S(1.0)&N501Y_S(1.0)&D614G_S(1.0)&A701V_S(1.0)&K417N_S(0.85)	99.1	NA	NA
Beta	COV025424	Instituto de Salud Global - ISGLOBAL (CAT_ISG)	2021-01-19	RNA		26.24	16			26.5	0.9701	1205	Illumina MiSeq	ARTICV3 + Nextera Flex	hCoV-19/Mozambique/MZ-L-IBV-97025424/2021	EPI_ISL_2442170	ERR6018237	Female	31-45	Manhiça	Maputo	Mozambique	B.1.351	E484K_S(0.99)&N501Y_S(1.0)&D614G_S(1.0)&A701V_S(1.0)&K417N_S(0.95)	96.12	NA	NA
Beta	COV025425	Instituto de Salud Global - ISGLOBAL (CAT_ISG)	2021-01-19	RNA		23.17	11			43.75	0.9825	1328	Illumina MiSeq	ARTICV3 + Nextera Flex	hCoV-19/Mozambique/MZ-L-IBV-97025425/2021	EPI_ISL_2442171	ERR6018239	Male	31-45	Manhiça	Maputo	Mozambique	B.1.351	E484K_S(1.0)&N501Y_S(1.0)&D614G_S(1.0)&A701V_S(1.0)&K417N_S(0.96)	98.02	NA	NA
Beta	COV025426	Instituto de Salud Global - ISGLOBAL (CAT_ISG)	2021-01-05	RNA		15.3	20			146.5	0.9905	1199	Illumina MiSeq	ARTICV3 + Nextera Flex	hCoV-19/Mozambique/MZ-L-IBV-97025426/2021	EPI_ISL_2442172	ERR6018240	Male	>60	Manhiça	Maputo	Mozambique	B.1.351	E484K_S(1.0)&N501Y_S(1.0)&D614G_S(1.0)&A701V_S(1.0)&K417N_S(0.92)	98.74	NA	NA
Beta	COV025427	Instituto de Salud Global - ISGLOBAL (CAT_ISG)	2020-12-30	RNA		13.63	20			163.5	0.9905	1277	Illumina MiSeq	ARTICV3 + Nextera Flex	hCoV-19/Mozambique/MZ-L-IBV-97025427/2020	EPI_ISL_2442173	ERR6018242	Male	15-30	Manhiça	Maputo	Mozambique	B.1.351	E484K_S(1.0)&N501Y_S(1.0)&D614G_S(1.0)&A701V_S(1.0)&K417N_S(0.88)	98.56	NA	NA
Beta	COV025428	Instituto de Salud Global - ISGLOBAL (CAT_ISG)	2021-01-14	RNA		16.6	22			143	0.9905	1315	Illumina MiSeq	ARTICV3 + Nextera Flex	hCoV-19/Mozambique/MZ-L-IBV-97025428/2021	EPI_ISL_2442174	ERR6018243	Female	31-45	Manhiça	Maputo	Mozambique	B.1.351	E484K_S(1.0)&N501Y_S(1.0)&D614G_S(1.0)&A701V_S(1.0)&K417N_S(0.84)	98.75	NA	NA
Beta	COV025429	Instituto de Salud Global - ISGLOBAL (CAT_ISG)	2021-01-14	RNA		17.31	18			112.5	0.9906	1409	Illumina MiSeq	ARTICV3 + Nextera Flex	hCoV-19/Mozambique/MZ-L-IBV-97025429/2021	EPI_ISL_2442175	ERR6018248	Male	15-30	Manhiça	Maputo	Mozambique	B.1.351	E484K_S(1.0)&N501Y_S(1.0)&D614G_S(1.0)&A701V_S(1.0)&K417N_S(0.87)	98.72	NA	NA
Beta	COV025430	Instituto de Salud Global - ISGLOBAL (CAT_ISG)	2021-01-15	RNA		18.58	22			125.5	0.9905	1355	Illumina MiSeq	ARTICV3 + Nextera Flex	hCoV-19/Mozambique/MZ-L-IBV-97025430/2021	EPI_ISL_2442176	ERR6018252	Female	46-60	Manhiça	Maputo	Mozambique	B.1.351	E484K_S(1.0)&N501Y_S(1.0)&D614G_S(1.0)&A701V_S(1.0)&K417N_S(0.85)	98.73	NA	NA
Beta	COV025431	Instituto de Salud Global - ISGLOBAL (CAT_ISG)	2021-01-14	RNA		13.78	22			141.5	0.9906	1360	Illumina MiSeq	ARTICV3 + Nextera Flex	hCoV-19/Mozambique/MZ-L-IBV-97025431/2021	EPI_ISL_2442177	ERR6018256	Male	15-30	Manhiça	Maputo	Mozambique	B.1.351	L18F_S(1.0)&E484K_S(1.0)&N501Y_S(1.0)&D614G_S(1.0)&A701V_S(1.0)&K417N_S(0.86)	98.51	NA	NA
Beta	COV025433	Instituto de Salud Global - ISGLOBAL (CAT_ISG)	2021-01-15	RNA		16.64	20			162	0.9904	1180	Illumina MiSeq	ARTICV3 + Nextera Flex	hCoV-19/Mozambique/MZ-L-IBV-97025433/2021	EPI_ISL_2442178	ERR6018261	Male	31-45	Manhiça	Maputo	Mozambique	B.1.351	E484K_S(1.0)&N501Y_S(1.0)&D614G_S(1.0)&A701V_S(1.0)&K417N_S(0.79)	98.71	NA	NA
Beta	COV025434	Instituto de Salud Global - ISGLOBAL (CAT_ISG)	2021-01-15	RNA		18.68	21			167	0.9844	1267	Illumina MiSeq	ARTICV3 + Nextera Flex	hCoV-19/Mozambique/MZ-L-IBV-97025434/2021	EPI_ISL_2442179	ERR6018266	Female	15-30	Manhiça	Maputo	Mozambique	B.1.351	L18F_S(1.0)&E484K_S(1.0)&N501Y_S(1.0)&D614G_S(1.0)&A701V_S(1.0)&K417N_S(0.85)	98.71	NA	NA
Beta	COV025440	Instituto de Salud Global - ISGLOBAL (CAT_ISG)	2021-01-08	RNA		13.44	19			170.5	0.9906	1425	Illumina MiSeq	ARTICV3 + Nextera Flex	hCoV-19/Mozambique/MZ-L-IBV-97025440/2021	EPI_ISL_2442185	ERR6018282	Male	31-45	Manhiça	Maputo	Mozambique	B.1.351	E484K_S(1.0)&N501Y_S(1.0)&D614G_S(1.0)&A701V_S(1.0)&K417N_S(0.85)	98.86	NA	NA
Beta	COV025452	Instituto de Salud Global - ISGLOBAL (CAT_ISG)	2021-01-18	RNA		26.54	12			39.35	0.9735	1146	Illumina MiSeq	ARTICV3 + Nextera Flex	hCoV-19/Mozambique/MZ-L-IBV-97025452/2021	EPI_ISL_2442193	ERR6018310	Female	>60	Manhiça	Maputo	Mozambique	B.1.351	L18F_S(0.99)&E484K_S(1.0)&N501Y_S(1.0)&D614G_S(1.0)&A701V_S(1.0)&K417N_S(0.94)	93.49	NA	NA
Beta	COV025453	Instituto de Salud Global - ISGLOBAL (CAT_ISG)	2021-01-18	RNA		17.44	5			138	0.9905	1514	Illumina MiSeq	ARTICV3 + Nextera Flex	hCoV-19/Mozambique/MZ-L-IBV-97025453/2021	EPI_ISL_2442194	ERR6018315	Male	31-45	Manhiça	Maputo	Mozambique	B.1.351	E484K_S(1.0)&N501Y_S(1.0)&D614G_S(1.0)&A701V_S(1.0)&K417N_S(0.82)	98.18	NA	NA
Beta	COV025454	Instituto de Salud Global - ISGLOBAL (CAT_ISG)	2021-02-01	RNA		19.37	9			128	0.9905	1883	Illumina MiSeq	ARTICV3 + Nextera Flex	hCoV-19/Mozambique/MZ-L-IBV-97025454/2021	EPI_ISL_2442195	ERR6018321	Female	<15	Manhiça	Maputo	Mozambique	B.1.351	E484K_S(1.0)&N501Y_S(1.0)&D614G_S(1.0)&A701V_S(1.0)&K417N_S(0.88)	98.36	NA	NA
Beta	COV025455	Instituto de Salud Global - ISGLOBAL (CAT_ISG)	2021-02-02	RNA		28.91	13			3.2	0.9267	844	Illumina MiSeq	ARTICV3 + Nextera Flex	hCoV-19/Mozambique/MZ-L-IBV-97025455/2021	EPI_ISL_2442196	ERR6018322	Female	46-60	Manhiça	Maputo	Mozambique	B.1.351	L18F_S(0.85)&E484K_S(0.99)&N501Y_S(1.0)&D614G_S(1.0)&A701V_S(1.0)&K417N_S(0.98)	90.49	NA	NA
Beta	COV025458	Instituto de Salud Global - ISGLOBAL (CAT_ISG)	2021-02-02	RNA		28.09	12			8.2	0.9367	861	Illumina MiSeq	ARTICV3 + Nextera Flex	hCoV-19/Mozambique/MZ-L-IBV-97025458/2021	EPI_ISL_2442197	ERR6018325	Male	46-60	Manhiça	Maputo	Mozambique	B.1.351	E484K_S(1.0)&N501Y_S(1.0)&D614G_S(1.0)&A701V_S(0.99)&K417N_S(0.95)	91.6	NA	NA
Beta	COV025459	Instituto de Salud Global - ISGLOBAL (CAT_ISG)	2021-02-01	RNA		27.21	9			18.5	0.9685	1245	Illumina MiSeq	ARTICV3 + Nextera Flex	hCoV-19/Mozambique/MZ-L-IBV-97025459/2021	EPI_ISL_2442198	ERR6018330	Female	15-30	Manhiça	Maputo	Mozambique	B.1.351	E484K_S(1.0)&N501Y_S(1.0)&D614G_S(1.0)&A701V_S(1.0)&K417N_S(0.95)	97.21	NA	NA
Beta	COV025461	Instituto de Salud Global - ISGLOBAL (CAT_ISG)	2021-02-02	RNA		27.73	13			14.1	0.9489	1042	Illumina MiSeq	ARTICV3 + Nextera Flex	hCoV-19/Mozambique/MZ-L-IBV-97025461/2021	EPI_ISL_2442199	ERR6018334	Male	<15	Manhiça	Maputo	Mozambique	B.1.351	L18F_S(0.96)&E484K_S(0.98)&N501Y_S(0.99)&D614G_S(1.0)&A701V_S(1.0)&K417N_S(0.98)	94.65	NA	NA
Beta	COV025463	Instituto de Salud Global - ISGLOBAL (CAT_ISG)	2021-02-28	RNA		29.55	16			10.1	0.9487	1455	Illumina MiSeq	ARTICV3 + Nextera Flex	hCoV-19/Mozambique/MZ-L-IBV-97025463/2021	EPI_ISL_2442200	ERR6018335	Female	<15	Manhiça	Maputo	Mozambique	B.1.351	E484K_S(1.0)&N501Y_S(1.0)&D614G_S(1.0)&A701V_S(1.0)&K417N_S(0.96)	91.19	NA	NA
Beta	COV025464	Instituto de Salud Global - ISGLOBAL (CAT_ISG)	2021-02-28	RNA		17.11	3			125.5	0.9749	1595	Illumina MiSeq	ARTICV3 + Nextera Flex	hCoV-19/Mozambique/MZ-L-IBV-97025464/2021	EPI_ISL_2442201	ERR6018337	Female	31-45	Manhiça	Maputo	Mozambique	B.1.351	E484K_S(1.0)&N501Y_S(1.0)&D614G_S(1.0)&A701V_S(1.0)&K417N_S(0.81)	98.62	NA	NA
Beta	COV025465	Instituto de Salud Global - ISGLOBAL (CAT_ISG)	2021-02-28	RNA		22.91	10			93	0.9737	1402	Illumina MiSeq	ARTICV3 + Nextera Flex	hCoV-19/Mozambique/MZ-L-IBV-97025465/2021	EPI_ISL_3246529	ERR6018339	Male	31-45	Manhiça	Maputo	Mozambique	B.1.351	E484K_S(1.0)&N501Y_S(1.0)&D614G_S(1.0)&A701V_S(1.0)&K417N_S(0.89)	98.28	NA	NA
Beta	COV025466	Instituto de Salud Global - ISGLOBAL (CAT_ISG)	2021-02-26	RNA		22.3	9			96	0.9832	1244	Illumina MiSeq	ARTICV3 + Nextera Flex	hCoV-19/Mozambique/MZ-L-IBV-97025466/2021	EPI_ISL_2442202	ERR6018343	Male	31-45	Manhiça	Maputo	Mozambique	B.1.351	L18F_S(1.0)&E484K_S(1.0)&N501Y_S(1.0)&D614G_S(1.0)&A701V_S(1.0)&K417N_S(0.88)	98.69	NA	NA
Beta	COV035979	Instituto de Salud Global - ISGLOBAL (CAT_ISG)	2021-05-05	RNA			18.76				0.9958	1250	Illumina MiSeq	ARTICV4 + Nextera Flex	hCoV-19/Mozambique/MZ-L-IBV-97035979/2021	EPI_ISL_17075858		Female	31-45	Manhiça	Maputo	Mozambique	B.1.351	L18F_S(0.93)&E484K_S(0.96)&N501Y_S(0.95)&D614G_S(1.0)&A701V_S(0.87)&K417N_S(0.97)&P681R_S(0.07)	97.23	2	None
Beta	COV035981	Instituto de Salud Global - ISGLOBAL (CAT_ISG)	2021-05-05	RNA			26.31				0.9966	1238	Illumina MiSeq	ARTICV4 + Nextera Flex	hCoV-19/Mozambique/MZ-L-IBV-97035981/2021	EPI_ISL_17075859		Female	<15	Manhiça	Maputo	Mozambique	B.1.351	L18F_S(0.91)&E484K_S(0.91)&N501Y_S(0.91)&D614G_S(1.0)&A701V_S(0.84)&K417N_S(0.95)&L452R_S(0.05)&P681R_S(0.09)	98.32	None	None
Delta	COV035922	Instituto de Salud Global - ISGLOBAL (CAT_ISG)	2021-07-08	RNA			19.6				0.9968	1047	Illumina MiSeq	ARTICV4 + Nextera Flex	hCoV-19/Mozambique/MZ-L-IBV-97035922/2021	EPI_ISL_17075821		Male	46-60	Manhiça	Maputo	Mozambique	B.1.617.2	D614G_S(1.0)&L452R_S(1.0)&P681R_S(1.0)	98.41	None	None
Delta	COV035923	Instituto de Salud Global - ISGLOBAL (CAT_ISG)	2021-06-14	RNA			24.85				0.9324	458	Illumina MiSeq	ARTICV4 + Nextera Flex	hCoV-19/Mozambique/MZ-L-IBV-97035923/2021	EPI_ISL_17112717		Female	<15	Manhiça	Maputo	Mozambique	AY.10	P681R_S(1.0)	82.01	1,2	None
Delta	COV035924	Instituto de Salud Global - ISGLOBAL (CAT_ISG)	2021-07-07	RNA			24.2				0.9968	995	Illumina MiSeq	ARTICV4 + Nextera Flex	hCoV-19/Mozambique/MZ-L-IBV-97035924/2021	EPI_ISL_17075822		Male	31-45	Manhiça	Maputo	Mozambique	B.1.617.2	D614G_S(1.0)&L452R_S(1.0)&P681R_S(1.0)	98.46	1,3,5	None
Delta	COV035925	Instituto de Salud Global - ISGLOBAL (CAT_ISG)	2021-06-28	RNA			5.78				0.9967	1016	Illumina MiSeq	ARTICV4 + Nextera Flex	hCoV-19/Mozambique/MZ-L-IBV-97035925/2021	EPI_ISL_17075823		Male	46-60	Manhiça	Maputo	Mozambique	B.1.617.2	D614G_S(1.0)&L452R_S(1.0)&P681R_S(1.0)	98.7	2	None
Delta	COV035926	Instituto de Salud Global - ISGLOBAL (CAT_ISG)	2021-07-26	RNA			13				0.9258	66	Illumina MiSeq	ARTICV4 + Nextera Flex	hCoV-19/Mozambique/MZ-L-IBV-97035926/2021	EPI_ISL_17118741		Male	<15	Manhiça	Maputo	Mozambique	AY.10	D614G_S(1.0)&P681R_S(1.0)	72.08	3,4	Asthma
Delta	COV035927	Instituto de Salud Global - ISGLOBAL (CAT_ISG)	2021-07-23	RNA			26				0.9932	1231	Illumina MiSeq	ARTICV4 + Nextera Flex	hCoV-19/Mozambique/MZ-L-IBV-97035927/2021	EPI_ISL_17075824		Female	<15	Manhiça	Maputo	Mozambique	B.1.617.2	D614G_S(1.0)&L452R_S(1.0)&P681R_S(1.0)	96.07	1,2	Asthma
Delta	COV035928	Instituto de Salud Global - ISGLOBAL (CAT_ISG)	2021-07-19	RNA			24.5				0.9967	1016	Illumina MiSeq	ARTICV4 + Nextera Flex	hCoV-19/Mozambique/MZ-L-IBV-97035928/2021	EPI_ISL_17075825		Female	46-60	Manhiça	Maputo	Mozambique	B.1.617.2	D614G_S(1.0)&L452R_S(1.0)&P681R_S(1.0)	98.51	1,3,5	HIV-SIDA
Delta	COV035931	Instituto de Salud Global - ISGLOBAL (CAT_ISG)	2021-07-05	RNA			5.24				0.9911	1445	Illumina MiSeq	ARTICV4 + Nextera Flex	hCoV-19/Mozambique/MZ-L-IBV-97035931/2021	EPI_ISL_17075826		Male	>60	Manhiça	Maputo	Mozambique	B.1.617.2	D614G_S(1.0)&L452R_S(1.0)&P681R_S(1.0)	98.55	None	Hipertension
Delta	COV035932	Instituto de Salud Global - ISGLOBAL (CAT_ISG)	2021-07-05	RNA			12.5				0.9966	1063	Illumina MiSeq	ARTICV4 + Nextera Flex	hCoV-19/Mozambique/MZ-L-IBV-97035932/2021	EPI_ISL_17075827		Female	31-45	Manhiça	Maputo	Mozambique	B.1.617.2	D614G_S(1.0)&L452R_S(1.0)&P681R_S(1.0)	98.3	1	None
Delta	COV035934	Instituto de Salud Global - ISGLOBAL (CAT_ISG)	2021-07-05	RNA			11.91				0.9884	1291	Illumina MiSeq	ARTICV4 + Nextera Flex	hCoV-19/Mozambique/MZ-L-IBV-97035934/2021	EPI_ISL_17075828		Female	31-45	Manhiça	Maputo	Mozambique	B.1.617.2	D614G_S(1.0)&L452R_S(1.0)&P681R_S(0.99)	98.48	1,3,4	Hipertension
Delta	COV035935	Instituto de Salud Global - ISGLOBAL (CAT_ISG)	2021-07-05	RNA			5.91				0.9967	1398	Illumina MiSeq	ARTICV4 + Nextera Flex	hCoV-19/Mozambique/MZ-L-IBV-97035935/2021	EPI_ISL_17075829		Male	31-45	Manhiça	Maputo	Mozambique	B.1.617.2	D614G_S(1.0)&L452R_S(1.0)&P681R_S(1.0)	98.43	1,5	Asthma
Delta	COV035937	Instituto de Salud Global - ISGLOBAL (CAT_ISG)	2021-06-28	RNA			21.11				0.9905	1297	Illumina MiSeq	ARTICV4 + Nextera Flex	hCoV-19/Mozambique/MZ-L-IBV-97035937/2021	EPI_ISL_17075830		Male	>60	Manhiça	Maputo	Mozambique	AY.37	D614G_S(1.0)&L452R_S(1.0)&P681R_S(1.0)	97.87	2,4	Hipertension

Delta	COV035938	Instituto de Salud Global - ISGLOBAL (CAT_ISG)	2021-07-05	RNA			10.3				0.9967	1631	Illumina MiSeq	ARTICV4 + Nextera Flex	hCoV-19/Mozambique/MZ-L-IBV-97035938/2021	EPI_ISL_17075831		Female	<15	Manhiça	Maputo	Mozambique	B.1.617.2	D614G_S(1.0)&L452R_S(1.0)&P681R_S(1.0)	98.48	2	Asthma
Delta	COV035939	Instituto de Salud Global - ISGLOBAL (CAT_ISG)	2021-07-05	RNA			15.63				0.9963	1427	Illumina MiSeq	ARTICV4 + Nextera Flex	hCoV-19/Mozambique/MZ-L-IBV-97035939/2021	EPI_ISL_17075832		Female	31-45	Manhiça	Maputo	Mozambique	B.1.617.2	D614G_S(1.0)&L452R_S(1.0)&P681R_S(1.0)	98.28	1,2,5	HIV-SIDA
Delta	COV035940	Instituto de Salud Global - ISGLOBAL (CAT_ISG)	2021-07-05	RNA			14.97				0.9979	1455	Illumina MiSeq	ARTICV4 + Nextera Flex	hCoV-19/Mozambique/MZ-L-IBV-97035940/2021	EPI_ISL_17075833		Female	31-45	Manhiça	Maputo	Mozambique	B.1.617.2	D614G_S(1.0)&L452R_S(1.0)&P681R_S(1.0)	97.57	1,2,5	HIV-SIDA
Delta	COV035942	Instituto de Salud Global - ISGLOBAL (CAT_ISG)	2021-06-28	RNA			4.41				0.9967	1355	Illumina MiSeq	ARTICV4 + Nextera Flex	hCoV-19/Mozambique/MZ-L-IBV-97035942/2021	EPI_ISL_17075834		Female	>60	Manhiça	Maputo	Mozambique	B.1.617.2	D614G_S(1.0)&L452R_S(1.0)&P681R_S(0.99)	98.45	3	No
Delta	COV035943	Instituto de Salud Global - ISGLOBAL (CAT_ISG)	2021-06-14	RNA			26.08				0.946	808	Illumina MiSeq	ARTICV4 + Nextera Flex	hCoV-19/Mozambique/MZ-L-IBV-97035943/2021	EPI_ISL_17112718		Female	<15	Manhiça	Maputo	Mozambique	B.1.617.2	E484K_S(0.11)&N501Y_S(0.13)&D614G_S(1.0)&L452R_S(1.0)&P681R_S(1.0)&R190S_S(0.22)	89.6	3	No
Delta	COV035947	Instituto de Salud Global - ISGLOBAL (CAT_ISG)	2021-07-07	RNA			25.7				0.9968	1237	Illumina MiSeq	ARTICV4 + Nextera Flex	hCoV-19/Mozambique/MZ-L-IBV-97035947/2021	EPI_ISL_17075835		Male	<15	Manhiça	Maputo	Mozambique	B.1.617.2	D614G_S(1.0)&L452R_S(1.0)&P681R_S(1.0)	98.31	1,3	No
Delta	COV035949	Instituto de Salud Global - ISGLOBAL (CAT_ISG)	2021-07-09	RNA			21.6				0.9967	1424	Illumina MiSeq	ARTICV4 + Nextera Flex	hCoV-19/Mozambique/MZ-L-IBV-97035949/2021	EPI_ISL_17075836		Female	46-60	Manhiça	Maputo	Mozambique	B.1.617.2	D614G_S(1.0)&L452R_S(1.0)&P681R_S(0.99)	98.32	NA	NA
Delta	COV035950	Instituto de Salud Global - ISGLOBAL (CAT_ISG)	2021-07-09	RNA			19.6				0.9968	1392	Illumina MiSeq	ARTICV4 + Nextera Flex	hCoV-19/Mozambique/MZ-L-IBV-97035950/2021	EPI_ISL_17075837		Male	<15	Manhiça	Maputo	Mozambique	B.1.617.2	D614G_S(1.0)&L452R_S(1.0)&P681R_S(1.0)	98.38	NA	NA
Delta	COV035951	Instituto de Salud Global - ISGLOBAL (CAT_ISG)	2021-06-28	RNA			22.07				0.9896	1299	Illumina MiSeq	ARTICV4 + Nextera Flex	hCoV-19/Mozambique/MZ-L-IBV-97035951/2021	EPI_ISL_17118743		Male	15-30	Manhiça	Maputo	Mozambique	B.1.617.2	D614G_S(1.0)&L452R_S(1.0)&P681R_S(1.0)	93.98	NA	NA
Delta	COV035952	Instituto de Salud Global - ISGLOBAL (CAT_ISG)	2021-07-09	RNA			19.8				0.9967	1537	Illumina MiSeq	ARTICV4 + Nextera Flex	hCoV-19/Mozambique/MZ-L-IBV-97035952/2021	EPI_ISL_17075838		Male	15-30	Manhiça	Maputo	Mozambique	B.1.617.2	D614G_S(1.0)&L452R_S(1.0)&P681R_S(1.0)	98.42	NA	NA
Delta	COV035953	Instituto de Salud Global - ISGLOBAL (CAT_ISG)	2021-07-09	RNA			23				0.9964	1354	Illumina MiSeq	ARTICV4 + Nextera Flex	hCoV-19/Mozambique/MZ-L-IBV-97035953/2021	EPI_ISL_17075839		Female	<15	Manhiça	Maputo	Mozambique	B.1.617.2	D614G_S(1.0)&L452R_S(1.0)&P681R_S(1.0)	98.47	1,2	None
Delta	COV035955	Instituto de Salud Global - ISGLOBAL (CAT_ISG)	2021-07-09	RNA			28				0.977	1425	Illumina MiSeq	ARTICV4 + Nextera Flex	hCoV-19/Mozambique/MZ-L-IBV-97035955/2021	EPI_ISL_17075840		Female	15-30	Manhiça	Maputo	Mozambique	B.1.617.2	D614G_S(1.0)&L452R_S(1.0)&P681R_S(1.0)	98.01	NA	NA
Delta	COV035956	Instituto de Salud Global - ISGLOBAL (CAT_ISG)	2021-07-08	RNA			23.1				0.9969	1478	Illumina MiSeq	ARTICV4 + Nextera Flex	hCoV-19/Mozambique/MZ-L-IBV-97035956/2021	EPI_ISL_17075841		Female	31-45	Manhiça	Maputo	Mozambique	B.1.617.2	D614G_S(1.0)&L452R_S(1.0)&P681R_S(1.0)	98.46	NA	NA
Delta	COV035957	Instituto de Salud Global - ISGLOBAL (CAT_ISG)	2021-07-08	RNA			24.6				0.9968	1542	Illumina MiSeq	ARTICV4 + Nextera Flex	hCoV-19/Mozambique/MZ-L-IBV-97035957/2021	EPI_ISL_17075842		Female	15-30	Manhiça	Maputo	Mozambique	B.1.617.2	D614G_S(1.0)&L452R_S(1.0)&P681R_S(1.0)	98.43	1,2,4,5	None
Delta	COV035958	Instituto de Salud Global - ISGLOBAL (CAT_ISG)	2021-07-08	RNA			23.4				0.9967	1236	Illumina MiSeq	ARTICV4 + Nextera Flex	hCoV-19/Mozambique/MZ-L-IBV-97035958/2021	EPI_ISL_17075843		Male	15-30	Manhiça	Maputo	Mozambique	B.1.617.2	D614G_S(1.0)&L452R_S(1.0)&P681R_S(1.0)	98.54	1,2	None
Delta	COV035959	Instituto de Salud Global - ISGLOBAL (CAT_ISG)	2021-07-08	RNA			23.9				0.9967	1329	Illumina MiSeq	ARTICV4 + Nextera Flex	hCoV-19/Mozambique/MZ-L-IBV-97035959/2021	EPI_ISL_17075844		Female	15-30	Manhiça	Maputo	Mozambique	B.1.617.2	D614G_S(1.0)&L452R_S(1.0)&P681R_S(1.0)	98.41	1,2,4,5	None
Delta	COV035960	Instituto de Salud Global - ISGLOBAL (CAT_ISG)	2021-07-08	RNA			20.5				0.9968	1329	Illumina MiSeq	ARTICV4 + Nextera Flex	hCoV-19/Mozambique/MZ-L-IBV-97035960/2021	EPI_ISL_17075845		Male	31-45	Manhiça	Maputo	Mozambique	AY.6	D614G_S(1.0)&L452R_S(1.0)&P681R_S(1.0)	98.39	NA	NA
Delta	COV035961	Instituto de Salud Global - ISGLOBAL (CAT_ISG)	2021-07-06	RNA			18				0.9912	1262	Illumina MiSeq	ARTICV4 + Nextera Flex	hCoV-19/Mozambique/MZ-L-IBV-97035961/2021	EPI_ISL_17075846		Male	31-45	Manhiça	Maputo	Mozambique	B.1.617.2	D614G_S(1.0)&L452R_S(1.0)&P681R_S(1.0)	98.51	2,4,5	None
Delta	COV035962	Instituto de Salud Global - ISGLOBAL (CAT_ISG)	2021-07-06	RNA			25				0.9859	1311	Illumina MiSeq	ARTICV4 + Nextera Flex	hCoV-19/Mozambique/MZ-L-IBV-97035962/2021	EPI_ISL_17075847		Male	46-60	Manhiça	Maputo	Mozambique	B.1.617.2	D614G_S(0.99)&L452R_S(1.0)&P681R_S(1.0)	98.55	2,4	HIV-SIDA
Delta	COV035963	Instituto de Salud Global - ISGLOBAL (CAT_ISG)	2021-07-26	RNA			19				0.9968	1326	Illumina MiSeq	ARTICV4 + Nextera Flex	hCoV-19/Mozambique/MZ-L-IBV-97035963/2021	EPI_ISL_17075848		Female	31-45	Manhiça	Maputo	Mozambique	B.1.617.2	D614G_S(1.0)&L452R_S(1.0)&P681R_S(1.0)	98.05	3,4	HIV-SIDA
Delta	COV035964	Instituto de Salud Global - ISGLOBAL (CAT_ISG)	2021-08-16	RNA			22				0.9655	539	Illumina MiSeq	ARTICV4 + Nextera Flex	hCoV-19/Mozambique/MZ-L-IBV-97035964/2021	EPI_ISL_17075849		Female	15-30	Manhiça	Maputo	Mozambique	B.1.617.2	D614G_S(1.0)&L452R_S(1.0)&P681R_S(1.0)	47.98	1,3,5	None
Delta	COV035968	Instituto de Salud Global - ISGLOBAL (CAT_ISG)	2021-07-08	RNA			26.4				0.9968	1395	Illumina MiSeq	ARTICV4 + Nextera Flex	hCoV-19/Mozambique/MZ-L-IBV-97035968/2021	EPI_ISL_17075850		Male	31-45	Manhiça	Maputo	Mozambique	B.1.617.2	D614G_S(1.0)&L452R_S(1.0)&P681R_S(1.0)	98.49	1	None
Delta	COV035969	Instituto de Salud Global - ISGLOBAL (CAT_ISG)	2021-07-08	RNA			23.1				0.9859	1261	Illumina MiSeq	ARTICV4 + Nextera Flex	hCoV-19/Mozambique/MZ-L-IBV-97035969/2021	EPI_ISL_17075851		Male	31-45	Manhiça	Maputo	Mozambique	B.1.617.2	D614G_S(1.0)&L452R_S(1.0)&P681R_S(1.0)	98.35	1,3	None
Delta	COV035970	Instituto de Salud Global - ISGLOBAL (CAT_ISG)	2021-06-14	RNA			23.98				0.9606	924	Illumina MiSeq	ARTICV4 + Nextera Flex	hCoV-19/Mozambique/MZ-L-IBV-97035970/2021	EPI_ISL_17075852		Male	31-45	Manhiça	Maputo	Mozambique	B.1.617.2	D614G_S(1.0)&L452R_S(1.0)&P681R_S(1.0)	92.43	NA	NA
Delta	COV035972	Instituto de Salud Global - ISGLOBAL (CAT_ISG)	2021-07-01	RNA			3.34				0.9968	1353	Illumina MiSeq	ARTICV4 + Nextera Flex	hCoV-19/Mozambique/MZ-L-IBV-97035972/2021	EPI_ISL_17075853		Female	46-60	Manhiça	Maputo	Mozambique	AY.6	D614G_S(1.0)&L452R_S(1.0)&P681R_S(1.0)	98.36	1,2,5	HIV-SIDA
Delta	COV035973	Instituto de Salud Global - ISGLOBAL (CAT_ISG)	2021-07-01	RNA			4.55				0.9967	1213	Illumina MiSeq	ARTICV4 + Nextera Flex	hCoV-19/Mozambique/MZ-L-IBV-97035973/2021	EPI_ISL_17075854		Male	15-30	Manhiça	Maputo	Mozambique	B.1.617.2	D614G_S(1.0)&L452R_S(1.0)&P681R_S(1.0)	98.35	1	None
Delta	COV035974	Instituto de Salud Global - ISGLOBAL (CAT_ISG)	2021-07-01	RNA			21.01				0.9814	1131	Illumina MiSeq	ARTICV4 + Nextera Flex	hCoV-19/Mozambique/MZ-L-IBV-97035974/2021	EPI_ISL_17112719		Male	31-45	Manhiça	Maputo	Mozambique	B.1.617.2	D614G_S(1.0)&L452R_S(1.0)&P681R_S(1.0)	95.98	1,3	None
Delta	COV035975	Instituto de Salud Global - ISGLOBAL (CAT_ISG)	2021-07-01	RNA			5.24				0.9969	1342	Illumina MiSeq	ARTICV4 + Nextera Flex	hCoV-19/Mozambique/MZ-L-IBV-97035975/2021	EPI_ISL_17075855		Female	15-30	Manhiça	Maputo	Mozambique	B.1.617.2	D614G_S(1.0)&L452R_S(1.0)&P681R_S(1.0)	98.27	1,2,4	None
Delta	COV035976	Instituto de Salud Global - ISGLOBAL (CAT_ISG)	2021-07-01	RNA			4.06				0.9967	1204	Illumina MiSeq	ARTICV4 + Nextera Flex	hCoV-19/Mozambique/MZ-L-IBV-97035976/2021	EPI_ISL_17075856		Female	31-45	Manhiça	Maputo	Mozambique	AY.6	D614G_S(1.0)&L452R_S(1.0)&P681R_S(1.0)	98.34	1,2	None
Delta	COV035977	Instituto de Salud Global - ISGLOBAL (CAT_ISG)	2021-05-05	RNA			23.55				0.9536	1062	Illumina MiSeq	ARTICV4 + Nextera Flex	hCoV-19/Mozambique/MZ-L-IBV-97035977/2021	EPI_ISL_17075857		Female	<15	Manhiça	Maputo	Mozambique	B.1.617.2	D614G_S(1.0)&L452R_S(1.0)&P681R_S(1.0)	87.23	2	None
Delta	COV035983	Instituto de Salud Global - ISGLOBAL (CAT_ISG)	2021-07-01	RNA			5.98				0.9966	795	Illumina MiSeq	ARTICV4 + Nextera Flex	hCoV-19/Mozambique/MZ-L-IBV-97035983/2021	EPI_ISL_17075860		Female	<15	Manhiça	Maputo	Mozambique	AY.6	D614G_S(1.0)&L452R_S(1.0)&P681R_S(1.0)	98.82	2	None
Delta	COV035984	Instituto de Salud Global - ISGLOBAL (CAT_ISG)	2021-07-01	RNA			9.51				0.9916	1123	Illumina MiSeq	ARTICV4 + Nextera Flex	hCoV-19/Mozambique/MZ-L-IBV-97035984/2021	EPI_ISL_17075861		Male	15-30	Manhiça	Maputo	Mozambique	B.1.617.2	D614G_S(1.0)&L452R_S(1.0)&P681R_S(1.0)	98.6	2	None
Delta	COV035985	Instituto de Salud Global - ISGLOBAL (CAT_ISG)	2021-07-02	RNA			7.13				0.9968	1031	Illumina MiSeq	ARTICV4 + Nextera Flex	hCoV-19/Mozambique/MZ-L-IBV-97035985/2021	EPI_ISL_17075862		Male	15-30	Manhiça	Maputo	Mozambique	B.1.617.2	D614G_S(1.0)&L452R_S(1.0)&P681R_S(1.0)	98.79	NA	NA
Delta	COV035986	Instituto de Salud Global - ISGLOBAL (CAT_ISG)	2021-07-02	RNA			7.15				0.9979	1369	Illumina MiSeq	ARTICV4 + Nextera Flex	hCoV-19/Mozambique/MZ-L-IBV-97035986/2021	EPI_ISL_17075863		Male	15-30	Manhiça	Maputo	Mozambique	B.1.617.2	D614G_S(1.0)&L452R_S(1.0)&P681R_S(0.99)	98.48	1,2,4,5	None
Delta	COV035987	Instituto de Salud Global - ISGLOBAL (CAT_ISG)	2021-07-03	RNA			13.26				0.9963	853	Illumina MiSeq	ARTICV4 + Nextera Flex	hCoV-19/Mozambique/MZ-L-IBV-97035987/2021	EPI_ISL_17075864		Male	15-30	Manhiça	Maputo	Mozambique	B.1.617.2	D614G_S(1.0)&L452R_S(1.0)&P681R_S(1.0)	98.15	1,2,5	Asthma
Delta	COV035988	Instituto de Salud Global - ISGLOBAL (CAT_ISG)	2021-07-02	RNA			8.64				0.9967	1104	Illumina MiSeq	ARTICV4 + Nextera Flex	hCoV-19/Mozambique/MZ-L-IBV-97035988/2021	EPI_ISL_17075865		Female	15-30	Manhiça	Maputo	Mozambique	B.1.617.2	D614G_S(1.0)&L452R_S(1.0)&P681R_S(1.0)	98.71	3	None
Delta	COV035991	Instituto de Salud Global - ISGLOBAL (CAT_ISG)	2021-07-13	RNA			18				0.993	1264	Illumina MiSeq	ARTICV4 + Nextera Flex	hCoV-19/Mozambique/MZ-L-IBV-97035991/2021	EPI_ISL_17075866		Female	31-45	Manhiça	Maputo	Mozambique	AY.6	D614G_S(1.0)&L452R_S(1.0)&P681R_S(1.0)	98.71	1,3,5	Asthma
Delta	COV035992	Instituto de Salud Global - ISGLOBAL (CAT_ISG)	2021-07-19	RNA			23				0.9967	1454	Illumina MiSeq	ARTICV4 + Nextera Flex	hCoV-19/Mozambique/MZ-L-IBV-97035992/2021	EPI_ISL_17075867		Male	31-45	Manhiça	Maputo	Mozambique	B.1.617.2	D614G_S(1.0)&L452R_S(1.0)&P681R_S(1.0)	98.6	1,3	None
Delta	COV035993	Instituto de Salud Global - ISGLOBAL (CAT_ISG)	2021-07-27	RNA			15				0.9967	1561	Illumina MiSeq	ARTICV4 + Nextera Flex	hCoV-19/Mozambique/MZ-L-IBV-97035993/2021	EPI_ISL_17075868		Female	<15	Manhiça	Maputo	Mozambique	B.1.617.2	D614G_S(1.0)&L452R_S(1.0)&P681R_S(1.0)	98.56	1,3,5	None
Delta	COV035994	Instituto de Salud Global - ISGLOBAL (CAT_ISG)	2021-06-24	RNA			0				0.9968	1201	Illumina MiSeq	ARTICV4 + Nextera Flex	hCoV-19/Mozambique/MZ-L-IBV-97035994/2021	EPI_ISL_17075869		Male	15-30	Manhiça	Maputo	Mozambique	B.1.617.2	D614G_S(1.0)&L452R_S(1.0)&P681R_S(1.0)	98.57	1,4,5	None
Delta	COV035995	Instituto de Salud Global - ISGLOBAL (CAT_ISG)	2021-07-03	RNA			5.79				0.9967	1225	Illumina MiSeq	ARTICV4 + Nextera Flex	hCoV-19/Mozambique/MZ-L-IBV-97035995/2021	EPI_ISL_17075870		Female	46-60	Manhiça	Maputo	Mozambique	B.1.617.2	D614G_S(1.0)&L452R_S(1.0)&P681R_S(1.0)	98.65	1,2	Hipertension
Delta	COV035997	Instituto de Salud Global - ISGLOBAL (CAT_ISG)	2021-07-13	RNA			22.9				0.9191	408	Illumina MiSeq	ARTICV4 + Nextera Flex	hCoV-19/Mozambique/MZ-L-IBV-97035997/2021	EPI_ISL_17075871		Female	15-30	Manhiça	Maputo	Mozambique	B.1.617.2	E484K_S(0.12)&D614G_S(1.0)&L452R_S(0.82)&P681R_S(1.0)&R190S_S(0.08)	81.01	3	None
Delta	COV035998	Instituto de Salud Global - ISGLOBAL (CAT_ISG)	2021-07-26	RNA			17				0.9967	1142	Illumina MiSeq	ARTICV4 + Nextera Flex	hCoV-19/Mozambique/MZ-L-IBV-97035998/2021	EPI_ISL_17075872		Male	15-30	Manhiça	Maputo	Mozambique	B.1.617.2	D614G_S(1.0)&L452R_S(1.0)&P681R_S(1.0)	98.61	2	None
Delta	COV035999	Instituto de Salud Global - ISGLOBAL (CAT_ISG)	2021-07-01	RNA			11.89				0.9965	1179	Illumina MiSeq	ARTICV4 + Nextera Flex	hCoV-19/Mozambique/MZ-L-IBV-97035999/2021	EPI_ISL_17075873		Female	15-30	Manhiça	Maputo	Mozambique	B.1.617.2	D614G_S(1.0)&L452R_S(1.0)&P681R_S(1.0)	98.47	1,2,5	None
Delta	COV036000	Instituto de Salud Global - ISGLOBAL (CAT_ISG)	2021-07-05	RNA			14.86				0.9973	853	Illumina MiSeq	ARTICV4 + Nextera Flex	hCoV-19/Mozambique/MZ-L-IBV-97036000/2021	EPI_ISL_17075874		Male	46-60	Manhiça	Maputo	Mozambique	B.1.617.2	D614G_S(1.0)&L452R_S(1.0)&P681R_S(1.0)	96.63	3,5	None
Delta	COV036001	Instituto de Salud Global - ISGLOBAL (CAT_ISG)	2021-07-15	RNA			0				0.9967	1068	Illumina MiSeq	ARTICV4 + Nextera Flex	hCoV-19/Mozambique/MZ-L-IBV-97036001/2021	EPI_ISL_17075875		Male	31-45	Manhiça	Maputo	Mozambique	B.1.617.2	D614G_S(1.0)&L452R_S(1.0)&P681R_S(1.0)	98.57	NA	NA
Delta	COV036002	Instituto de Salud Global - ISGLOBAL (CAT_ISG)	2021-06-24	RNA			9.18				0.9968	1077	Illumina MiSeq	ARTICV4 + Nextera Flex	hCoV-19/Mozambique/MZ-L-IBV-97036002/2021	EPI_ISL_17075876		Male	31-45	Manhiça	Maputo	Mozambique	B.1.617.2	D614G_S(1.0)&L452R_S(1.0)&P681R_S(1.0)	98.55	1,2,5	None
Delta	COV036003	Instituto de Salud Global - ISGLOBAL (CAT_ISG)	2021-03-15	RNA			19.34				0.9978	1272	Illumina MiSeq	ARTICV4 + Nextera Flex	hCoV-19/Mozambique/MZ-L-IBV-97036003/2021	EPI_ISL_17075877		Female	31-45	Manhiça	Maputo	Mozambique	B.1.617.2	D614G_S(1.0)&L452R_S(1.0)&P681R_S(1.0)	97.56	2	None
Delta	COV036005	Instituto de Salud Global - ISGLOBAL (CAT_ISG)	2021-07-05	RNA			12.53				0.9967	1494	Illumina MiSeq	ARTICV4 + Nextera Flex	hCoV-19/Mozambique/MZ-L-IBV-97036005/2021	EPI_ISL_17075879		Female		Manhiça	Maputo	Mozambique	B.1.617.2	D614G_S(1.0)&L452R_S(1.0)&P681R_S(1.0)	98.51	1,2	None
Delta	COV036006	Instituto de Salud Global - ISGLOBAL (CAT_ISG)	2021-07-14	RNA			19				0.9966	1039	Illumina MiSeq	ARTICV4 + Nextera Flex	hCoV-19/Mozambique/MZ-L-IBV-97036006/2021	EPI_ISL_17075880		Female	31-45	Manhiça	Maputo	Mozambique	B.1.617.2	D614G_S(1.0)&L452R_S(1.0)&P681R_S(1.0)	98.28	2,4	HIV-SIDA
Delta	COV036007	Instituto de Salud Global - ISGLOBAL (CAT_ISG)	2021-07-19	RNA			25				0.9857	775	Illumina MiSeq	ARTICV4 + Nextera Flex	hCoV-19/Mozambique/MZ-L-IBV-97036007/2021	EPI_ISL_17075881		Male	31-45	Manhiça	Maputo	Mozambique	B.1.617.2	D614G_S(1.0)&L452R_S(1.0)&P681R_S(1.0)	97.83	1,5	None
Delta	COV036008	Instituto de Salud Global - ISGLOBAL (CAT_ISG)	2021-07-12	RNA			16				0.9859	812	Illumina MiSeq	ARTICV4 + Nextera Flex	hCoV-19/Mozambique/MZ-L-IBV-97036008/2021	EPI_ISL_17075882		Female	15-30	Manhiça	Maputo	Mozambique	B.1.617.2	D614G_S(1.0)&L452R_S(0.99)&P681R_S(1.0)	98.38	1,3,5	None
Delta	COV036010	Instituto de Salud Global - ISGLOBAL (CAT_ISG)	2021-07-12	RNA			22				0.9963	891	Illumina MiSeq	ARTICV4 + Nextera Flex	hCoV-19/Mozambique/MZ-L-IBV-97036010/2021	EPI_ISL_17075883		Male	46-60	Manhiça	Maputo	Mozambique	B.1.617.2	D614G_S(1.0)&L452R_S(1.0)&P681R_S(1.0)	98.43	1	HIV-SIDA
Delta	COV036011	Instituto de Salud Global - ISGLOBAL (CAT_ISG)	2021-07-19	RNA			18				0.9892	806	Illumina MiSeq	ARTICV4 + Nextera Flex	hCoV-19/Mozambique/MZ-L-IBV-97036011/2021	EPI_ISL_17075884		Male	15-30	Manhiça	Maputo	Mozambique	B.1.617.2	D614G_S(1.0)&L452R_S(1.0)&P681R_S(1.0)	98.49	1,2	None
Delta	COV036012	Instituto de Salud Global - ISGLOBAL (CAT_ISG)	2021-07-14	RNA			19.7				0.981	1056	Illumina MiSeq	ARTICV4 + Nextera Flex	hCoV-19/Mozambique/MZ-L-IBV-97036012/2021	EPI_ISL_17075885		Male	15-30	Manhiça	Maputo	Mozambique	B.1.617.2	D614G_S(1.0)&L452R_S(1.0)&P681R_S(1.0)	97.81	NA	NA
Delta	COV036013	Instituto de Salud Global - ISGLOBAL (CAT_ISG)	2021-07-14	RNA			25.5				0.9966	840	Illumina MiSeq	ARTICV4 + Nextera Flex	hCoV-19/Mozambique/MZ-L-IBV-97036013/2021	EPI_ISL_17075886		Male	>60	Manhiça	Maputo	Mozambique	B.1.617.2	A222V_S(1.0)&D614G_S(1.0)&L452R_S(1.0)&P681R_S(1.0)	98.62	NA	NA
Delta	COV036014	Instituto de Salud Global - ISGLOBAL (CAT_ISG)	2021-07-13	RNA			25				0.9966	1000	Illumina MiSeq	ARTICV4 + Nextera Flex	hCoV-19/Mozambique/MZ-L-IBV-97036014/2021	EPI_ISL_17075887		Female	31-45	Manhiça	Maputo	Mozambique	AY.6	D614G_S(1.0)&L452R_S(1.0)&P681R_S(0.99)	98.45	1,3,5	HIV-SIDA
Delta	COV036016	Instituto de Salud Global - ISGLOBAL (CAT_ISG)	2021-07-19	RNA			20				0.9799	800	Illumina MiSeq	ARTICV4 + Nextera Flex	hCoV-19/Mozambique/MZ-L-IBV-97036016/2021	EPI_ISL_17075888		Male	15-30	Manhiça	Maputo	Mozambique	B.1.617.2	D614G_S(1.0)&L452R_S(1.0)&P681R_S(1.0)	96.1	1,2	None
Delta	COV036017	Instituto de Salud Global - ISGLOBAL (CAT_ISG)	2021-07-13	RNA			24.9				0.9963	987	Illumina MiSeq	ARTICV4 + Nextera Flex	hCoV-19/Mozambique/MZ-L-IBV-97036017/2021	EPI_ISL_17075889		Male	46-60	Manhiça	Maputo	Mozambique	B.1.617.2	D614G_S(1.0)&L452R_S(1.0)&P681R_S(1.0)	99.07	1,3,5	HIV-SIDA
Delta	COV036018	Instituto de Salud Global - ISGLOBAL (CAT_ISG)	2021-08-13	RNA			21				0.945	569	Illumina MiSeq	ARTICV4 + Nextera Flex	hCoV-19/Mozambique/MZ-L-IBV-97036018/2021	EPI_ISL_17118745		Male	31-45	Manhiça	Maputo	Mozambique	AY.6	D614G_S(1.0)&L452R_S(1.0)&P681R_S(1.0)	90.14	2	HIV-SIDA
Delta	COV036019	Instituto de Salud Global - ISGLOBAL (CAT_ISG)	2021-08-16	RNA			18				0.9967	1225	Illumina MiSeq	ARTICV4 + Nextera Flex	hCoV-19/Mozambique/MZ-L-IBV-97036019/2021	EPI_ISL_17075890		Female	31-45	Manhiça	Maputo	Mozambique	B.1.617.2	D614G_S(1.0)&L452R_S(1.0)&P681R_S(1.0)	98.56	2,5	None
Delta	COV036020	Instituto de Salud Global - ISGLOBAL (CAT_ISG)	2021-07-19	RNA			24				0.9965	1115	Illumina MiSeq	ARTICV4 + Nextera Flex	hCoV-19/Mozambique/MZ-L-IBV-97036020/2021	EPI_ISL_17075891		Male	46-60	Manhiça	Maputo	Mozambique	B.1.617.2	D614G_S(1.0)&L452R_S(1.0)&P681R_S(1.0)	98.88	1,3,4,5	Hipertension
Delta	COV036021	Instituto de Salud Global - ISGLOBAL (CAT_ISG)	2021-08-11	RNA			22				0.9962	1216	Illumina MiSeq	ARTICV4 + Nextera Flex	hCoV-19/Mozambique/MZ-L-IBV-97036021/2021	EPI_ISL_17075892		Male	>60	Manhiça	Maputo	Mozambique	B.1.617.2	D614G_S(1.0)&L452R_S(1.0)&P681R_S(1.0)	98.78	3	Tuberculosis
Delta	COV036022	Instituto de Salud Global - ISGLOBAL (CAT_ISG)	2021-08-11	RNA			26				0.9648	882	Illumina MiSeq	ARTICV4 + Nextera Flex	hCoV-19/Mozambique/MZ-L-IBV-97036022/2021	EPI_ISL_17112720		Female	46-60	Manhiça	Maputo	Mozambique	B.1.617.2	D614G_S(1.0)&L452R_S(1.0)&P681R_S(1.0)	88.75	2,3,4,5	Hipertension
Delta	COV036024	Instituto de Salud Global - ISGLOBAL (CAT_ISG)	2021-07-22	RNA			26				0.9837	1277	Illumina MiSeq	ARTICV4 + Nextera Flex	hCoV-19/Mozambique/MZ-L-IBV-97036024/2021	EPI_ISL_17075893		Male	46-60	Manhiça	Maputo	Mozambique	B.1.617.2	D614G_S(1.0)&L452R_S(1.0)&P681R_S(1.0)	98.67	2,4,5	None
Delta	COV036025	Instituto de Salud Global - ISGLOBAL (CAT_ISG)	2021-07-22	RNA			15				0.9967	1384	Illumina MiSeq	ARTICV4 + Nextera Flex	hCoV-19/Mozambique/MZ-L-IBV-97036025/2021	EPI_ISL_17075894		Female	31-45	Manhiça	Maputo	Mozambique	AY.6	D614G_S(1.0)&L452R_S(1.0)&P681R_S(1.0)	98.74	3,5	None
Delta	COV036026	Instituto de Salud Global - ISGLOBAL (CAT_ISG)	2021-07-23	RNA			22				0.9808	1185	Illumina MiSeq	ARTICV4 + Nextera Flex	hCoV-19/Mozambique/MZ-L-IBV-97036026/2021	EPI_ISL_17075895		Female	<15	Manhiça	Maputo	Mozambique	B.1.617.2	D614G_S(1.0)&L452R_S(1.0)&P681R_S(1.0)	98.67	3	None
Delta	COV036027	Instituto de Salud Global - ISGLOBAL (CAT_ISG)	2021-07-23	RNA			26				0.9893	1310	Illumina MiSeq	ARTICV4 + Nextera Flex	hCoV-19/Mozambique/MZ-L-IBV-97036027/2021	EPI_ISL_17075896		Female	<15	Manhiça	Maputo	Mozambique	B.1.617.2	D614G_S(1.0)&L452R_S(1.0)&P681R_S(1.0)	98.74	1,3	None
Delta	COV036028	Instituto de Salud Global - ISGLOBAL (CAT_ISG)	2021-07-12	RNA			24				0.9792	1217	Illumina MiSeq	ARTICV4 + Nextera Flex	hCoV-19/Mozambique/MZ-L-IBV-97036028/2021	EPI_ISL_17075897		Female	31-45	Manhiça	Maputo	Mozambique	B.1.617.2	D614G_S(1.0)&L452R_S(1.0)&P681R_S(1.0)	98.76	1,2	None
Delta	COV036029	Instituto de Salud Global - ISGLOBAL (CAT_ISG)	2021-06-30	RNA			6.31				0.9967	1158	Illumina MiSeq	ARTICV4 + Nextera Flex	hCoV-19/Mozambique/MZ-L-IBV-97036029/2021	EPI_ISL_17075898		Female	31-45	Manhiça	Maputo	Mozambique	B.1.617.2	D614G_S(1.0)&L452R_S(1.0)&P681R_S(1.0)	98.67	1,3	HIV-SIDA
Delta	COV036030	Instituto de Salud Global - ISGLOBAL (CAT_ISG)	2021-07-31	RNA			16.4				0.9967	1215	Illumina MiSeq	ARTICV4 + Nextera Flex	hCoV-19/Mozambique/MZ-L-IBV-97036030/2021	EPI_ISL_17075899		Male	31-45	Manhiça	Maputo	Mozambique	B.1.617.2	D614G_S(1.0)&L452R_S(1.0)&P681R_S(1.0)	98.67	None	None
Delta	COV036031	Instituto de Salud Global - ISGLOBAL (CAT_ISG)	2021-07-12	RNA			19				0.988	1238	Illumina MiSeq	ARTICV4 + Nextera Flex	hCoV-19/Mozambique/MZ-L-IBV-97036031/2021	EPI_ISL_17075900		Male	15-30	Manhiça	Maputo	Mozambique	AY.6	D614G_S(1.0)&L452R_S(1.0)&P681R_S(1.0)	98.67	1,3,4,5	None
Delta	COV036032	Instituto de Salud Global - ISGLOBAL (CAT_ISG)	2021-07-22	RNA			20				0.9965	1232	Illumina MiSeq	ARTICV4 + Nextera Flex	hCoV-19/Mozambique/MZ-L-IBV-97036032/2021	EPI_ISL_17075901		Male	31-45	Manhiça	Maputo	Mozambique	AY.6	D614G_S(1.0)&L452R_S(1.0)&P681R_S(1.0)	98.64	1,3,4,5	None
Delta	COV036033	Instituto de Salud Global - ISGLOBAL (CAT_ISG)	2021-07-22	RNA			12				0.9951	1344	Illumina MiSeq	ARTICV4 + Nextera Flex	hCoV-19/Mozambique/MZ-L-IBV-97036033/2021	EPI_ISL_17075902		Female	46-60	Manhiça	Maputo	Mozambique	B.1.617.2	D614G_S(1.0)&L452R_S(1.0)&P681R_S(1.0)	98.73	1,2,3,5	HIV-SIDA
Delta	COV036034	Instituto de Salud Global - ISGLOBAL (CAT_ISG)	2021-08-11	RNA			24				0.9967	1685	Illumina MiSeq	ARTICV4 + Nextera Flex	hCoV-19/Mozambique/MZ-L-IBV-97036034/2021	EPI_ISL_17075903		Female	46-60	Manhiça	Maputo	Mozambique	B.1.617.2	D614G_S(1.0)&L452R_S(1.0)&P681R_S(1.0)	98.7	1,3	HIV-SIDA
Delta	COV036035	Instituto de Salud Global - ISGLOBAL (CAT_ISG)	2021-08-11	RNA			20				0.9967	1862	Illumina MiSeq	ARTICV4 + Nextera Flex	hCoV-19/Mozambique/MZ-L-IBV-97036035/2021	EPI_ISL_17075904		Male	>60	Manhiça	Maputo	Mozambique	B.1.617.2	D614G_S(1.0)&L452R_S(1.0)&P681R_S(1.0)&V1176F_S(1.0)	98.69	1,2	Hipertension
Delta	COV036036	Instituto de Salud Global - ISGLOBAL (CAT_ISG)	2021-06-21	RNA			18.52				0.9944	1055	Illumina MiSeq	ARTICV4 + Nextera Flex	hCoV-19/Mozambique/MZ-L-IBV-97036036/2021	EPI_ISL_17075905		Female	<15	Manhiça	Maputo	Mozambique	B.1.617.2	D614G_S(1.0)&L452R_S(1.0)&P681R_S(1.0)	98.18	3	Asthma
Delta	COV036037	Instituto de Salud Global - ISGLOBAL (CAT_ISG)	2021-08-12	RNA			20				0.9963	1374	Illumina MiSeq	ARTICV4 + Nextera Flex	hCoV-19/Mozambique/MZ-L-IBV-97036037/2021	EPI_ISL_17075906		Male	31-45	Manhiça	Maputo	Mozambique	B.1.617.2	D614G_S(1.0)&L452R_S(1.0)&P681R_S(1.0)	98.78	3	HIV-SIDA
Delta	COV036040	Instituto de Salud Global - ISGLOBAL (CAT_ISG)	2021-07-30	RNA			21.5				0.9963	2027	Illumina MiSeq	ARTICV4 + Nextera Flex	hCoV-19/Mozambique/MZ-L-IBV-97036040/2021	EPI_ISL_17118746		Male	46-60	Manhiça	Maputo	Mozambique	B.1.617.2	D614G_S(1.0)&L452R_S(1.0)&P681R_S(1.0)	98.69	2,5	None
Delta	COV036041	Instituto de Salud Global - ISGLOBAL (CAT_ISG)	2021-07-30	RNA			22.3				0.9966	1493	Illumina MiSeq	ARTICV4 + Nextera Flex	hCoV-19/Mozambique/MZ-L-IBV-97036041/2021	EPI_ISL_17118747		Female	>60	Manhiça	Maputo	Mozambique	B.1.617.2	D614G_S(1.0)&L452R_S(1.0)&P681R_S(1.0)	98.66	1,3,5	None
Delta	COV036042	Instituto de Salud Global - ISGLOBAL (CAT_ISG)	2021-07-30	RNA			17.4				0.9966	1318	Illumina MiSeq	ARTICV4 + Nextera Flex	hCoV-19/Mozambique/MZ-L-IBV-97036042/2021	EPI_ISL_17118748		Male	<15	Manhiça	Maputo	Mozambique	B.1.617.2	D614G_S(1.0)&L452R_S(1.0)&P681R_S(1.0)	98.61	1,2,4	None
Delta	COV036043	Instituto de Salud Global - ISGLOBAL (CAT_ISG)	2021-07-30	RNA			20.9				0.9967	1505	Illumina MiSeq	ARTICV4 + Nextera Flex	hCoV-19/Mozambique/MZ-L-IBV-97036043/2021	EPI_ISL_17075907		Female	15-30	Manhiça	Maputo	Mozambique	B.1.617.2	D614G_S(1.0)&L452R_S(1.0)&P681R_S(1.0)	98.78	1,3,4	None
Delta	COV036044	Instituto de Salud Global - ISGLOBAL (CAT_ISG)	2021-07-30	RNA			18.22				0.9885	1443	Illumina MiSeq	ARTICV4 + Nextera Flex	hCoV-19/Mozambique/MZ-L-IBV-97036044/2021	EPI_ISL_17075908		Female	31-45	Manhiça	Maputo	Mozambique	B.1.617.2	D614G_S(1.0)&L452R_S(1.0)&P681R_S(1.0)	98.71	2	None
Delta	COV036045	Instituto de Salud Global - ISGLOBAL (CAT_ISG)	2021-07-27	RNA			18.6				0.9913	1502	Illumina MiSeq	ARTICV4 + Nextera Flex	hCoV-19/Mozambique/MZ-L-IBV-97036045/2021	EPI_ISL_17075909		Female	46-60	Manhiça	Maputo	Mozambique	B.1.617.2	D614G_S(1.0)&L452R_S(1.0)&P681R_S(1.0)	98.58	3,5	None
Delta	COV036046	Instituto de Salud Global - ISGLOBAL (CAT_ISG)	2021-07-19	RNA			22				0.9967	1540	Illumina MiSeq	ARTICV4 + Nextera Flex	hCoV-19/Mozambique/MZ-L-IBV-97036046/2021	EPI_ISL_17075910		Female	31-45	Manhiça	Maputo	Mozambique	AY.29	D614G_S(1.0)&L452R_S(1.0)&P681R_S(1.0)	98.64	3,5	None
Delta	COV036047	Instituto de Salud Global - ISGLOBAL (CAT_ISG)	2021-06-21	RNA			3.61				0.9968	1463	Illumina MiSeq	ARTICV4 + Nextera Flex	hCoV-19/Mozambique/MZ-L-IBV-97036047/2021	EPI_ISL_17075911		Male	31-45	Manhiça	Maputo	Mozambique	B.1.617.2	D614G_S(1.0)&L452R_S(1.0)&P681R_S(1.0)	98.67	5	None
Delta	COV036049	Instituto de Salud Global - ISGLOBAL (CAT_ISG)	2021-07-31	RNA			23.3				0.9964	2060	Illumina MiSeq	ARTICV4 + Nextera Flex	hCoV-19/Mozambique/MZ-L-IBV-97036049/2021	EPI_ISL_17075912		Male	31-45	Manhiça	Maputo	Mozambique	B.1.617.2	D614G_S(1.0)&L452R_S(1.0)&P681R_S(1.0)	98.66	1,3,5	None
Delta	COV036050	Instituto de Salud Global - ISGLOBAL (CAT_ISG)	2021-07-31	RNA			18.6				0.9963	1258	Illumina MiSeq	ARTICV4 + Nextera Flex	hCoV-19/Mozambique/MZ-L-IBV-97036050/2021	EPI_ISL_17075913		Male	15-30	Manhiça	Maputo	Mozambique	B.1.617.2	D614G_S(1.0)&L452R_S(1.0)&P681R_S(1.0)	98.73	1,5	None
Delta	COV036051	Instituto de Salud Global - ISGLOBAL (CAT_ISG)	2021-07-31	RNA			14				0.9791	1551	Illumina MiSeq	ARTICV4 + Nextera Flex	hCoV-19/Mozambique/MZ-L-IBV-97036051/2021	EPI_ISL_17075914		Female	<15	Manhiça	Maputo	Mozambique	B.1.617.2	D614G_S(1.0)&L452R_S(1.0)&P681R_S(1.0)	98.66	1,3,5	None
Delta	COV036052	Instituto de Salud Global - ISGLOBAL (CAT_ISG)	2021-07-31	RNA			16.4				0.9793	1690	Illumina MiSeq	ARTICV4 + Nextera Flex	hCoV-19/Mozambique/MZ-L-IBV-97036052/2021	EPI_ISL_17075915		Male	15-30	Manhiça	Maputo	Mozambique	B.1.617.2	D614G_S(1.0)&L452R_S(1.0)&P681R_S(1.0)	98.62	1,2,5	None
Delta	COV036053	Instituto de Salud Global - ISGLOBAL (CAT_ISG)	2021-07-30	RNA			21				0.9911	1458	Illumina MiSeq	ARTICV4 + Nextera Flex	hCoV-19/Mozambique/MZ-L-IBV-97036053/2021	EPI_ISL_17075916		Male	31-45	Manhiça	Maputo	Mozambique	AY.6	D614G_S(1.0)&L452R_S(1.0)&P681R_S(1.0)	98.75	1,3,5	None
Delta	COV036055	Instituto de Salud Global - ISGLOBAL (CAT_ISG)	2021-07-12	RNA			25.6				0.9966	1601	Illumina MiSeq	ARTICV4 + Nextera Flex	hCoV-19/Mozambique/MZ-L-IBV-97036055/2021	EPI_ISL_17075917		Female	<15	Manhiça	Maputo	Mozambique	B.1.617.2	D614G_S(1.0)&L452R_S(1.0)&P681R_S(0.96)	98.44	3,4	None
Delta	COV036056	Instituto de Salud Global - ISGLOBAL (CAT_ISG)	2021-07-29	RNA			26				0.991	1527	Illumina MiSeq	ARTICV4 + Nextera Flex	hCoV-19/Mozambique/MZ-L-IBV-97036056/2021	EPI_ISL_17075918		Female	<15	Manhiça	Maputo	Mozambique	AY.6	D614G_S(1.0)&L452R_S(1.0)&P681R_S(1.0)	98.61	3	None
Delta	COV036057	Instituto de Salud Global - ISGLOBAL (CAT_ISG)	2021-06-30	RNA			8.51				0.9967	1497	Illumina MiSeq	ARTICV4 + Nextera Flex	hCoV-19/Mozambique/MZ-L-IBV-97036057/2021	EPI_ISL_17075919		Male	15-30	Manhiça	Maputo	Mozambique	B.1.617.2	D614G_S(1.0)&L452R_S(1.0)&P681R_S(1.0)	98.81	None	None
Delta	COV036058	Instituto de Salud Global - ISGLOBAL (CAT_ISG)	2021-06-30	RNA			7.12				0.9968	1650	Illumina MiSeq	ARTICV4 + Nextera Flex	hCoV-19/Mozambique/MZ-L-IBV-97036058/2021	EPI_ISL_17075920		Male	15-30	Manhiça	Maputo	Mozambique	AY.6	D614G_S(1.0)&L452R_S(1.0)&P681R_S(1.0)	98.7	2	None
Delta	COV036059	Instituto de Salud Global - ISGLOBAL (CAT_ISG)	2021-06-30	RNA			6.36				0.9963	1564	Illumina MiSeq	ARTICV4 + Nextera Flex	hCoV-19/Mozambique/MZ-L-IBV-97036059/2021	EPI_ISL_17075921		Female	15-30	Manhiça	Maputo	Mozambique	AY.6	D614G_S(1.0)&L452R_S(1.0)&P681R_S(1.0)	98.76	1,3,4,5	None
Delta	COV036061	Instituto de Salud Global - ISGLOBAL (CAT_ISG)	2021-06-30	RNA			12.79				0.9963	1685	Illumina MiSeq	ARTICV4 + Nextera Flex	hCoV-19/Mozambique/MZ-L-IBV-97036061/2021	EPI_ISL_17075922		Female	46-60	Manhiça	Maputo	Mozambique	B.1.617.2	D614G_S(1.0)&L452R_S(1.0)&P681R_S(0.99)	98.68	5	HIV-SIDA
Delta	COV036064	Instituto de Salud Global - ISGLOBAL (CAT_ISG)	2021-06-30	RNA			9.1				0.9968	1622	Illumina MiSeq	ARTICV4 + Nextera Flex	hCoV-19/Mozambique/MZ-L-IBV-97036064/2021	EPI_ISL_17075923		Female	<15	Manhiça	Maputo	Mozambique	B.1.617.2	D614G_S(1.0)&L452R_S(1.0)&P681R_S(1.0)	98.56	2	None
Delta	COV036067	Instituto de Salud Global - ISGLOBAL (CAT_ISG)	2021-06-30	RNA			20.05				0.9657	1125	Illumina MiSeq	ARTICV4 + Nextera Flex	hCoV-19/Mozambique/MZ-L-IBV-97036067/2021	EPI_ISL_17075924		Female	15-30	Manhiça	Maputo	Mozambique	B.1.617.2	D614G_S(1.0)&L452R_S(1.0)&P681R_S(1.0)	89.76	3	None
Delta	COV036069	Instituto de Salud Global - ISGLOBAL (CAT_ISG)	2021-06-30	RNA			3.84				0.9978	1485	Illumina MiSeq	ARTICV4 + Nextera Flex	hCoV-19/Mozambique/MZ-L-IBV-97036069/2021	EPI_ISL_17075925		Male	46-60	Manhiça	Maputo	Mozambique	AY.6	D614G_S(1.0)&L452R_S(1.0)&P681R_S(1.0)	98.48	1,2	None
Delta	COV036070	Instituto de Salud Global - ISGLOBAL (CAT_ISG)	2021-06-30	RNA			20.64				0.9963	2108	Illumina MiSeq	ARTICV4 + Nextera Flex	hCoV-19/Mozambique/MZ-L-IBV-97036070/2021	EPI_ISL_17075926		Female	15-30	Manhiça	Maputo	Mozambique	AY.29	D614G_S(1.0)&L452R_S(1.0)&P681R_S(1.0)	98.49	None	None
Delta	COV036071	Instituto de Salud Global - ISGLOBAL (CAT_ISG)	2021-06-30	RNA			8.78				0.9968	1517	Illumina MiSeq	ARTICV4 + Nextera Flex	hCoV-19/Mozambique/MZ-L-IBV-97036071/2021	EPI_ISL_17075927		Male	<15	Manhiça	Maputo	Mozambique	B.1.617.2	D614G_S(1.0)&L452R_S(1.0)&P681R_S(1.0)	98.4	NA	NA
Delta	COV036073	Instituto de Salud Global - ISGLOBAL (CAT_ISG)	2021-07-05	RNA			11.13				0.9967	1576	Illumina MiSeq	ARTICV4 + Nextera Flex	hCoV-19/Mozambique/MZ-L-IBV-97036073/2021	EPI_ISL_17075928		Male	<15	Manhiça	Maputo	Mozambique	B.1.617.2	D614G_S(1.0)&L452R_S(1.0)&P681R_S(1.0)	98.6	1,3	None
Delta	COV036074	Instituto de Salud Global - ISGLOBAL (CAT_ISG)	2021-06-15	RNA			19.28				0.9904	1481	Illumina MiSeq	ARTICV4 + Nextera Flex	hCoV-19/Mozambique/MZ-L-IBV-97036074/2021	EPI_ISL_17075929		Male	31-45	Manhiça	Maputo	Mozambique	B.1.617.2	D614G_S(1.0)&L452R_S(1.0)&P681R_S(1.0)	97.78	None	None
Delta	COV036075	Instituto de Salud Global - ISGLOBAL (CAT_ISG)	2021-07-12	RNA			20				0.9966	1603	Illumina MiSeq	ARTICV4 + Nextera Flex	hCoV-19/Mozambique/MZ-L-IBV-97036075/2021	EPI_ISL_17075930		Female	15-30	Manhiça	Maputo	Mozambique	B.1.617.2	D614G_S(1.0)&L452R_S(1.0)&P681R_S(1.0)	98.65	1,2	None
Delta	COV036076	Instituto de Salud Global - ISGLOBAL (CAT_ISG)	2021-07-12	RNA			23				0.9967	1319	Illumina MiSeq	ARTICV4 + Nextera Flex	hCoV-19/Mozambique/MZ-L-IBV-97036076/2021	EPI_ISL_17075931		Female	46-60	Manhiça	Maputo	Mozambique	B.1.617.2	D614G_S(1.0)&L452R_S(1.0)&P681R_S(1.0)	98.61	3,5	None
Delta	COV036078	Instituto de Salud Global - ISGLOBAL (CAT_ISG)	2021-07-05	RNA			6.67				0.9885	1376	Illumina MiSeq	ARTICV4 + Nextera Flex	hCoV-19/Mozambique/MZ-L-IBV-97036078/2021	EPI_ISL_17075932		Male	31-45	Manhiça	Maputo	Mozambique	B.1.617.2	D614G_S(1.0)&L452R_S(1.0)&P681R_S(1.0)	98.46	NA	NA
Delta	COV036079	Instituto de Salud Global - ISGLOBAL (CAT_ISG)	2021-06-28	RNA			9.18				0.9915	1270	Illumina MiSeq	ARTICV4 + Nextera Flex	hCoV-19/Mozambique/MZ-L-IBV-97036079/2021	EPI_ISL_17075933		Female	15-30	Manhiça	Maputo	Mozambique	B.1.617.2	D614G_S(1.0)&L452R_S(1.0)&P681R_S(1.0)	88.45	4,5	None
Delta	COV036080	Instituto de Salud Global - ISGLOBAL (CAT_ISG)	2021-07-15	RNA			22				0.9967	1472	Illumina MiSeq	ARTICV4 + Nextera Flex	hCoV-19/Mozambique/MZ-L-IBV-97036080/2021	EPI_ISL_17075934		Male	15-30	Manhiça	Maputo	Mozambique	B.1.617.2	D614G_S(1.0)&L452R_S(1.0)&P681R_S(1.0)	98.49	NA	NA
Delta	COV036081	Instituto de Salud Global - ISGLOBAL (CAT_ISG)	2021-07-22	RNA			24				0.9967	1440	Illumina MiSeq	ARTICV4 + Nextera Flex	hCoV-19/Mozambique/MZ-L-IBV-97036081/2021	EPI_ISL_17075935		Female	<15	Manhiça	Maputo	Mozambique	B.1.617.2	D614G_S(1.0)&L452R_S(1.0)&P681R_S(1.0)	98.66	2	None
Delta	COV036082	Instituto de Salud Global - ISGLOBAL (CAT_ISG)	2021-07-13	RNA			18				0.9968	2051	Illumina MiSeq	ARTICV4 + Nextera Flex	hCoV-19/Mozambique/MZ-L-IBV-97036082/2021	EPI_ISL_17075936		Male	31-45	Manhiça	Maputo	Mozambique	B.1.617.2	D614G_S(1.0)&L452R_S(1.0)&P681R_S(1.0)	98.6	1,2,4	Asthma
Delta	COV036083	Instituto de Salud Global - ISGLOBAL (CAT_ISG)	2021-07-20	RNA			18				0.9967	1675	Illumina MiSeq	ARTICV4 + Nextera Flex	hCoV-19/Mozambique/MZ-L-IBV-97036083/2021	EPI_ISL_17075937		Male	15-30	Manhiça	Maputo	Mozambique	B.1.617.2	D614G_S(1.0)&L452R_S(1.0)&P681R_S(1.0)	98.59	3	Gastrite
Delta	COV036084	Instituto de Salud Global - ISGLOBAL (CAT_ISG)	2021-07-30	RNA			23.8				0.9966	1706	Illumina MiSeq	ARTICV4 + Nextera Flex	hCoV-19/Mozambique/MZ-L-IBV-97036084/2021	EPI_ISL_17075938		Female	15-30	Manhiça	Maputo	Mozambique	B.1.617.2	D614G_S(1.0)&L452R_S(1.0)&P681R_S(1.0)	98.6	2	None
Delta	COV036085	Instituto de Salud Global - ISGLOBAL (CAT_ISG)	2021-06-30	RNA			7.55				0.9967	1549	Illumina MiSeq	ARTICV4 + Nextera Flex	hCoV-19/Mozambique/MZ-L-IBV-97036085/2021	EPI_ISL_17075939		Female	46-60	Manhiça	Maputo	Mozambique	B.1.617.2	D614G_S(1.0)&L452R_S(1.0)&P681R_S(1.0)	98.75	3,5	HIV-SIDA
Delta	COV036088	Instituto de Salud Global - ISGLOBAL (CAT_ISG)	2021-06-17	RNA			8.47				0.9968	1679	Illumina MiSeq	ARTICV4 + Nextera Flex	hCoV-19/Mozambique/MZ-L-IBV-97036088/2021	EPI_ISL_17075940		Male	31-45	Manhiça	Maputo	Mozambique	AY.6	D614G_S(1.0)&L452R_S(1.0)&P681R_S(1.0)	98.6	1,2,5	Hipertension
Delta	COV036090	Instituto de Salud Global - ISGLOBAL (CAT_ISG)	2021-06-22	RNA			5.38				0.9968	1540	Illumina MiSeq	ARTICV4 + Nextera Flex	hCoV-19/Mozambique/MZ-L-IBV-97036090/2021	EPI_ISL_17075941		Female	46-60	Manhiça	Maputo	Mozambique	AY.6	D614G_S(1.0)&L452R_S(1.0)&P681R_S(1.0)	98.71	1,3	None
Delta	COV036091	Instituto de Salud Global - ISGLOBAL (CAT_ISG)	2021-07-12	RNA			21				0.9967	1621	Illumina MiSeq	ARTICV4 + Nextera Flex	hCoV-19/Mozambique/MZ-L-IBV-97036091/2021	EPI_ISL_17075942		Male	15-30	Manhiça	Maputo	Mozambique	AY.19	D614G_S(1.0)&L452R_S(1.0)&P681R_S(1.0)	98.6	1	None
Delta	COV036095	Instituto de Salud Global - ISGLOBAL (CAT_ISG)	2021-07-05	RNA			12.23				0.9967	1728	Illumina MiSeq	ARTICV4 + Nextera Flex	hCoV-19/Mozambique/MZ-L-IBV-97036095/2021	EPI_ISL_17075943		Male	31-45	Manhiça	Maputo	Mozambique	AY.6	D614G_S(1.0)&L452R_S(1.0)&P681R_S(1.0)	98.54	1,3,5	None
Delta	COV036097	Instituto de Salud Global - ISGLOBAL (CAT_ISG)	2021-08-31	RNA			26				0.9964	1395	Illumina MiSeq	ARTICV4 + Nextera Flex	hCoV-19/Mozambique/MZ-L-IBV-97036097/2021	EPI_ISL_17075944		Male	15-30	Manhiça	Maputo	Mozambique	B.1.617.2	D614G_S(1.0)&L452R_S(1.0)&P681R_S(1.0)	98.74	3	None
Delta	COV035965	Instituto de Salud Global - ISGLOBAL (CAT_ISG)	2021-08-09	RNA			16				0.9967	1424	Illumina MiSeq	ARTICV4 + Nextera Flex	hCoV-19/Mozambique/MZ-L-IBV-97035965/2021	EPI_ISL_17156366		Male	31-45	Manhiça	Maputo	Mozambique	B.1.617.2	D614G_S(1.0)&L452R_S(1.0)&P681R_S(1.0)	98.5	1,3,4,5	None
Delta	COV035929	Instituto de Salud Global - ISGLOBAL (CAT_ISG)	2021-08-12	RNA			16				0.9967	1294	Illumina MiSeq	ARTICV4 + Nextera Flex	hCoV-19/Mozambique/MZ-L-IBV-97035929/2021	EPI_ISL_17156367		Male	<15	Manhiça	Maputo	Mozambique	B.1.617.2	D614G_S(1.0)&L452R_S(1.0)&P681R_S(1.0)	98.47	2	Asthma
Delta	COV036100	Instituto de Salud Global - ISGLOBAL (CAT_ISG)	2021-07-05	RNA			4.56				0.9967	1394	Illumina MiSeq	ARTICV4 + Nextera Flex	hCoV-19/Mozambique/MZ-L-IBV-97036100/2021	EPI_ISL_17075947		Male	31-45	Manhiça	Maputo	Mozambique	B.1.617.2	D614G_S(1.0)&L452R_S(1.0)&P681R_S(1.0)	98.66	1,2,4	None
Delta	COV036101	Instituto de Salud Global - ISGLOBAL (CAT_ISG)	2021-07-05	RNA			12.48				0.9964	1214	Illumina MiSeq	ARTICV4 + Nextera Flex	hCoV-19/Mozambique/MZ-L-IBV-97036101/2021	EPI_ISL_17075948		Male	46-60	Manhiça	Maputo	Mozambique	B.1.617.2	D614G_S(1.0)&L452R_S(1.0)&P681R_S(1.0)	98.51	NA	NA
Delta	COV035967	Instituto de Salud Global - ISGLOBAL (CAT_ISG)	2021-08-10	RNA			22				0.9967	1260	Illumina MiSeq	ARTICV4 + Nextera Flex	hCoV-19/Mozambique/MZ-L-IBV-97035967/2021	EPI_ISL_17156368		Female	31-45	Manhiça	Maputo	Mozambique	B.1.617.2	D614G_S(1.0)&L452R_S(1.0)&P681R_S(0.99)	98.41	1,3	None
Delta	COV035966	Instituto de Salud Global - ISGLOBAL (CAT_ISG)	2021-08-09	RNA			18				0.9967	1256	Illumina MiSeq	ARTICV4 + Nextera Flex	hCoV-19/Mozambique/MZ-L-IBV-97035966/2021	EPI_ISL_17156369		Female	>60	Manhiça	Maputo	Mozambique	B.1.617.2	D614G_S(1.0)&L452R_S(1.0)&P681R_S(1.0)	98.52	1,5	Hipertension
Delta	COV036104	Instituto de Salud Global - ISGLOBAL (CAT_ISG)	2021-06-21	RNA			21.3				0.9967	1310	Illumina MiSeq	ARTICV4 + Nextera Flex	hCoV-19/Mozambique/MZ-L-IBV-97036104/2021	EPI_ISL_17075951		Female	<15	Manhiça	Maputo	Mozambique	B.1.617.2	D614G_S(1.0)&L452R_S(1.0)&P681R_S(1.0)	98.37	1,3	None
Delta	COV036109	Instituto de Salud Global - ISGLOBAL (CAT_ISG)	2021-07-27	RNA			26.6				0.9967	1532	Illumina MiSeq	ARTICV4 + Nextera Flex	hCoV-19/Mozambique/MZ-L-IBV-97036109/2021	EPI_ISL_17075952		Male	31-45	Manhiça	Maputo	Mozambique	B.1.617.2	D614G_S(1.0)&L452R_S(1.0)&P681R_S(1.0)	98.56	1,3	None
Delta	COV036110	Instituto de Salud Global - ISGLOBAL (CAT_ISG)	2021-07-27	RNA			27				0.9966	1195	Illumina MiSeq	ARTICV4 + Nextera Flex	hCoV-19/Mozambique/MZ-L-IBV-97036110/2021	EPI_ISL_17075953		Male	31-45	Manhiça	Maputo	Mozambique	B.1.617.2	D614G_S(1.0)&L452R_S(1.0)&P681R_S(1.0)	98.62	2,5	None

Delta	COV036111	Instituto de Salud Global - ISGLOBAL (CAT_ISG)	2021-07-13	RNA			27				0.9967	1015	Illumina MiSeq	ARTICV4 + Nextera Flex	hCoV-19/Mozambique/MZ-L-IBV-97036111/2021	EPI_ISL_17075954		Female	31-45	Manhiça	Maputo	Mozambique	AY.6	D614G_S(1.0)&L452R_S(1.0)&P681R_S(1.0)	98.78	1,3,5	None
Delta	COV036112	Instituto de Salud Global - ISGLOBAL (CAT_ISG)	2021-07-17	RNA			27				0.9813	1266	Illumina MiSeq	ARTICV4 + Nextera Flex	hCoV-19/Mozambique/MZ-L-IBV-97036112/2021	EPI_ISL_17075955		Male	<15	Manhiça	Maputo	Mozambique	AY.6	D614G_S(1.0)&L452R_S(1.0)&P681R_S(1.0)	97.4	NA	NA
Delta	COV036113	Instituto de Salud Global - ISGLOBAL (CAT_ISG)	2021-07-01	RNA			27.42				0.9839	1224	Illumina MiSeq	ARTICV4 + Nextera Flex	hCoV-19/Mozambique/MZ-L-IBV-97036113/2021	EPI_ISL_17075956		Male	<15	Manhiça	Maputo	Mozambique	B.1.617.2	D614G_S(1.0)&L452R_S(1.0)&P681R_S(1.0)	97.85	1	None
other	COV025325	Instituto de Salud Global - ISGLOBAL (CAT_ISG)	2021-02-12	RNA			18			6	0.9079	933	Illumina MiSeq	ARTICV3 + Nextera Flex	hCoV-19/Mozambique/MZ-L-IBV-97025325/2021	EPI_ISL_2442102	ERR6018091	Male	31-45	Manhiça	Maputo	Mozambique	B.1.1.375	D614G_S(1.0)	82.83	NA	NA
other	COV025328	Instituto de Salud Global - ISGLOBAL (CAT_ISG)	2021-02-12	RNA			15			15.2	0.9574	1194	Illumina MiSeq	ARTICV3 + Nextera Flex	hCoV-19/Mozambique/MZ-L-IBV-97025328/2021	EPI_ISL_2442105	ERR6018094	Male	31-45	Manhiça	Maputo	Mozambique	B.1.1.375	D614G_S(0.99)	93.07	NA	NA
other	COV025363	Instituto de Salud Global - ISGLOBAL (CAT_ISG)	2021-01-25	RNA			6			236	0.9966	1324	Illumina MiSeq	ARTICV3 + Nextera Flex	hCoV-19/Mozambique/MZ-L-IBV-97025363/2021	EPI_ISL_2442121	ERR6018125	Male	15-30	Manhiça	Maputo	Mozambique	B.1.1.375	D614G_S(1.0)	99.19	NA	NA
other	COV025379	Instituto de Salud Global - ISGLOBAL (CAT_ISG)	2021-01-25	RNA		17.83	7			92.5	0.9811	1773	Illumina MiSeq	ARTICV3 + Nextera Flex	hCoV-19/Mozambique/MZ-L-IBV-97025379/2021	EPI_ISL_2442134	ERR6018146	Female	15-30	Manhiça	Maputo	Mozambique	B.1.1.375	D614G_S(1.0)	98.67	NA	NA
other	COV025404	Instituto de Salud Global - ISGLOBAL (CAT_ISG)	2020-12-31	RNA		11.97	18			188	0.9813	1429	Illumina MiSeq	ARTICV3 + Nextera Flex	hCoV-19/Mozambique/MZ-L-IBV-97025404/2020	EPI_ISL_2442151	ERR6018177	Male	15-30	Manhiça	Maputo	Mozambique	B.1.1.375	D614G_S(1.0)	98.68	NA	NA
other	COV025406	Instituto de Salud Global - ISGLOBAL (CAT_ISG)	2020-12-30	RNA		16.26	21			146	0.9812	1477	Illumina MiSeq	ARTICV3 + Nextera Flex	hCoV-19/Mozambique/MZ-L-IBV-97025406/2020	EPI_ISL_2442153	ERR6018182	Male	15-30	Manhiça	Maputo	Mozambique	B.1.1.375	D614G_S(1.0)	98.77	NA	NA
other	COV025407	Instituto de Salud Global - ISGLOBAL (CAT_ISG)	2021-01-05	RNA		17.14	22			114	0.9813	1732	Illumina MiSeq	ARTICV3 + Nextera Flex	hCoV-19/Mozambique/MZ-L-IBV-97025407/2021	EPI_ISL_2442154	ERR6018187	Male	46-60	Manhiça	Maputo	Mozambique	B.1.1.375	D614G_S(1.0)	98.53	NA	NA
other	COV025435	Instituto de Salud Global - ISGLOBAL (CAT_ISG)	2020-11-03	RNA		23.88	24			68	0.9889	1298	Illumina MiSeq	ARTICV3 + Nextera Flex	hCoV-19/Mozambique/MZ-L-IBV-97025435/2020	EPI_ISL_2442180	ERR6018269	Female		Manhiça	Maputo	Mozambique	B.1.1.54	D614G_S(1.0)	98.69	NA	NA
other	COV025436	Instituto de Salud Global - ISGLOBAL (CAT_ISG)	2020-11-04	RNA		23.7	24			67.5	0.9868	1238	Illumina MiSeq	ARTICV3 + Nextera Flex	hCoV-19/Mozambique/MZ-L-IBV-97025436/2020	EPI_ISL_2442181	ERR6018273	Male	31-45	Manhiça	Maputo	Mozambique	B.1	D614G_S(1.0)	98.17	NA	NA
other	COV025437	Instituto de Salud Global - ISGLOBAL (CAT_ISG)	2020-12-02	RNA		18.01	24			151	0.9899	1599	Illumina MiSeq	ARTICV3 + Nextera Flex	hCoV-19/Mozambique/MZ-L-IBV-97025437/2020	EPI_ISL_2442182	ERR6018275	Male	>60	Manhiça	Maputo	Mozambique	C.1.1	D614G_S(1.0)	98.59	NA	NA
other	COV025438	Instituto de Salud Global - ISGLOBAL (CAT_ISG)	2020-11-23	RNA		16.37	17			169	0.9836	1577	Illumina MiSeq	ARTICV3 + Nextera Flex	hCoV-19/Mozambique/MZ-L-IBV-97025438/2020	EPI_ISL_2442183	ERR6018277	Male	15-30	Manhiça	Maputo	Mozambique	B.1.1.375	D614G_S(1.0)	98.71	NA	NA
other	COV025439	Instituto de Salud Global - ISGLOBAL (CAT_ISG)	2020-11-20	RNA		30.29	23			7.5	0.9498	1205	Illumina MiSeq	ARTICV3 + Nextera Flex	hCoV-19/Mozambique/MZ-L-IBV-97025439/2020	EPI_ISL_2442184	ERR6018278	Female	46-60	Manhiça	Maputo	Mozambique	B.1.1.375	D614G_S(1.0)	90.99	NA	NA
other	COV025441	Instituto de Salud Global - ISGLOBAL (CAT_ISG)	2020-11-05	RNA		19.37	8			131.5	0.9772	1446	Illumina MiSeq	ARTICV3 + Nextera Flex	hCoV-19/Mozambique/MZ-L-IBV-97025441/2020	EPI_ISL_2442186	ERR6018288	Female	15-30	Manhiça	Maputo	Mozambique	B.1.1	D614G_S(1.0)	98.61	NA	NA
other	COV025442	Instituto de Salud Global - ISGLOBAL (CAT_ISG)	2020-11-09	RNA		33.35	18			1	0.9021	597	Illumina MiSeq	ARTICV3 + Nextera Flex	hCoV-19/Mozambique/MZ-L-IBV-97025442/2020	EPI_ISL_2442187	ERR6018291	Male	31-45	Manhiça	Maputo	Mozambique	C.6	D614G_S(1.0)	56.35	NA	NA
other	COV025443	Instituto de Salud Global - ISGLOBAL (CAT_ISG)	2020-11-04	RNA		31.36	24			3.2	0.9246	922	Illumina MiSeq	ARTICV3 + Nextera Flex	hCoV-19/Mozambique/MZ-L-IBV-97025443/2020	EPI_ISL_2442188	ERR6018292	Male	>60	Manhiça	Maputo	Mozambique	B.1.1.1	D614G_S(1.0)	67.14	NA	NA
other	COV025444	Instituto de Salud Global - ISGLOBAL (CAT_ISG)	2020-12-02	RNA		17.7	21			144	0.9827	1397	Illumina MiSeq	ARTICV3 + Nextera Flex	hCoV-19/Mozambique/MZ-L-IBV-97025444/2020	EPI_ISL_2442189	ERR6018297	Female	15-30	Manhiça	Maputo	Mozambique	B.1.1.375	D614G_S(1.0)	98.71	NA	NA
other	COV025447	Instituto de Salud Global - ISGLOBAL (CAT_ISG)	2020-11-03	RNA		19.18	20			133.5	0.9752	1362	Illumina MiSeq	ARTICV3 + Nextera Flex	hCoV-19/Mozambique/MZ-L-IBV-97025447/2020	EPI_ISL_2442190	ERR6018301	Female	31-45	Manhiça	Maputo	Mozambique	B.1.1.375	D614G_S(1.0)	97.84	NA	NA
other	COV025448	Instituto de Salud Global - ISGLOBAL (CAT_ISG)	2020-11-10	RNA		24.61	20			47.5	0.986	1638	Illumina MiSeq	ARTICV3 + Nextera Flex	hCoV-19/Mozambique/MZ-L-IBV-97025448/2020	EPI_ISL_3246532	ERR6018305	Male	46-60	Manhiça	Maputo	Mozambique	B.1.1.448	D614G_S(1.0)	96.71	NA	NA
other	COV025449	Instituto de Salud Global - ISGLOBAL (CAT_ISG)	2020-11-23	RNA		22.11	19			79.5	0.9795	1321	Illumina MiSeq	ARTICV3 + Nextera Flex	hCoV-19/Mozambique/MZ-L-IBV-97025449/2020	EPI_ISL_2442191	ERR6018306	Female	>60	Manhiça	Maputo	Mozambique	B.1.1.375	D614G_S(1.0)	98.39	NA	NA
other	COV025451	Instituto de Salud Global - ISGLOBAL (CAT_ISG)	2020-11-09	RNA		14.66	5			152.5	0.9845	1536	Illumina MiSeq	ARTICV3 + Nextera Flex	hCoV-19/Mozambique/MZ-L-IBV-97025451/2020	EPI_ISL_2442192	ERR6018308	Male	31-45	Manhiça	Maputo	Mozambique	B.1.1.375	D614G_S(1.0)	98.69	NA	NA
other	COV035941	Instituto de Salud Global - ISGLOBAL (CAT_ISG)	2021-06-28	RNA			5.52				0.9811	1929	Illumina MiSeq	ARTICV4 + Nextera Flex	hCoV-19/Mozambique/MZ-L-IBV-97035941/2021	EPI_ISL_17118742		Female	>60	Manhiça	Maputo	Mozambique	C.1.2	E484K_S(1.0)&N501Y_S(1.0)&D614G_S(1.0)&T716I_S(1.0)&H655Y_S(1.0)&R190S_S(1.0)	98.24	4	None
other	COV035996	Instituto de Salud Global - ISGLOBAL (CAT_ISG)	2021-07-03	RNA			10.16				0.9809	1128	Illumina MiSeq	ARTICV4 + Nextera Flex	hCoV-19/Mozambique/MZ-L-IBV-97035996/2021	EPI_ISL_17118744		Female	31-45	Manhiça	Maputo	Mozambique	C.1.2	E484K_S(1.0)&N501Y_S(1.0)&D614G_S(1.0)&T716I_S(1.0)&H655Y_S(1.0)&R190S_S(1.0)	98.45	NA	NA
other	COV036004	Instituto de Salud Global - ISGLOBAL (CAT_ISG)	2021-03-15	RNA			14.35				0.994	951	Illumina MiSeq	ARTICV4 + Nextera Flex	hCoV-19/Mozambique/MZ-L-IBV-97036004/2021	EPI_ISL_17075878		Female	>60	Manhiça	Maputo	Mozambique	B.1	L18F_S(0.74)&E484K_S(0.73)&N501Y_S(0.69)&D614G_S(1.0)&Mink_M1229I_S(0.79)&A701V_S(0.8)&K417N_S(0.89)&L452R_S(0.13)&P681R_S(0.23)	94.21	None	Hipertension
other	COV036060	Instituto de Salud Global - ISGLOBAL (CAT_ISG)	2021-06-30	RNA			9.15				0.9808	1836	Illumina MiSeq	ARTICV4 + Nextera Flex	hCoV-19/Mozambique/MZ-L-IBV-97036060/2021	EPI_ISL_17118749		Female	31-45	Manhiça	Maputo	Mozambique	C.1.2	E484K_S(1.0)&N501Y_S(1.0)&D614G_S(1.0)&T716I_S(1.0)&H655Y_S(1.0)&R190S_S(1.0)	98.72	3,4,5	None
other	COV036096	Instituto de Salud Global - ISGLOBAL (CAT_ISG)	2021-07-09	RNA			23				0.981	1860	Illumina MiSeq	ARTICV4 + Nextera Flex	hCoV-19/Mozambique/MZ-L-IBV-97036096/2021	EPI_ISL_17118750		Male		Manhiça	Maputo	Mozambique	C.1.2	E484K_S(1.0)&N501Y_S(1.0)&D614G_S(1.0)&T716I_S(1.0)&H655Y_S(1.0)&R190S_S(1.0)	98.41	1,3	None
																										1: Fever	
																										2: Dry cough	
																										3: Productive cough	
																										4. Breathing difficulties	
																										5: Loss of smell/taste	

Beta transmission groups

Group	samples_Mz	cluster_size	samples_NewIBV	%Mz	%Mz_New_IBV	Likely_geographical_origin	Likely_introduction_period	Samples_included
G01	2	2	2	100.0	100.0	71% SouthAfrica, 39 % Zimbabwe	2020-11-02 ; 2021-01-05	Mozambique/MZ-L-IBV-97025423/2021_EPI_ISL_2442169_2021-01-19, Mozambique/MZ-L-IBV-97025426/2021_EPI_ISL_2442172_2021-01-05
G02	2	2	2	100.0	100.0	SouthAfrica	2020-12-21 ; 2021-01-21	Mozambique/MZ-L-IBV-97025378/2021_EPI_ISL_2442133_2021-01-23, Mozambique/MZ-L-IBV-97025413/2021_EPI_ISL_2442159_2021-01-21
G03	2	2	2	100.0	100.0	SouthAfrica	2021-01-26 ; 2021-03-03	Mozambique/MZ-L-IBV-97025279/2021_EPI_ISL_3246530_2021-03-04, Mozambique/MZ-L-IBV-97025281/2021_EPI_ISL_2442087_2021-03-03
G04	2	2	0	100.0	0.0	SouthAfrica	2021-01-07 ; 2021-03-09	Mozambique/CERI-KRISP-K015022/2021_EPI_ISL_2617103_2021-03-11, Mozambique/CERI-KRISP-K015005/2021_EPI_ISL_2617102_2021-03-09
G05	2	2	0	100.0	0.0	England	2020-12-15 ; 2020-12-23	Mozambique/INS-K007911/2020_EPI_ISL_887439_2020-12-26, Mozambique/INS-K007936/2020_EPI_ISL_887446_2020-12-23
G06	2	2	0	100.0	0.0	SouthAfrica	2021-01-11 ; 2021-03-09	Mozambique/CERI-KRISP-K015047/2021_EPI_ISL_2617166_2021-03-17, Mozambique/CERI-KRISP-K015006/2021_EPI_ISL_2617111_2021-03-09
G07	2	2	0	100.0	0.0	SouthAfrica	2020-12-22 ; 2021-02-05	Mozambique/CERI-KRISP-K015060/2021_EPI_ISL_2617107_2021-03-17, Mozambique/INS-K011880/2021_EPI_ISL_2396985_2021-02-05
G08	2	2	0	100.0	0.0	Undetermined	before 2020-10-31	Mozambique/INS-K011825/2020_EPI_ISL_2396930_2020-11-01, Mozambique/INS-K011823/2020_EPI_ISL_2396928_2020-10-31
G09	2	2	2	100.0	100.0	75% SouthAfrica, 25% Canada	2020-12-23 ; 2021-02-24	Mozambique/MZ-L-IBV-97025327/2021_EPI_ISL_2442104_2021-02-24, Mozambique/MZ-L-IBV-97025299/2021_EPI_ISL_2442091_2021-03-04
G10	2	2	2	100.0	100.0	43% SouthAfrica, 43% Malawi; 14% Zimbabwe	2020-12-18 ; 2021-02-12	Mozambique/MZ-L-IBV-97025302/2021_EPI_ISL_2442092_2021-02-20, Mozambique/MZ-L-IBV-97025308/2021_EPI_ISL_2442096_2021-02-12
G11	2	2	2	100.0	100.0	SouthAfrica	2020-12-28 ; 2021-01-15	Mozambique/MZ-L-IBV-97025433/2021_EPI_ISL_2442178_2021-01-15, Mozambique/MZ-L-IBV-97025430/2021_EPI_ISL_2442176_2021-01-15
G12	2	2	0	100.0	0.0	SouthAfrica	2021-05-24 ; 2021-05-29	Mozambique/COV-2332-A1981/2021_EPI_ISL_5822556_2021-05-29, Mozambique/COV-2329-A1978/2021_EPI_ISL_5822554_2021-05-29
G13	2	2	2	100.0	100.0	SouthAfrica	2021-01-11 ; 2021-01-23	Mozambique/MZ-L-IBV-97025367/2021_EPI_ISL_2442125_2021-01-23, Mozambique/MZ-L-IBV-97025377/2021_EPI_ISL_2442132_2021-01-23
G14	2	2	0	100.0	0.0	62.5% UK, 37.5% SouthAfrica	2020-12-15 ; 2021-03-13	Mozambique/CERI-KRISP-K015063/2021_EPI_ISL_2617105_2021-03-18, Mozambique/CERI-KRISP-K015039/2021_EPI_ISL_2617112_2021-03-13
G15	2	2	0	100.0	0.0	SouthAfrica	2021-02-16 ; 2021-02-22	Mozambique/CERI-KRISP-K015033/2021_EPI_ISL_2617097_2021-03-12, Mozambique/INS-K011862/2021_EPI_ISL_2396967_2021-02-22
G16	2	2	2	100.0	100.0	SouthAfrica	2021-01-02 ; 2021-01-17	Mozambique/MZ-L-IBV-97025345/2021_EPI_ISL_2442109_2021-02-09, Mozambique/MZ-L-IBV-97025421/2021_EPI_ISL_2442167_2021-01-17
G17	2	2	0	100.0	0.0	50% SouthAfrica, 50% Denmark	2020-12-23 ; 2021-01-10	Mozambique/INS-K008163/2021_EPI_ISL_964940_2021-01-14, Mozambique/INS-K008775/2021_EPI_ISL_1132822_2021-01-10
G18	2	2	0	100.0	0.0	88% SouthAfrica, 12% Ireland	2020-12-14 ; 2021-03-10	Mozambique/COV-2354-A2003/2021_EPI_ISL_5822561_2021-05-22, Mozambique/CERI-KRISP-K015016/2021_EPI_ISL_2617184_2021-03-10
G19	2	2	0	100.0	0.0	Undetermined	Before 2021-04-20	Mozambique/COV-2293-A1941/2021_EPI_ISL_5822565_2021-05-31, Mozambique/CERI-KRISP-K016068/2021_EPI_ISL_3447998_2021-04-20
G20	3	3	1	100.0	33.3	SouthAfrica	2020-12-21 ; 2020-12-27	Mozambique/MZ-L-IBV-97025453/2021_EPI_ISL_2442194_2021-01-18, Mozambique/INS-K007918/2020_EPI_ISL_887440_2020-12-27, Mozambique/COV-2340-A1989/2021_EPI_ISL_5822557_2021-05-05
G21	3	3	0	100.0	0.0	Undetermined	before 2021-05-31	Mozambique/COV-2298-A1946/2021_EPI_ISL_5822566_2021-05-31, Mozambique/COV-2252-A1900/2021_EPI_ISL_5822552_2021-05-31, Mozambique/COV-2349-A1998/2021_EPI_ISL_5822560_2021-05-31
G22	2	3	0	66.7	0.0	SouthAfrica	2020-12-28 ; 2021-01-10	Portugal/PT2925/2021_EPI_ISL_1414437_2021-02-07, Mozambique/INS-K008773/2021_EPI_ISL_1132820_2021-01-10, Mozambique/INS-K011853/2021_EPI_ISL_2396958_2021-02-21
G23	3	3	0	100.0	0.0	Undetermined	Before 2021-05-10	Mozambique/COV-2334-A1983/2021_EPI_ISL_5822680_2021-05-10, Mozambique/COV-2346-A1995/2021_EPI_ISL_5822559_2021-05-29, Mozambique/COV-2330-A1979/2021_EPI_ISL_5822555_2021-05-29
G24	3	3	1	100.0	33.3	SouthAfrica	2021-01-05 ; 2021-01-13	Mozambique/INS-K008130/2021_EPI_ISL_964935_2021-01-16, Mozambique/CERI-KRISP-K015044/2021_EPI_ISL_2617114_2021-03-16, Mozambique/MZ-L-IBV-97025401/2021_EPI_ISL_2442148_2021-01-13
G25	2	3	0	66.7	0.0	Portugal	2021-03-04 ; 2021-03-17	Mozambique/CERI-KRISP-K015052/2021_EPI_ISL_2617116_2021-03-17, Mozambique/COV-2341-A1990/2021_EPI_ISL_5822674_2021-05-04, SouthAfrica/NICD-N8409/2021_EPI_ISL_2695754_2021-05-06
G26	2	3	0	66.7	0.0	SouthAfrica	2020-12-21 ; 2021-01-23	Mozambique/COV-2139-A1786/2021_EPI_ISL_5822549_2021-06-22, Mozambique/CERI-KRISP-K015998/2021_EPI_ISL_3447976_2021-03-31, India/TG-RFCH00858_CIB3170/2021_EPI_ISL_6877898_2021-01-23
G27	2	3	1	66.7	50.0	SouthAfrica	2020-12-21 ; 2021-01-28	Mozambique/MZ-L-IBV-97025280/2021_EPI_ISL_2442086_2021-03-03, Angola/KRISP-K009740/2021_EPI_ISL_1347913_2021-01-28, Mozambique/COV-2362-A2011/2021_EPI_ISL_5822562_2021-05-23
G28	3	3	0	100.0	0.0	Angola	2021-01-28 ; 2021-04-26	Mozambique/COV-2287-A1935/2021_EPI_ISL_5822695_2021-05-01, Mozambique/COV-2326-A1975/2021_EPI_ISL_5822553_2021-04-26, Mozambique/COV-2301-A1949/2021_EPI_ISL_5822567_2021-04-26
G29	3	3	0	100.0	0.0	SouthAfrica	2020-11-19 ; 2021-01-08	Mozambique/INS-K008157/2021_EPI_ISL_964933_2021-01-14, Mozambique/INS-K008145/2021_EPI_ISL_964930_2021-01-15, Mozambique/INS-K008126/2021_EPI_ISL_964924_2021-01-08
G30	3	4	0	75.0	0.0	SouthAfrica	2020-12-21 ; 2021-02-24	Mozambique/CERI-KRISP-K015064/2021_EPI_ISL_2617104_2021-03-18, Mozambique/CERI-KRISP-K015032/2021_EPI_ISL_2617121_2021-03-12, SouthAfrica/UP-K012897-KRISP/2021_EPI_ISL_2727289_2021-02-24, hCoV-19/Mozambique/INS-K011886/2021_2021-02-08_2021-06-03
G31	4	4	0	100.0	0.0	SouthAfrica	2021-02-16 ; 2021-03-31	Mozambique/COV-2376-A2025/2021_EPI_ISL_5822677_2021-03-31, Mozambique/CERI-KRISP-K016049/2021_EPI_ISL_3447996_2021-04-16, Mozambique/CERI-KRISP-K016082/2021_EPI_ISL_3447977_2021-04-20, Mozambique/CERI-KRISP-K016059/2021_EPI_ISL_3447979_2021-04-19
G32	4	4	0	100.0	0.0	SouthAfrica	2020-12-09 ; 2021-01-07	Mozambique/INS-K007985/2021_EPI_ISL_887452_2021-01-07, Mozambique/INS-K007988/2021_EPI_ISL_887453_2021-01-07, Mozambique/INS-K007984/2021_EPI_ISL_887451_2021-01-07, Mozambique/INS-K007986/2021_EPI_ISL_887433_2021-01-07
G33	4	4	4	100.0	100.0	Zimbabwe	2020-12-17 ; 2021-02-28	Mozambique/MZ-L-IBV-97025278/2021_EPI_ISL_2442085_2021-03-03, Mozambique/MZ-L-IBV-97025465/2021_EPI_ISL_3246529_2021-02-28, Mozambique/MZ-L-IBV-97025464/2021_EPI_ISL_2442201_2021-02-28, Mozambique/MZ-L-IBV-97025463/2021_EPI_ISL_2442200_2021-02-28
G34	5	5	4	100.0	80.0	SouthAfrica	2020-12-09 ; 2021-01-07	Mozambique/INS-K007999/2021_EPI_ISL_887454_2021-01-07, Mozambique/MZ-L-IBV-97025352/2021_EPI_ISL_2442115_2021-01-12, Mozambique/MZ-L-IBV-97025375/2021_EPI_ISL_3246526_2021-01-23, Mozambique/MZ-L-IBV-97025398/2021_EPI_ISL_3246531_2021-01-25, Mozambique/MZ-L-IBV-97025416/2021_EPI_ISL_2442162_2021-01-15
G35	5	5	0	100.0	0.0	SouthAfrica	2020-12-21 ; 2021-03-12	Mozambique/CERI-KRISP-K015048/2021_EPI_ISL_2617126_2021-03-17, Mozambique/CERI-KRISP-K015029/2021_EPI_ISL_2617094_2021-03-12, Mozambique/CERI-KRISP-K015072/2021_EPI_ISL_2617119_2021-03-18, Mozambique/CERI-KRISP-K016085/2021_EPI_ISL_3447991_2021-04-20, Mozambique/CERI-KRISP-K015034/2021_EPI_ISL_2617128_2021-03-12
G36	6	6	6	100.0	100.0	Malawi	2021-01-12 ; 2021-01-19	Mozambique/MZ-L-IBV-97025403/2021_EPI_ISL_2442150_2021-01-21, Mozambique/MZ-L-IBV-97025359/2021_EPI_ISL_2442119_2021-02-09, Mozambique/MZ-L-IBV-97025342/2021_EPI_ISL_2442107_2021-02-09, Mozambique/MZ-L-IBV-97025347/2021_EPI_ISL_2442111_2021-02-25, Mozambique/MZ-L-IBV-97025425/2021_EPI_ISL_2442171_2021-01-19, Mozambique/MZ-L-IBV-97025310/2021_EPI_ISL_2442098_2021-02-17
G37	6	6	0	100.0	0.0	Undetermined	Before 2020-08-26	Mozambique/INS-K011854/2021_EPI_ISL_2396959_2021-02-19, Mozambique/INS-K011840/2020_EPI_ISL_2396945_2020-11-04, Mozambique/INS-K011842/2020_EPI_ISL_2396947_2020-11-04, Mozambique/INS-K011777/2020_EPI_ISL_2396891_2020-08-26, Mozambique/INS-K011855/2021_EPI_ISL_2396960_2021-02-08, Mozambique/INS-K011871/2021_EPI_ISL_2396976_2021-02-23
G38	7	7	6	100.0	85.7	SouthAfrica	2020-12-28 ; 2021-01-24	Mozambique/MZ-L-IBV-97025394/2021_EPI_ISL_2442144_2021-01-24, Mozambique/MZ-L-IBV-97025391/2021_EPI_ISL_2442142_2021-01-24, Mozambique/CERI-KRISP-K016070/2021_EPI_ISL_3447978_2021-04-20, Mozambique/MZ-L-IBV-97025370/2021_EPI_ISL_2442126_2021-01-24, Mozambique/MZ-L-IBV-97025418/2021_EPI_ISL_2442164_2021-01-24, Mozambique/MZ-L-IBV-97025380/2021_EPI_ISL_2442135_2021-01-24, Mozambique/MZ-L-IBV-97025387/2021_EPI_ISL_2442139_2021-01-24
G39	8	10	0	80.0	0.0	Undetermined	Before 2020-08-25	SouthAfrica/KRISP-K011947/2021_EPI_ISL_2397052_2021-03-03, Mozambique/INS-K011877/2021_EPI_ISL_2396982_2021-02-05, Mozambique/INS-K011805/2020_EPI_ISL_2396910_2020-10-10, Mozambique/INS-K011847/2021_EPI_ISL_2396952_2021-02-05, Mozambique/INS-K011778/2020_EPI_ISL_2396892_2020-08-25, Mozambique/INS-K011883/2021_EPI_ISL_2396988_2021-02-08, Mozambique/INS-K011803/2020_EPI_ISL_2396908_2020-11-09, Mozambique/INS-K011873/2021_EPI_ISL_2396978_2021-02-05, SouthAfrica/KRISP-K011950/2021_EPI_ISL_2397055_2021-03-08, Mozambique/INS-K011879/2021_EPI_ISL_2396984_2021-02-05
G40	9	11	0	81.8	0.0	SouthAfrica	2020-12-21 ; 2021-01-22	Mozambique/INS-K008797/2021_EPI_ISL_1132836_2021-01-22, Mozambique/CERI-KRISP-K015061/2021_EPI_ISL_2617117_2021-03-17, Mozambique/CERI-KRISP-K015011/2021_EPI_ISL_2617141_2021-03-10, Mozambique/CERI-KRISP-K015038/2021_EPI_ISL_2617124_2021-03-12, Mozambique/CERI-KRISP-K015062/2021_EPI_ISL_2617098_2021-03-17, Mozambique/CERI-KRISP-K016047/2021_EPI_ISL_3447986_2021-04-16, Mozambique/CERI-KRISP-K015999/2021_EPI_ISL_3447975_2021-03-31, Mozambique/CERI-KRISP-K015014/2021_EPI_ISL_2617172_2021-03-10, Mozambique/CERI-KRISP-K015012/2021_EPI_ISL_2617110_2021-03-10, MWI/MLW00246/2021_OL779176.1_2021-03-26, Malawi/MLW-00095/2021_EPI_ISL_6963027_2021-03-26
G41	10	15	0	66.7	0.0	Undetermined	Before 2020-10-10	Mozambique/INS-K011866/2021_EPI_ISL_2396971_2021-02-08, Mozambique/INS-K011793/2020_EPI_ISL_2396898_2020-10-10, Mozambique/INS-K011818/2020_EPI_ISL_2396923_2020-10-21, SouthAfrica/KRISP-K012085/2021_EPI_ISL_2397094_2021-03-19, Mozambique/INS-K011810/2020_EPI_ISL_2396915_2020-10-10, SouthAfrica/KRISP-K011939/2021_EPI_ISL_2397044_2021-03-09, Mozambique/INS-K011845/2021_EPI_ISL_2396950_2021-02-07, SouthAfrica/KRISP-K014586/2021_EPI_ISL_2494531_2021-04-27, SouthAfrica/KRISP-K014593/2021_EPI_ISL_2494613_2021-04-20, Mozambique/INS-K011844/2020_EPI_ISL_2396949_2020-11-03, SouthAfrica/KRISP-K011932/2021_EPI_ISL_2397037_2021-03-08, Mozambique/INS-K011846/2021_EPI_ISL_2396951_2021-02-06, Mozambique/INS-K011841/2020_EPI_ISL_2396946_2020-11-05, Mozambique/INS-K011837/2020_EPI_ISL_2396942_2020-11-04, Mozambique/INS-K011839/2020_EPI_ISL_2396944_2020-11-04
G42	15	17	10	88.2	66.7	SouthAfrica	2020-11-17 ; 2020-12-30	Mozambique/INS-K008160/2021_EPI_ISL_964926_2021-01-15, Mozambique/INS-K008785/2021_EPI_ISL_1132831_2021-01-18, Mozambique/MZ-L-IBV-97025303/2021_EPI_ISL_2442093_2021-02-17, Mozambique/MZ-L-IBV-97025285/2021_EPI_ISL_2442088_2021-02-01, Mozambique/MZ-L-IBV-97025395/2021_EPI_ISL_2442145_2021-01-24, Mozambique/MZ-L-IBV-97025390/2021_EPI_ISL_2442141_2021-01-24, Mozambique/MZ-L-IBV-97025389/2021_EPI_ISL_3246528_2021-01-24, Mozambique/MZ-L-IBV-97025396/2021_EPI_ISL_2442146_2021-01-24, Mozambique/MZ-L-IBV-97025381/2021_EPI_ISL_2442136_2021-01-24, Mozambique/MZ-L-IBV-97025388/2021_EPI_ISL_2442140_2021-01-24, Mozambique/CERI-KRISP-K015041/2021_EPI_ISL_2617108_2021-03-16, Mozambique/MZ-L-IBV-97025455/2021_EPI_ISL_2442196_2021-02-02, SouthAfrica/VIDA-KRISP-K008050/2020_EPI_ISL_940873_2020-12-30, Mozambique/INS-K008149/2021_EPI_ISL_964921_2021-01-14, Mozambique/INS-K008161/2021_EPI_ISL_964938_2021-01-22, Mozambique/MZ-L-IBV-97025341/2021_EPI_ISL_3246522_2021-02-09, Senegal/2027667/2021_EPI_ISL_8527645_2021-01-09
**total_samples_group**	**150**		**49**					

Beta unique sequences			
**Strain**	**Likely_geographical_origin**	**Likely_introduction_period**	**new_seq**
COV035979	undetermined	before 2021-05-05	**yes**
Mozambique/CERI-KRISP-K014987/2021_2021-03-01	SouthAfrica	2021-02-16 ; 2021-03-01	no
Mozambique/CERI-KRISP-K014990/2021_EPI_ISL_2492957_2021-03-05	Eswatini/SouthAfrica	2021-02-16 ; 2021-03-05	no
Mozambique/CERI-KRISP-K014995/2021_EPI_ISL_2492976_2021-03-05	SouthAfrica	2021-01-04 ; 2021-03-05	no
Mozambique/CERI-KRISP-K014997/2021_EPI_ISL_2492968_2021-03-05	SouthAfrica	2021-01-06 ; 2021-03-05	no
Mozambique/CERI-KRISP-K014998/2021_EPI_ISL_2492997_2021-03-05	SouthAfrica	2021-02-04 ; 2021-03-05	no
Mozambique/CERI-KRISP-K015003/2021_EPI_ISL_2617135_2021-03-08	Kenya	2021-03-03 ; 2021-03-08	no
Mozambique/CERI-KRISP-K015004/2021_EPI_ISL_2617118_2021-03-09	SouthAfrica	2021-01-11 ; 2021-03-09	no
Mozambique/CERI-KRISP-K015007/2021_EPI_ISL_2617168_2021-03-09	undetermined	before 2021-03-09	no
Mozambique/CERI-KRISP-K015008/2021_EPI_ISL_2617095_2021-03-09	SouthAfrica	2021-01-09 ; 2021-03-09	no
Mozambique/CERI-KRISP-K015010/2021_EPI_ISL_2617125_2021-03-09	SouthAfrica/Ghana	2021-01-15 ; 2021-03-09	no
Mozambique/CERI-KRISP-K015017/2021_EPI_ISL_2617162_2021-03-11	SouthAfrica	2021-01-12 ; 2021-03-11	no
Mozambique/CERI-KRISP-K015018/2021_EPI_ISL_2617159_2021-03-11	undetermined	before 2021-03-11	no
Mozambique/CERI-KRISP-K015020/2021_EPI_ISL_2617109_2021-03-11	SouthAfrica	2021-01-12 ; 2021-03-11	no
Mozambique/CERI-KRISP-K015021/2021_EPI_ISL_2617185_2021-03-11	undetermined	before 2021-03-11	no
Mozambique/CERI-KRISP-K015026/2021_EPI_ISL_2617115_2021-03-12	undetermined	before 2021-03-12	no
Mozambique/CERI-KRISP-K015035/2021_EPI_ISL_2617120_2021-03-12	Malawi	2021-03-05 ; 2021-03-12	no
Mozambique/CERI-KRISP-K015036/2021_EPI_ISL_2617130_2021-03-12	Angola	2021-01-28 ; 2021-03-12	no
Mozambique/CERI-KRISP-K015042/2021_EPI_ISL_2617131_2021-03-16	SouthAfrica	2021-01-12 ; 2021-03-16	no
Mozambique/CERI-KRISP-K015046/2021_EPI_ISL_2617154_2021-03-17	SouthAfrica/Angola	2021-02-04 ; 2021-03-17	no
Mozambique/CERI-KRISP-K015059/2021_EPI_ISL_2617132_2021-03-18	SouthAfrica	2021-03-14 ; 2021-03-18	no
Mozambique/CERI-KRISP-K015065/2021_EPI_ISL_2617122_2021-03-18	undetermined	before 2021-03-18	no
Mozambique/CERI-KRISP-K015068/2021_EPI_ISL_2617113_2021-03-18	SouthAfrica	2021-03-14 ; 2021-03-18	no
Mozambique/CERI-KRISP-K015069/2021_EPI_ISL_2617096_2021-03-18	Malawi	2021-03-05 ; 2021-03-18	no
Mozambique/CERI-KRISP-K015075/2021_EPI_ISL_2617099_2021-03-18	SouthAfrica	2021-02-16 ; 2021-03-18	no
Mozambique/CERI-KRISP-K015076/2021_EPI_ISL_2617144_2021-03-18	SouthAfrica/Angola	2021-02-04 ; 2021-03-18	no
Mozambique/CERI-KRISP-K015079/2021_EPI_ISL_2617136_2021-03-18	undetermined	before 2021-03-18	no

Mozambique/CERI-KRISP-K015081/2021_EPI_ISL_2617106_2021-03-18	SouthAfrica/Angola	2021-02-04 ; 2021-03-18	no
Mozambique/CERI-KRISP-K015086/2021_EPI_ISL_2617127_2021-03-21	Gabon	2021-02-09 ; 2021-03-21	no
Mozambique/CERI-KRISP-K015087/2021_EPI_ISL_2617123_2021-03-21	Qatar	2021-02-20 ; 2021-03-21	no
Mozambique/CERI-KRISP-K015088/2021_EPI_ISL_2617129_2021-03-21	France	2021-01-20 ; 2021-03-21	no
Mozambique/CERI-KRISP-K016000/2021_EPI_ISL_3447988_2021-04-02	undetermined	before 2021-04-02	no
Mozambique/CERI-KRISP-K016001/2021_EPI_ISL_3447993_2021-03-30	SouthAfrica	2021-02-16 ; 2021-03-30	no
Mozambique/CERI-KRISP-K016003/2021_EPI_ISL_3447989_2021-03-30	undetermined	before 2021-03-30	no
Mozambique/CERI-KRISP-K016004/2021_EPI_ISL_3447992_2021-03-25	undetermined	before 2021-03-25	no
Mozambique/CERI-KRISP-K016005/2021_EPI_ISL_3447994_2021-03-25	SouthAfrica	2021-02-16 ; 2021-03-25	no
Mozambique/CERI-KRISP-K016006/2021_EPI_ISL_3447995_2021-03-31	undetermined	before 2021-03-31	no
Mozambique/CERI-KRISP-K016015/2021_EPI_ISL_3447980_2021-04-01	undetermined	before 2021-04-01	no
Mozambique/CERI-KRISP-K016042/2021_EPI_ISL_3447990_2021-04-13	undetermined	before 2021-04-13	no
Mozambique/CERI-KRISP-K016046/2021_EPI_ISL_3447981_2021-04-14	undetermined	before 2021-04-14	no
Mozambique/CERI-KRISP-K016053/2021_EPI_ISL_3447982_2021-04-16	SouthAfrica	2021-02-17 ; 2021-04-16	no
Mozambique/CERI-KRISP-K016054/2021_2021-04-18	undetermined	before 2021-04-18	no
Mozambique/CERI-KRISP-K016056/2021_EPI_ISL_3447984_2021-04-13	undetermined	before 2021-04-13	no
Mozambique/CERI-KRISP-K016058/2021_EPI_ISL_3447997_2021-04-16	undetermined	before 2021-04-16	no
Mozambique/CERI-KRISP-K016071/2021_EPI_ISL_3447987_2021-04-20	SouthAfrica	2021-03-14 ; 2021-04-20	no
Mozambique/CERI-KRISP-K016074/2021_EPI_ISL_3447974_2021-04-20	SouthAfrica/Portugal	2021-03-04 ; 2021-04-20	no
Mozambique/CERI-KRISP-K016077/2021_EPI_ISL_3447983_2021-04-20	SouthAfrica	2021-03-14 ; 2021-04-20	no
Mozambique/CERI-KRISP-K018287/2021_EPI_ISL_2688576_2021-04-20	Portugal	2021-02-26 ; 2021-04-20	no
Mozambique/CERI-KRISP-K018292/2021_EPI_ISL_2688578_2021-04-22	undetermined	before 2021-04-22	no
Mozambique/CERI-KRISP-K027176/2021_EPI_ISL_8799665_2021-07-15	undetermined	before 2021-07-15	no
Mozambique/COV-2140-A1787/2021_EPI_ISL_5822550_2021-06-18	Botswana/SouthAfrica	2021-05-18 ; 2021-06-18	no
Mozambique/COV-2185-A1832/2021_EPI_ISL_5822551_2021-06-21	undetermined	before 2021-06-21	no
Mozambique/COV-2297-A1945/2021_EPI_ISL_5822661_2021-05-31	SouthAfrica	2021-05-05 ; 2021-05-31	no
Mozambique/COV-2306-A1954/2021_EPI_ISL_5822697_2021-05-18	undetermined	before 2021-05-18	no
Mozambique/COV-2321-A1969/2021_EPI_ISL_5822568_2021-04-30	SouthAfrica	2021-04-12 ; 2021-04-30	no
Mozambique/COV-2342-A1991/2021_EPI_ISL_5822558_2021-04-26	Bangladesh	2021-03-14 ; 2021-04-26	no
Mozambique/COV-2344-A1993/2021_2021-04-30_2021-11-01	SouthAfrica	2021-03-14 ; 2021-04-30	no

Mozambique/COV-2360-A2009/2021_EPI_ISL_5822685_2021-05-27	undetermined	before 2021-05-27	no
Mozambique/COV-2373-A2022/2021_EPI_ISL_5822563_2021-04-29	SouthAfrica	2021-04-12 ; 2021-04-29	no
Mozambique/COV-2374-A2023/2021_EPI_ISL_5822564_2021-04-01	undetermined	before 2021-04-01	no
Mozambique/INS-K007976/2021_EPI_ISL_887448_2021-01-07	SouthAfrica	2020-12-28 ; 2021-01-07	no
Mozambique/INS-K007977/2021_EPI_ISL_887449_2021-01-07	SouthAfrica	2020-11-24 ; 2021-01-07	no
Mozambique/INS-K007980/2021_EPI_ISL_887419_2021-01-06	SouthAfrica	2020-12-18 ; 2021-01-06	no
Mozambique/INS-K007981/2021_EPI_ISL_887450_2021-01-06	undetermined	before 2021-01-06	no
Mozambique/INS-K007989/2021_EPI_ISL_887427_2021-01-07	SouthAfrica	2020-12-15 ; 2021-01-07	no
Mozambique/INS-K008128/2021_EPI_ISL_964934_2021-01-16	Zimbabwe	2020-12-29 ; 2021-01-16	no
Mozambique/INS-K008131/2021_EPI_ISL_964922_2021-01-16	SouthAfrica	2020-12-09 ; 2021-01-16	no
Mozambique/INS-K008132/2021_EPI_ISL_964936_2021-01-16	UK	2021-01-09 ; 2021-01-16	no
Mozambique/INS-K008143/2021_EPI_ISL_964929_2021-01-15	SouthAfrica/UK	2020-12-21 ; 2021-01-15	no
Mozambique/INS-K008146/2021_EPI_ISL_964931_2021-01-15	SouthAfrica	2020-11-20 ; 2021-01-15	no
Mozambique/INS-K008147/2021_EPI_ISL_964925_2021-01-16	undetermined	before 2021-01-16	no
Mozambique/INS-K008154/2021_EPI_ISL_964932_2021-01-14	SouthAfrica/Ireland	2020-11-20 ; 2021-01-14	no
Mozambique/INS-K008162/2021_EPI_ISL_964939_2021-01-15	SouthAfrica	2021-01-02 ; 2021-01-15	no
Mozambique/INS-K008166/2021_EPI_ISL_964927_2021-01-17	SouthAfrica	2020-12-15 ; 2021-01-17	no
Mozambique/INS-K008170/2021_EPI_ISL_964941_2021-01-14	SouthAfrica	2020-12-26 ; 2021-01-14	no
Mozambique/INS-K008173/2021_EPI_ISL_964942_2021-01-16	SouthAfrica	2020-12-30 ; 2021-01-16	no
Mozambique/INS-K008771/2021_EPI_ISL_1132818_2021-01-14	Eswatini/SouthAfrica	2020-11-23 ; 2021-01-14	no
Mozambique/INS-K008772/2021_EPI_ISL_1132819_2021-01-28	Malawi	2021-01-12 ; 2021-01-28	no
Mozambique/INS-K008774/2021_EPI_ISL_1132821_2021-01-10	undetermined	before 2021-01-10	no
Mozambique/INS-K008777/2021_EPI_ISL_1132824_2021-01-30	SouthAfrica	2020-12-21 ; 2021-01-30	no
Mozambique/INS-K008778/2021_EPI_ISL_1132825_2021-01-30	SouthAfrica	2020-12-12 ; 2021-01-30	no
Mozambique/INS-K008781/2021_EPI_ISL_1132828_2021-01-30	undetermined	before 2021-01-30	no
Mozambique/INS-K008783/2021_EPI_ISL_1132829_2021-01-13	SouthAfrica	2020-12-27 ; 2021-01-13	no
Mozambique/INS-K008791/2021_EPI_ISL_1132834_2021-01-10	SouthAfrica	2020-12-21 ; 2021-01-10	no
Mozambique/INS-K008809/2021_EPI_ISL_1132841_2021-01-16	SouthAfrica	2020-12-21 ; 2021-01-16	no
Mozambique/INS-K011781/2020_EPI_ISL_2396895_2020-08-22	undetermined	before 2020-08-22	no
Mozambique/INS-K011807/2020_EPI_ISL_2396912_2020-11-10	undetermined	before 2020-11-10	no

Mozambique/INS-K011809/2020_EPI_ISL_2396914_2020-10-10	undetermined	before 2020-10-10	no
Mozambique/INS-K011819/2020_EPI_ISL_2396924_2020-10-30	undetermined	before 2020-10-30	no
Mozambique/INS-K011822/2020_EPI_ISL_2396927_2020-10-31	undetermined	before 2020-10-31	no
Mozambique/INS-K011831/2020_EPI_ISL_2396936_2020-11-03	undetermined	before 2020-11-03	no
Mozambique/INS-K011834/2020_EPI_ISL_2396939_2020-11-05	undetermined	before 2020-11-05	no
Mozambique/INS-K011848/2021_EPI_ISL_2396953_2021-02-06	SouthAfrica	2020-12-21 ; 2021-02-06	no
Mozambique/INS-K011849/2021_EPI_ISL_2396954_2021-02-19	SouthAfrica/Slovenia	2020-12-24 ; 2021-02-19	no
Mozambique/INS-K011856/2021_EPI_ISL_2396961_2021-02-06	SouthAfrica	2021-01-01 ; 2021-02-06	no
Mozambique/INS-K011857/2021_EPI_ISL_2396962_2021-02-08	SouthAfrica	2020-12-21 ; 2021-02-08	no
Mozambique/INS-K011859/2021_2021-02-08	Zimbabwe/SouthAfrica	2020-12-17 ; 2021-02-08	no
Mozambique/INS-K011868/2021_EPI_ISL_2396973_2021-02-22	SouthAfrica	2021-01-04 ; 2021-02-22	no
Mozambique/INS-K011872/2021_EPI_ISL_2396977_2021-02-08	undetermined	before 2021-02-08	no
Mozambique/INS-K011881/2021_EPI_ISL_2396986_2021-02-19	SouthAfrica/UK	2021-01-11 ; 2021-02-19	no
Mozambique/INS-K011884/2021_EPI_ISL_2396989_2021-02-08	SouthAfrica	2020-12-21 ; 2021-02-08	no
Mozambique/INS-K011888/2021_2021-02-09	undetermined	before 2021-02-09	no
Mozambique/INS-K011889/2021_2021-02-08	undetermined	before 2021-02-08	no
Mozambique/INS-K011891/2021_EPI_ISL_2396996_2021-02-02	SouthAfrica	2021-01-13 ; 2021-02-02	no
Mozambique/INS-K011893/2021_2021-02-06	undetermined	before 2021-02-06	no
Mozambique/INS-K011894/2021_EPI_ISL_2396999_2021-02-08	India	2021-02-02 ; 2021-02-08	no
Mozambique/MZ-L-IBV-97025276/2021_EPI_ISL_2442084_2021-02-01	SouthAfrica	2020-12-24 ; 2021-02-01	**yes**
Mozambique/MZ-L-IBV-97025290/2021_EPI_ISL_2442089_2021-02-01	SouthAfrica	2020-12-24 ; 2021-02-01	**yes**
Mozambique/MZ-L-IBV-97025296/2021_EPI_ISL_2442090_2021-02-20	SouthAfrica/Gabon	2020-12-28 ; 2021-02-20	**yes**
Mozambique/MZ-L-IBV-97025304/2021_EPI_ISL_2442094_2021-02-19	Angola	2021-01-28 ; 2021-02-19	**yes**
Mozambique/MZ-L-IBV-97025309/2021_EPI_ISL_2442097_2021-02-17	SouthAfrica	2021-01-03 ; 2021-02-17	**yes**
Mozambique/MZ-L-IBV-97025311/2021_EPI_ISL_2442099_2021-02-12	Zimbabwe/SouthAfrica	2020-12-14 ; 2021-02-12	**yes**
Mozambique/MZ-L-IBV-97025315/2021_EPI_ISL_2442100_2021-02-12	SouthAfrica	2021-01-11 ; 2021-02-12	**yes**
Mozambique/MZ-L-IBV-97025321/2021_2021-02-12	undetermined	before 2021-02-12	**yes**
Mozambique/MZ-L-IBV-97025326/2021_2021-02-18	undetermined	before 2021-02-18	**yes**
Mozambique/MZ-L-IBV-97025343/2021_EPI_ISL_2442108_2021-02-02	SouthAfrica	2021-01-05 ; 2021-02-02	**yes**
Mozambique/MZ-L-IBV-97025346/2021_EPI_ISL_2442110_2021-02-01	SouthAfrica	2020-12-21 ; 2021-02-01	**yes**

Mozambique/MZ-L-IBV-97025351/2021_EPI_ISL_2442114_2021-01-12	SouthAfrica	2020-12-07 ; 2021-01-12	**yes**
Mozambique/MZ-L-IBV-97025354/2021_EPI_ISL_2442117_2021-01-14	SouthAfrica	2020-12-27 ; 2021-01-14	**yes**
Mozambique/MZ-L-IBV-97025356/2021_EPI_ISL_3246523_2021-01-12	SouthAfrica	2020-12-30 ; 2021-01-12	**yes**
Mozambique/MZ-L-IBV-97025357/2021_EPI_ISL_2442118_2021-01-08	SouthAfrica	2020-12-30 ; 2021-01-08	**yes**
Mozambique/MZ-L-IBV-97025358/2021_EPI_ISL_3246524_2021-02-09	SouthAfrica	2021-01-06 ; 2021-02-09	**yes**
Mozambique/MZ-L-IBV-97025364/2021_EPI_ISL_2442122_2021-01-23	SouthAfrica	2020-12-21 ; 2021-01-23	**yes**
Mozambique/MZ-L-IBV-97025365/2021_EPI_ISL_2442123_2021-01-23	SouthAfrica	2020-12-24 ; 2021-01-23	**yes**
Mozambique/MZ-L-IBV-97025374/2021_EPI_ISL_2442130_2021-01-23	undetermined	before 2021-01-23	**yes**
Mozambique/MZ-L-IBV-97025393/2021_2021-01-25	undetermined	before 2021-01-25	**yes**
Mozambique/MZ-L-IBV-97025399/2021_EPI_ISL_2442147_2021-01-22	SouthAfrica	2020-12-15 ; 2021-01-22	**yes**
Mozambique/MZ-L-IBV-97025402/2021_EPI_ISL_2442149_2021-01-19	undetermined	before 2021-01-19	**yes**
Mozambique/MZ-L-IBV-97025409/2021_EPI_ISL_2442155_2021-01-13	SouthAfrica	2021-01-03 ; 2021-01-13	**yes**
Mozambique/MZ-L-IBV-97025410/2021_EPI_ISL_2442156_2021-01-13	SouthAfrica	2020-12-21 ; 2021-01-13	**yes**
Mozambique/MZ-L-IBV-97025412/2021_EPI_ISL_2442158_2021-01-13	SouthAfrica	2020-12-18 ; 2021-01-13	**yes**
Mozambique/MZ-L-IBV-97025414/2021_2021-01-13	SouthAfrica	2020-12-15 ; 2021-01-13	**yes**
Mozambique/MZ-L-IBV-97025415/2021_EPI_ISL_2442161_2021-01-13	SouthAfrica	2020-12-07 ; 2021-01-13	**yes**
Mozambique/MZ-L-IBV-97025419/2021_EPI_ISL_2442165_2021-01-18	undetermined	before 2021-01-18	**yes**
Mozambique/MZ-L-IBV-97025422/2021_EPI_ISL_2442168_2021-01-19	SouthAfrica/UK	2020-12-15 ; 2021-01-19	**yes**
Mozambique/MZ-L-IBV-97025424/2021_EPI_ISL_2442170_2021-01-19	SouthAfrica	2020-11-20 ; 2021-01-19	**yes**
Mozambique/MZ-L-IBV-97025427/2020_EPI_ISL_2442173_2020-12-30	SouthAfrica	2020-12-28 ; 2020-12-30	**yes**
Mozambique/MZ-L-IBV-97025428/2021_EPI_ISL_2442174_2021-01-14	SouthAfrica	2020-12-30 ; 2021-01-14	**yes**
Mozambique/MZ-L-IBV-97025431/2021_EPI_ISL_2442177_2021-01-14	SouthAfrica	2020-12-01 ; 2021-01-14	**yes**
Mozambique/MZ-L-IBV-97025440/2021_EPI_ISL_2442185_2021-01-08	SouthAfrica	2021-01-04 ; 2021-01-08	**yes**
Mozambique/MZ-L-IBV-97025454/2021_EPI_ISL_2442195_2021-02-01	SouthAfrica	2020-12-24 ; 2021-02-01	**yes**
Mozambique/MZ-L-IBV-97025458/2021_EPI_ISL_2442197_2021-02-02	SouthAfrica	2020-12-11 ; 2021-02-02	**yes**
Mozambique/MZ-L-IBV-97025459/2021_EPI_ISL_2442198_2021-02-01	SouthAfrica	2020-12-15 ; 2021-02-01	**yes**
Mozambique/MZ-L-IBV-97025461/2021_EPI_ISL_2442199_2021-02-02	SouthAfrica	2020-12-21 ; 2021-02-02	**yes**
Mozambique/MZ-L-IBV-97025466/2021_EPI_ISL_2442202_2021-02-26	SouthAfrica	2020-12-27 ; 2021-02-26	**yes**
**total_Mozambican_seqs_uniques**	**145**		
**new_seqs_uniques**	**40**		

Beta unclassified sequences	
**Sample_name**	**new_seq**
Mozambique/INS-K007927/2020_EPI_ISL_887443_2020-12-26	no
Mozambique/INS-K011804/2020_EPI_ISL_2396909_2020-11-09	no
Mozambique/INS-K011833/2020_EPI_ISL_2396938_2020-11-04	no
Mozambique/INS-K011824/2020_EPI_ISL_2396929_2020-10-31	no
Mozambique/INS-K011817/2020_EPI_ISL_2396922_2020-10-16	no
Mozambique/INS-K011821/2020_EPI_ISL_2396926_2020-10-27	no
Mozambique/INS-K011826/2020_EPI_ISL_2396931_2020-11-02	no
Mozambique/INS-K011830/2020_EPI_ISL_2396935_2020-11-04	no
Mozambique/INS-K011829/2020_EPI_ISL_2396934_2020-11-03	no
Mozambique/INS-K011828/2020_EPI_ISL_2396933_2020-11-03	no
Mozambique/INS-K011820/2020_EPI_ISL_2396925_2020-10-27	no
Mozambique/INS-K011876/2021_EPI_ISL_2396981_2021-02-06	no
Mozambique/INS-K011869/2021_EPI_ISL_2396974_2021-02-05	no
Mozambique/INS-K011776/2020_EPI_ISL_2396890_2020-08-25	no
Mozambique/INS-K011865/2021_EPI_ISL_2396970_2021-02-05	no
Mozambique/INS-K011861/2021_EPI_ISL_2396966_2021-02-08	no
Mozambique/INS-K011851/2021_EPI_ISL_2396956_2021-02-08	no
hCoV-19/Mozambique/INS-K011850/2021_2021-02-08_2021-06-03	no
hCoV-19/Mozambique/INS-K011870/2021_2021-02-05_2021-06-03	no
Mozambique/INS-K011864/2021_EPI_ISL_2396969_2021-02-18	no
hCoV-19/Mozambique/INS-K011852/2021_2021-02-04_2021-06-03	no
Mozambique/INS-K011863/2021_EPI_ISL_2396968_2021-02-19	no
Mozambique/INS-K011835/2020_EPI_ISL_2396940_2020-11-04	no
Mozambique/INS-K011836/2020_EPI_ISL_2396941_2020-11-04	no
Mozambique/MZ-L-IBV-97025417/2021_EPI_ISL_2442163_2021-01-14	**yes**
Mozambique/INS-K008790/2021_EPI_ISL_1132833_2021-01-18	no
Mozambique/INS-K007925/2020_EPI_ISL_887442_2020-12-26	no

Mozambique/MZ-L-IBV-97025452/2021_EPI_ISL_2442193_2021-01-18	**yes**
Mozambique/MZ-L-IBV-97025353/2021_EPI_ISL_2442116_2021-01-14	**yes**
Mozambique/MZ-L-IBV-97025349/2021_EPI_ISL_2442112_2021-02-01	**yes**
Mozambique/MZ-L-IBV-97025361/2021_EPI_ISL_2442120_2021-01-12	**yes**
Mozambique/INS-K007928/2020_EPI_ISL_887444_2020-12-16	no
Mozambique/MZ-L-IBV-97025368/2021_EPI_ISL_2442127_2021-01-23	**yes**
Mozambique/MZ-L-IBV-97025376/2021_EPI_ISL_2442131_2021-01-23	**yes**
Mozambique/MZ-L-IBV-97025371/2021_EPI_ISL_2442128_2021-01-23	**yes**
hCoV-19/Mozambique/MZ-L-IBV-97025386/2021_2021-01-24_2021-06-07	**yes**
Mozambique/INS-K007922/2020_EPI_ISL_887441_2020-12-26	no
hCoV-19/Mozambique/MZ-L-IBV-97025366/2021_2021-01-20_2021-06-07	**yes**
hCoV-19/Mozambique/INS-K011885/2021_2021-02-08_2021-06-03	no
Mozambique/INS-K011867/2021_EPI_ISL_2396972_2021-02-08	no
Mozambique/INS-K008133/2021_EPI_ISL_964937_2021-01-16	no
Mozambique/INS-K011890/2021_EPI_ISL_2396995_2021-02-08	no
Mozambique/INS-K011827/2020_EPI_ISL_2396932_2020-11-15	no
Mozambique/INS-K011875/2021_EPI_ISL_2396980_2021-02-05	no
Mozambique/INS-K011860/2021_EPI_ISL_2396965_2021-02-08	no
Mozambique/INS-K011878/2021_EPI_ISL_2396983_2021-02-09	no
hCoV-19/Mozambique/INS-K011882/2021_2021-02-08_2021-06-03	no
Mozambique/INS-K011838/2020_EPI_ISL_2396943_2020-11-04	no
Mozambique/MZ-L-IBV-97025405/2021_EPI_ISL_2442152_2021-01-05	**yes**
Mozambique/MZ-L-IBV-97025350/2021_EPI_ISL_2442113_2021-01-14	**yes**
Mozambique/MZ-L-IBV-97025420/2021_EPI_ISL_2442166_2021-01-19	**yes**
Mozambique/INS-K007930/2020_EPI_ISL_887445_2020-12-14	no
Mozambique/MZ-L-IBV-97025373/2021_EPI_ISL_2442129_2021-01-23	**yes**
hCoV-19/Mozambique/MZ-L-IBV-97025383/2021_2021-01-24_2021-08-05	**yes**
Mozambique/MZ-L-IBV-97025411/2021_EPI_ISL_2442157_2021-01-13	**yes**
hCoV-19/Mozambique/MZ-L-IBV-97025362/2021_2021-01-06_2021-08-05	**yes**
Mozambique/INS-K011874/2021_EPI_ISL_2396979_2021-02-08	no

COV035981	**yes**
Mozambique/CERI-KRISP-K014985/2021_EPI_ISL_2492927_2021-01-01	no
Mozambique/INS-K007939/2020_EPI_ISL_887447_2020-12-26	no
Mozambique/INS-K008150/2021_EPI_ISL_964923_2021-01-15	no
Mozambique/INS-K008171/2021_EPI_ISL_964928_2021-01-16	no
Mozambique/INS-K008779/2021_EPI_ISL_1132826_2021-01-30	no
Mozambique/INS-K008780/2021_EPI_ISL_1132827_2021-01-30	no
Mozambique/INS-K008805/2021_EPI_ISL_1132840_2021-01-16	no
Mozambique/INS-K008810/2021_EPI_ISL_1132842_2021-01-16	no
Mozambique/INS-K011780/2020_EPI_ISL_2396894_2020-08-25	no
Mozambique/INS-K011801/2020_EPI_ISL_2396906_2020-10-09	no
Mozambique/INS-K011815/2020_EPI_ISL_2396920_2020-10-31	no
Mozambique/INS-K011816/2020_EPI_ISL_2396921_2020-10-30	no
Mozambique/INS-K011832/2020_EPI_ISL_2396937_2020-11-03	no
Mozambique/INS-K011887/2021_2021-02-08	no
Mozambique/INS-K011892/2021_EPI_ISL_2396997_2021-02-05	no
Mozambique/MZ-L-IBV-97025305/2021_2021-02-17	**yes**
Mozambique/MZ-L-IBV-97025340/2021_EPI_ISL_2442106_2021-02-01	**yes**
Mozambique/MZ-L-IBV-97025385/2021_EPI_ISL_2442137_2021-01-24	**yes**
Mozambique/MZ-L-IBV-97025429/2021_EPI_ISL_2442175_2021-01-14	**yes**
Mozambique/MZ-L-IBV-97025434/2021_EPI_ISL_2442179_2021-01-15	**yes**
**total_Mozambican_seqs**	**78**
**new_seqs**	**23**

Delta transmission groups										
**Group**	**samples_Mz**	**cluster_size**	**samples_NewIBV**	**%Mz**	**%Mz_New_IBV**	**Likely_geographical_origin**	**Likely_introduction_period**	**lineage**	**Samples_included**	**Comments**

G1	60	97	11	61.86	18.33	UK	2021-04-19 ; 2021-05-27	AY.6	Canada/QC-L00364010001/2021_EPI_ISL_3459078_2021-06-30<br>France/GES-HMN-21112050571/2021_EPI_ISL_6250920_2021-10-29<br>France/GES-HMN-21112190219/2021_EPI_ISL_7213624_2021-11-05<br>France/IDF-CERBAHC-10315989/2021_EPI_ISL_6261337_2021-10-27<br>France/IDF-HMN-21112090420/2021_EPI_ISL_6436900_2021-10-26<br>France/IDF-HMN-21112160414/2021_EPI_ISL_7289904_2021-10-29<br>France/IDF-IPP32538/2021_EPI_ISL_6424932_2021-10-26<br>France/IDF-IPP33696/2021_EPI_ISL_6699391_2021-10-28<br>France/NAQ-HCL021154029801/2021_EPI_ISL_4320475_2021-08-24<br>hCoV-19/Guinea/IPG-24116/2021_2021-08-30_2022-02-24<br>hCoV-19/Guinea/IP-GNC_22792/2021_2021-07-16_2021-09-07<br>hCoV-19/Malawi/NHRL-COVseq0114/2021_2021-09-09_2021-11-24<br>hCoV-19/Mozambique/COV-2177-A1824/2021_2021-06-17_2021-11-01<br>Malawi/CERI-KRISP-K024691/2021_EPI_ISL_4474382_2021-07-26<br>Malawi/CERI-KRISP-K024698/2021_EPI_ISL_4474388_2021-07-26<br>Malawi/CERI-KRISP-K027022/2021_EPI_ISL_6422344_2021-09-02<br>Malawi/CERI-KRISP-K027067/2021_EPI_ISL_6511739_2021-09-13<br>Malawi/MLW-00130/2021_EPI_ISL_6963056_2021-06-17<br>Malawi/MLW-00173/2021_EPI_ISL_6963100_2021-08-19<br>Malawi/MLW-00260/2021_EPI_ISL_6963179_2021-08-24<br>Malawi/NHRL-COVseq0015/2021_EPI_ISL_4857733_2021-05-27<br>Mozambique/CERI-KRISP-K021220/2021_EPI_ISL_3663608_2021-07-14<br>Mozambique/CERI-KRISP-K025859/2021_EPI_ISL_5425739_2021-07-07<br>Mozambique/CERI-KRISP-K025888/2021_EPI_ISL_5425757_2021-07-06<br>Mozambique/CERI-KRISP-K025913/2021_EPI_ISL_5425745_2021-06-28<br>Mozambique/CERI-KRISP-K025924/2021_EPI_ISL_5425651_2021-07-01<br>Mozambique/CERI-KRISP-K026093/2021_EPI_ISL_5942733_2021-06-30<br>Mozambique/CERI-KRISP-K026094/2021_EPI_ISL_5942754_2021-06-29<br>Mozambique/CERI-KRISP-K026098/2021_EPI_ISL_5942793_2021-07-02<br>Mozambique/CERI-KRISP-K026113/2021_EPI_ISL_5942762_2021-07-22<br>Mozambique/CERI-KRISP-K026123/2021_EPI_ISL_5942826_2021-07-19<br>Mozambique/CERI-KRISP-K026127/2021_EPI_ISL_5942713_2021-07-20<br>Mozambique/CERI-KRISP-K026128/2021_EPI_ISL_5942665_2021-07-22<br>Mozambique/CERI-KRISP-K026139/2021_EPI_ISL_5942740_2021-07-16<br>Mozambique/CERI-KRISP-K026153/2021_EPI_ISL_5942672_2021-07-14<br>Mozambique/CERI-KRISP-K027169/2021_EPI_ISL_8799658_2021-07-07<br>Mozambique/CERI-KRISP-K027175/2021_EPI_ISL_8799664_2021-07-13<br>Mozambique/CERI-KRISP-K027179/2021_EPI_ISL_8799668_2021-07-23<br>Mozambique/CERI-KRISP-K027183/2021_EPI_ISL_8799671_2021-07-27<br>Mozambique/CERI-KRISP-K027540/2021_EPI_ISL_9084393_2021-09-08<br>Mozambique/CERI-KRISP-K027542/2021_EPI_ISL_9084395_2021-09-08<br>Mozambique/CERI-KRISP-K027976/2021_EPI_ISL_7417469_2021-09-01<br>Mozambique/CERI-KRISP-K027987/2021_EPI_ISL_7417442_2021-09-15<br>Mozambique/CERI-KRISP-K027991/2021_EPI_ISL_7417429_2021-09-14<br>Mozambique/CERI-KRISP-K027993/2021_EPI_ISL_7417243_2021-09-13<br>Mozambique/CERI-KRISP-K030796/2021_EPI_ISL_6795170_2021-09-30<br>Mozambique/CERI-KRISP-K030803/2021_EPI_ISL_6795173_2021-10-18<br>Mozambique/CERI-KRISP-K030807/2021_EPI_ISL_6795175_2021-08-30<br>Mozambique/CERI-KRISP-K030827/2021_EPI_ISL_6795185_2021-09-02<br>Mozambique/CERI-KRISP-K032760/2021_EPI_ISL_7715458_2021-11-27<br>Mozambique_COV035972<br>Mozambique_COV035974<br>Mozambique_COV036014<br>Mozambique_COV036025<br>Mozambique_COV036031<br>Mozambique_COV036032<br>Mozambique_COV036053<br>Mozambique_COV036059<br>Mozambique_COV036090<br>Mozambique_COV036095<br>Mozambique/COV-2084-A1707/2021_EPI_ISL_5822570_2021-06-21<br>Mozambique/COV-2089-A1712/2021_EPI_ISL_5822573_2021-06-21<br>Mozambique/COV-2137-A1784/2021_EPI_ISL_5822696_2021-06-17<br>Mozambique/COV-2175-A1822/2021_EPI_ISL_5822658_2021-06-21<br>Mozambique/COV-2208-A1855/2021_EPI_ISL_5822609_2021-06-21<br>Mozambique/COV-2210-A1857/2021_EPI_ISL_5822662_2021-06-17<br>Mozambique/COV-2221-A1868/2021_EPI_ISL_5822616_2021-06-21<br>Mozambique/COV-2224-A1871/2021_EPI_ISL_5822618_2021-06-21<br>Mozambique/COV-2225-A1872/2021_EPI_ISL_5822657_2021-06-16<br>Mozambique/COV-2226-A1873/2021_EPI_ISL_5822619_2021-06-21<br>Mozambique/COV-2233-A1881/2021_EPI_ISL_5822626_2021-06-17<br>Mozambique/COV-2238-A1886/2021_EPI_ISL_5822631_2021-06-17<br>Mozambique/COV-2243-A1891/2021_EPI_ISL_5822700_2021-06-21<br>MWI/MLW00101/2021_OL779041.1_2021-02-23<br>MWI/MLW00231/2021_OL779161.1_2021-06-22<br>SouthAfrica/CERI-KRISP-K023390/2021_EPI_ISL_4336915_2021-08-25<br>SouthAfrica/CERI-KRISP-K023403/2021_EPI_ISL_4336926_2021-08-25<br>SouthAfrica/CERI-KRISP-K025152/2021_EPI_ISL_4877015_2021-09-14<br>SouthAfrica/KRISP-K020391/2021_EPI_ISL_3261982_2021-07-19<br>SouthAfrica/KRISP-K024947/2021_EPI_ISL_4505655_2021-09-09<br>SouthAfrica/NHLS-UCT-AM-Z060/2021_EPI_ISL_3690639_2021-06-30<br>SouthAfrica/NICD-N16542/2021_EPI_ISL_5196414_2021-08-31<br>USA/CO-CDC-FG-110871/2021_OK106110.1_2021-08-30<br>USA/TX-CDC-STM-000731165/2021_OL528706.1_2021-09-10<br>USA/WY-WYPHL-21065673/2021_EPI_ISL_5405564_2021-08-24<br>USA/WY-WYPHL-21068071/2021_EPI_ISL_4440778_2021-08-27<br>France/IDF-HMN-21112180284/2021_EPI_ISL_6781557_2021-11-02<br>Mozambique/CERI-KRISP-K025934/2021_EPI_ISL_5425737_2021-07-10<br>Mozambique/CERI-KRISP-K026110/2021_EPI_ISL_5942761_2021-07-19<br>Mozambique/CERI-KRISP-K027155/2021_EPI_ISL_8799652_2021-07-20<br>Mozambique/CERI-KRISP-K027956/2021_EPI_ISL_7417212_2021-09-17<br>Mozambique/CERI-KRISP-K027963/2021_EPI_ISL_7417415_2021-09-08<br>Mozambique_COV036056	Samples with dates on February were not considered, since those dates disagree with the reported time of Delta wave

G2	39	64	5	60.94	12.82	UK	before 2021-06-16	AY.6	DRC/INRB-RDC-598/2021_EPI_ISL_9570873_2021<br>France/BRE-IPP22834/2021_EPI_ISL_3999704_2021-08-10<br>Guinea/LFHVG-G0426/2021_EPI_ISL_9569771_2021-07-16<br>hCoV-19/Guinea/Conakry_22539/2021_2021-07-06_2021-09-07<br>hCoV-19/Guinea/Conakry_22551/2021_2021-07-07_2021-09-07<br>hCoV-19/Mozambique/CERI-KRISP-K021145/2021_2021-07-07_2021-08-25<br>hCoV-19/Mozambique/COV-2098-A1721/2021_2021-06-17_2021-11-01<br>Mozambique/CERI-KRISP-K025844/2021_EPI_ISL_5425637_2021-07-07<br>Mozambique/CERI-KRISP-K025862/2021_EPI_ISL_5425734_2021-08-07<br>Mozambique/CERI-KRISP-K025867/2021_EPI_ISL_5425815_2021-07-10<br>Mozambique/CERI-KRISP-K025915/2021_EPI_ISL_5425719_2021-06-28<br>Mozambique/CERI-KRISP-K025928/2021_EPI_ISL_5425808_2021-07-02<br>Mozambique/CERI-KRISP-K025937/2021_EPI_ISL_5425763_2021-06-25<br>Mozambique/CERI-KRISP-K026088/2021_EPI_ISL_5942839_2021-07-02<br>Mozambique/CERI-KRISP-K026155/2021_EPI_ISL_5942816_2021-07-16<br>Mozambique/CERI-KRISP-K027545/2021_EPI_ISL_9084398_2021-09-09<br>Mozambique/CERI-KRISP-K027960/2021_EPI_ISL_7417021_2021-09-08<br>Mozambique/CERI-KRISP-K027968/2021_EPI_ISL_7417044_2021-09-27<br>Mozambique/CERI-KRISP-K027979/2021_EPI_ISL_7417051_2021-09-08<br>Mozambique/CERI-KRISP-K027981/2021_EPI_ISL_7417365_2021-09-03<br>Mozambique_COV036018<br>Mozambique_COV036058<br>Mozambique_COV036067<br>Mozambique_COV036088<br>Mozambique_COV036112<br>Mozambique/COV-2083-A1706/2021_EPI_ISL_5822569_2021-06-21<br>Mozambique/COV-2093-A1716/2021_EPI_ISL_5822576_2021-06-21<br>Mozambique/COV-2155-A1802/2021_EPI_ISL_5822666_2021-05-04<br>Mozambique/COV-2165-A1812/2021_EPI_ISL_5822590_2021-06-21<br>Mozambique/COV-2180-A1827/2021_EPI_ISL_5822597_2021-06-16<br>Mozambique/COV-2192-A1839/2021_EPI_ISL_5822602_2021-06-21<br>Mozambique/COV-2193-A1840/2021_EPI_ISL_5822603_2021-06-17<br>Mozambique/COV-2200-A1847/2021_EPI_ISL_5822605_2021-06-21<br>Mozambique/COV-2205-A1852/2021_EPI_ISL_5822607_2021-06-17<br>Mozambique/COV-2209-A1856/2021_EPI_ISL_5822610_2021-06-20<br>Mozambique/COV-2217-A1864/2021_EPI_ISL_5822614_2021-06-21<br>Mozambique/COV-2223-A1870/2021_EPI_ISL_5822617_2021-06-21<br>Mozambique/COV-2231-A1879/2021_EPI_ISL_5822624_2021-06-18<br>Mozambique/COV-2232-A1880/2021_EPI_ISL_5822625_2021-06-21<br>Mozambique/COV-2239-A1887/2021_EPI_ISL_5822632_2021-06-17<br>Mozambique/COV-2242-A1890/2021_EPI_ISL_5822672_2021-06-21<br>Mozambique/COV-2257-A1905/2021_EPI_ISL_5822639_2021-06-25<br>SouthAfrica/CERI-KRISP-K025252/2021_EPI_ISL_5098720_2021-09-13<br>SouthAfrica/CERI-KRISP-K025256/2021_EPI_ISL_5098853_2021-09-13<br>SouthAfrica/CERI-KRISP-Tyg1734/2021_EPI_ISL_3827608_2021-07-27<br>SouthAfrica/CERI-KRISP-Tyg1819/2021_EPI_ISL_3827731_2021-07-28<br>SouthAfrica/KRISP-K022376/2021_EPI_ISL_3939076_2021-08-13<br>SouthAfrica/NHLS-UCT-GS-AE75/2021_EPI_ISL_6436444_2021-10-22<br>SouthAfrica/NICD-N11985/2021_EPI_ISL_3451123_2021-06-28<br>SouthAfrica/NICD-N14306/2021_EPI_ISL_4651026_2021-07-27<br>SouthAfrica/NICD-N15584/2021_EPI_ISL_4652360_2021-08-25<br>SouthAfrica/NICD-N18514/2021_EPI_ISL_6201695_2021-09-14<br>SouthAfrica/NICD-N21351/2021_EPI_ISL_7456411_2021-11-08<br>SouthAfrica/NICD-R11724/2021_EPI_ISL_3644037_2021-08-03<br>SouthAfrica/NICD-R13126/2021_EPI_ISL_4891862_2021-09-02<br>SouthAfrica/Tygerberg_2465/2021_EPI_ISL_4250506_2021-07-07<br>SouthAfrica/Tygerberg_2716/2021_EPI_ISL_4750281_2021-09-13<br>Guinea/LFHVG-G0428/2021_EPI_ISL_9569773_2021-07-16<br>Mozambique/CERI-KRISP-K030806/2021_EPI_ISL_6795174_2021-08-14<br>Mozambique/COV-2194-A1841/2021_EPI_ISL_5822665_2021-06-21<br>SouthAfrica/KRISP-UFS-K022171/2021_EPI_ISL_4003500_2021-07-11<br>SouthAfrica/NICD-N17111/2021_EPI_ISL_5477785_2021-07-27<br>SouthAfrica/NICD-N18041/2021_EPI_ISL_5918126_2021-09-11<br>SouthAfrica/NICD-R11988/2021_EPI_ISL_3644064_2021-08-12	

G3	34	35	18	97.14	52.94	undetermined	before 2021-06-29	AY.122	Mozambique/CERI-KRISP-K021121/2021_EPI_ISL_3663551_2021-07-02<br>Mozambique/CERI-KRISP-K021126/2021_EPI_ISL_3663556_2021-07-01<br>Mozambique/CERI-KRISP-K021147/2021_EPI_ISL_3663560_2021-07-06<br>Mozambique/CERI-KRISP-K021153/2021_EPI_ISL_3663563_2021-07-10<br>Mozambique/CERI-KRISP-K021196/2021_EPI_ISL_3663593_2021-07-14<br>Mozambique/CERI-KRISP-K021202/2021_EPI_ISL_3663599_2021-07-12<br>Mozambique/CERI-KRISP-K021211/2021_EPI_ISL_3663605_2021-07-16<br>Mozambique/CERI-KRISP-K025868/2021_EPI_ISL_5425736_2021-07-10<br>Mozambique/CERI-KRISP-K026101/2021_EPI_ISL_5942764_2021-07-02<br>Mozambique/CERI-KRISP-K026102/2021_EPI_ISL_5942685_2021-07-26<br>Mozambique/CERI-KRISP-K026103/2021_EPI_ISL_5942675_2021-06-29<br>Mozambique/CERI-KRISP-K026129/2021_EPI_ISL_5942669_2021-07-16<br>Mozambique/CERI-KRISP-K027543/2021_EPI_ISL_9084396_2021-09-09<br>Mozambique/CERI-KRISP-K027962/2021_EPI_ISL_7417328_2021-09-09<br>Mozambique_COV035927<br>Mozambique_COV035934<br>Mozambique_COV035935<br>Mozambique_COV035939<br>Mozambique_COV035943<br>Mozambique_COV035951<br>Mozambique_COV035956<br>Mozambique_COV035988<br>Mozambique_COV035999<br>Mozambique_COV036002<br>Mozambique_COV036030<br>Mozambique_COV036043<br>Mozambique_COV036045<br>Mozambique_COV036074<br>Mozambique_COV036075<br>Mozambique_COV036076<br>Mozambique_COV036113<br>SouthAfrica/NICD-N16720/2021_EPI_ISL_5416281_2021-08-02<br>Mozambique/CERI-KRISP-K021122/2021_EPI_ISL_3663552_2021-07-02<br>Mozambique/CERI-KRISP-K021203/2021_EPI_ISL_3663600_2021-07-14<br>Mozambique_COV035942	
G4	26	34	1	76.47	3.85	UK	before 2021-06-16	AY.6	Mozambique/CERI-KRISP-K025846/2021_EPI_ISL_5425755_2021-07-07<br>Mozambique/CERI-KRISP-K025849/2021_EPI_ISL_5425761_2021-07-07<br>Mozambique/CERI-KRISP-K025850/2021_EPI_ISL_5425724_2021-07-07<br>Mozambique/CERI-KRISP-K025860/2021_EPI_ISL_5425687_2021-08-07<br>Mozambique/CERI-KRISP-K025863/2021_EPI_ISL_5425738_2021-08-07<br>Mozambique/CERI-KRISP-K025890/2021_EPI_ISL_5425735_2021-07-06<br>Mozambique/CERI-KRISP-K025895/2021_EPI_ISL_5425784_2021-07-07<br>Mozambique/CERI-KRISP-K026096/2021_EPI_ISL_5942709_2021-06-29<br>Mozambique/CERI-KRISP-K026116/2021_EPI_ISL_5942666_2021-07-19<br>Mozambique/CERI-KRISP-K026134/2021_EPI_ISL_5942798_2021-07-17<br>Mozambique/CERI-KRISP-K027140/2021_EPI_ISL_8799647_2021-07-27<br>Mozambique/CERI-KRISP-K027167/2021_EPI_ISL_8799656_2021-07-01<br>Mozambique/CERI-KRISP-K027532/2021_EPI_ISL_9084390_2021-08-07<br>Mozambique/CERI-KRISP-K030812/2021_EPI_ISL_6795178_2021-09-20<br>Mozambique/CERI-KRISP-K030823/2021_EPI_ISL_6795182_2021-08-31<br>Mozambique/CERI-KRISP-K032761/2021_EPI_ISL_7715459_2021-11-04<br>Mozambique_COV036111<br>Mozambique/COV-2270-A2130/2021_EPI_ISL_5822678_2021-06-17<br>Mozambique/COV-2276-A1924/2021_EPI_ISL_5822692_2021-06-24<br>Mozambique/COV-2305-A2131/2021_EPI_ISL_5822681_2021-06-16<br>Mozambique/COV-2318-A1966/2021_EPI_ISL_5822670_2021-06-16<br>Mozambique/COV-2382-A2031/2021_EPI_ISL_5822679_2021-06-16<br>Portugal/PT11406/2021_EPI_ISL_2989327_2021-07-06<br>Portugal/PT11407/2021_EPI_ISL_2988994_2021-07-06<br>SouthAfrica/KRISP-K025069/2021_EPI_ISL_4572346_2021-09-10<br>SouthAfrica/NICD-N20407/2021_EPI_ISL_6327803_2021-10-15<br>SouthAfrica/NICD-N22255/2021_EPI_ISL_7605578_2021-11-27<br>SouthAfrica/NICD-N25253/2021_EPI_ISL_8799219_2021-12-13<br>Spain/GA-CHUVI-34178039/2021_EPI_ISL_4273610_2021-08-23<br>USA/TX-CDC-LC0104101/2021_MZ787485.1_2021-07-06<br>Mozambique/CERI-KRISP-K021195/2021_EPI_ISL_3663592_2021-07-14<br>Mozambique/CERI-KRISP-K025907/2021_EPI_ISL_5425787_2021-06-30<br>Mozambique/CERI-KRISP-K030808/2021_EPI_ISL_6795176_2021-09-01<br>Mozambique/COV-2249-A1897/2021_EPI_ISL_5822635_2021-06-18	

G5	9	15	4	60.00	44.44	India	2021-04-26 ; 2021-06-18	AY.122	Canada/AB-ABPHL-27710/2021_EPI_ISL_4053167_2021-08-07 Germany/SN-RKI-I-200017/2021_EPI_ISL_3499658_2021-07-25 IMS-10023-CVDP-A5996BAB-590F-4EC1-A780-DB6741DCCC24_OU570326.1_2021-07-25 Mozambique/CERI-KRISP-K021198/2021_EPI_ISL_3663595_2021-07-13 Mozambique/CERI-KRISP-K026121/2021_EPI_ISL_5942746_2021-07-20 Mozambique/CERI-KRISP-K027996/2021_EPI_ISL_7417402_2021-10-06 Mozambique_COV035922 Mozambique_COV035995 Mozambique_COV036005 Mozambique_COV036080 Mozambique/COV-2274-A1922/2021_EPI_ISL_5822646_2021-06-18 SouthAfrica/NICD-N13205/2021_EPI_ISL_3643946_2021-07-15 SouthAfrica/NICD-N14396/2021_EPI_ISL_4651279_2021-07-12 England/QEUH-18D197B/2021_OU437211.1_2021-07-08 Mozambique/CERI-KRISP-K027971/2021_EPI_ISL_7417145_2021-09-25	
G6	13	14	0	92.86	0.00	UK	before 2021-07-05	AY.6	Mozambique/CERI-KRISP-K027192/2021_EPI_ISL_8799677_2021-07-14 Mozambique/CERI-KRISP-K027185/2021_EPI_ISL_8799673_2021-07-27 Mozambique/CERI-KRISP-K027184/2021_EPI_ISL_8799672_2021-07-26 Malawi/CERI-KRISP-K027104/2021_EPI_ISL_6511749_2021-09-10 Mozambique/CERI-KRISP-K027206/2021_EPI_ISL_8799681_2021-07-14 Mozambique/CERI-KRISP-K027195/2021_EPI_ISL_8799678_2021-07-14 Mozambique/CERI-KRISP-K027171/2021_EPI_ISL_8799660_2021-07-12 Mozambique/CERI-KRISP-K027216/2021_EPI_ISL_8799683_2021-07-12 Mozambique/CERI-KRISP-K027160/2021_EPI_ISL_8799655_2021-07-20 Mozambique/CERI-KRISP-K027173/2021_EPI_ISL_8799662_2021-07-13 Mozambique/CERI-KRISP-K027191/2021_EPI_ISL_8799676_2021-07-14 Mozambique/CERI-KRISP-K027168/2021_EPI_ISL_8799657_2021-07-05 Mozambique/CERI-KRISP-K027170/2021_EPI_ISL_8799659_2021-07-12 Mozambique/CERI-KRISP-K027214/2021_EPI_ISL_8799682_2021-07-12	
G7	14	14	1	100.00	7.14	UK	before 2021-06-17	AY.6	Mozambique/COV-2229-A1877/2021_EPI_ISL_5822622_2021-06-21 Mozambique/COV-2235-A1883/2021_EPI_ISL_5822628_2021-06-21 Mozambique/COV-2241-A1889/2021_EPI_ISL_5822633_2021-06-21 Mozambique/CERI-KRISP-K027131/2021_EPI_ISL_6511763_2021-07-27 Mozambique/CERI-KRISP-K027126/2021_EPI_ISL_6511758_2021-07-26 Mozambique_COV036069 hCoV-19/Mozambique/CERI-KRISP-K025848/2021_2021-07-07_2021-10-22 hCoV-19/Mozambique/CERI-KRISP-K025938/2021_2021-06-28_2021-10-22 Mozambique/COV-2135-A1782/2021_EPI_ISL_5822584_2021-06-22 hCoV-19/Mozambique/COV-2087-A1710/2021_2021-06-21_2021-11-01 Mozambique/COV-2099-A1722/2021_EPI_ISL_5822581_2021-06-21 Mozambique/COV-2138-A1785/2021_EPI_ISL_5822587_2021-06-22 Mozambique/COV-2228-A1876/2021_EPI_ISL_5822621_2021-06-21 Mozambique/COV-2236-A1884/2021_EPI_ISL_5822629_2021-06-17	
G8	8	9	5	88.89	62.50	undetermined	before 2021-08-09	AY.122	hCoV-19/Mozambique/CERI-KRISP-K021192/2021_2021-07-08_2021-08-25 hCoV-19/Mozambique/CERI-KRISP-K027546/2021_2021-07-20_2022-01-25 Mozambique_COV035931 Mozambique_COV036011 Mozambique_COV036008 Mozambique_COV035961 Mozambique_COV035955 hCoV-19/Mozambique/COV-2261-A1909/2021_2021-06-24_2021-11-01 hCoV-19/Portugal/PT13248/2021_2021-07-26_2021-08-09	
G9	7	7	0	100.00	0.00	undetermined	before 2021-08-31	AY.122	Mozambique/CERI-KRISP-K030802/2021_EPI_ISL_6795172_2021-10-18 Mozambique/CERI-KRISP-K030824/2021_EPI_ISL_6795183_2021-08-31 Mozambique/CERI-KRISP-K030833/2021_EPI_ISL_6795187_2021-09-01 Mozambique/CERI-KRISP-K030828/2021_EPI_ISL_6795186_2021-09-02 hCoV-19/Mozambique/CERI-KRISP-K025886/2021_2021-07-09_2021-10-22 hCoV-19/Mozambique/CERI-KRISP-K025870/2021_2021-07-10_2021-10-22 hCoV-19/Mozambique/CERI-KRISP-K025884/2021_2021-07-08_2021-10-22	
G10	7	7	0	100.00	0.00	UK	before 2021-07-16	AY.6	Mozambique/CERI-KRISP-K027186/2021_EPI_ISL_8799674_2021-07-30 Mozambique/CERI-KRISP-K027182/2021_EPI_ISL_8799670_2021-07-20 Mozambique/CERI-KRISP-K027180/2021_EPI_ISL_8799669_2021-07-23 Mozambique/CERI-KRISP-K027177/2021_EPI_ISL_8799666_2021-07-19 Mozambique/CERI-KRISP-K027174/2021_EPI_ISL_8799663_2021-07-16 Mozambique/CERI-KRISP-K027541/2021_EPI_ISL_9084394_2021-09-08 Mozambique/CERI-KRISP-K027964/2021_EPI_ISL_7417032_2021-09-08	
G11	5	7	0	71.43	0.00	UK	before 2021-06-21	AY.6	Mozambique/COV-2204-A1851/2021_EPI_ISL_5822606_2021-06-21 Mozambique/CERI-KRISP-K025911/2021_EPI_ISL_5425638_2021-07-01 SouthAfrica/NICD-N15597/2021_EPI_ISL_4651407_2021-08-18 Mozambique/CERI-KRISP-K026163/2021_EPI_ISL_5942670_2021-07-30 Mozambique/CERI-KRISP-K026157/2021_EPI_ISL_5942751_2021-07-22 SouthAfrica/NICD-N13798/2021_EPI_ISL_6202812_2021-07-28 hCoV-19/Mozambique/CERI-KRISP-K025892/2021_2021-07-07_2021-10-22	
G12	6	6	1	100.00	16.67	UK	before 2021-05-24	AY.6	Mozambique/CERI-KRISP-K026111/2021_EPI_ISL_5942809_2021-07-19 Mozambique/COV-2359-A2008/2021_EPI_ISL_5822652_2021-05-24 hCoV-19/Mozambique/CERI-KRISP-K021200/2021_2021-07-13_2021-08-25 hCoV-19/Mozambique/CERI-KRISP-K021157/2021_2021-07-09_2021-08-25 hCoV-19/Mozambique/CERI-KRISP-K021166/2021_2021-07-12_2021-08-25 Mozambique_COV035960	
G13	3	3	0	100.00	0.00	India	2021-03-23 ; 2021-06-05	B.1.617.2	Mozambique/COV-2256-A1904/2021_EPI_ISL_5822638_2021-06-22 Mozambique/COV-2234-A1882/2021_EPI_ISL_5822627_2021-06-21 Mozambique/COV-2197-A1844/2021_EPI_ISL_5822604_2021-06-21	

G14	5	5	0	100.00	0.00	undetermined	before 2021-05-20	B.1.617.2	Mozambique/COV-2302-A1950/2021_EPI_ISL_5822654_2021-05-20 Mozambique/COV-2280-A1928/2021_EPI_ISL_5822648_2021-06-24 Mozambique/COV-2272-A1920/2021_EPI_ISL_5822645_2021-06-17 hCoV-19/Mozambique/COV-2279-A1927/2021_2021-06-24_2021-11-01 hCoV-19/Mozambique/CERI-KRISP-K021162/2021_2021-07-09_2021-08-25	
G15	5	5	5	100.00	100.00	SouthAfrica	2021-06-17 ; 2021-07-08	B.1.617.2	Mozambique_COV035968 Mozambique_COV036028 Mozambique_COV036051 Mozambique_COV036052 Mozambique_COV035969	
G16	5	5	0	100.00	0.00	undetermined	before 2021-10-22	AY.122	hCoV-19/Mozambique/CERI-KRISP-K025894/2021_2021-07-06_2021-10-22 hCoV-19/Mozambique/CERI-KRISP-K025877/2021_2021-07-08_2021-10-22 hCoV-19/Mozambique/CERI-KRISP-K026108/2021_2021-07-10_2021-11-05 hCoV-19/Mozambique/CERI-KRISP-K026130/2021_2021-07-17_2021-11-05 hCoV-19/Mozambique/CERI-KRISP-K026135/2021_2021-07-19_2021-11-05	
G17	3	3	3	100.00	100.00	SouthAfrica	before 2021-06-30	AY.45	Mozambique_COV036101 Mozambique_COV036057 Mozambique_COV036083	
G18	5	5	0	100.00	0.00	UK	before 2021-06-28	AY.6	Mozambique/CERI-KRISP-K025939/2021_EPI_ISL_5425762_2021-06-29 Mozambique/CERI-KRISP-K025925/2021_EPI_ISL_5425786_2021-07-01 Mozambique/CERI-KRISP-K025930/2021_EPI_ISL_5425780_2021-06-28 Mozambique/CERI-KRISP-K025864/2021_EPI_ISL_5425778_2021-08-07 Mozambique/CERI-KRISP-K026141/2021_EPI_ISL_5942760_2021-07-16	
G19	3	4	1	75.00	33.33	undetermined	before 2021-07-02	AY.122	Mozambique/CERI-KRISP-K026100/2021_EPI_ISL_5942714_2021-07-02 Mozambique/CERI-KRISP-K026106/2021_EPI_ISL_5942673_2021-07-09 SouthAfrica/CERI-KRISP-K025336/2021_EPI_ISL_5099155_2021-09-06 Mozambique_COV035938	
G20	2	2	0	100.00	0.00	Germany	2021-07-03 ; 2021-07-09	AY.122	Mozambique/CERI-KRISP-K021163/2021_EPI_ISL_3663572_2021-07-09 Mozambique/CERI-KRISP-K021160/2021_EPI_ISL_3663570_2021-07-09	
G21	3	3	3	100.00	100.00	SouthAfrica	before 2021-07-01	AY.45	Mozambique_COV035973 Mozambique_COV036078 Mozambique_COV036027	
G22	2	3	0	66.67	0.00	SouthAfrica	2021-06-21 ; 2021-06-29	AY.45	Mozambique/CERI-KRISP-K021204/2021_EPI_ISL_3663601_2021-07-07 Mozambique/CERI-KRISP-K021124/2021_EPI_ISL_3663554_2021-06-29 Canada/AB-ABPHL-27711/2021_EPI_ISL_4053168_2021-08-05	
G23	3	3	2	100.00	66.67	SouthAfrica	before 2021-06-24	AY.45	Mozambique/COV-2262-A1910/2021_EPI_ISL_5822643_2021-06-24 Mozambique_COV035963 Mozambique_COV035964	
G24	3	3	3	100.00	100.00	SouthAfrica	before 2021-07-30	AY.45	Mozambique_COV036040 Mozambique_COV036041 Mozambique_COV036042	
G25	3	3	3	100.00	100.00	SouthAfrica	before 2021-07-05	AY.45	Mozambique_COV036006 Mozambique_COV036073 Mozambique_COV036055	
G26	3	3	0	100.00	0.00	undetermined	before 2021-07-19	AY.116	hCoV-19/Mozambique/CERI-KRISP-K026154/2021_2021-07-19_2021-11-05 Mozambique/CERI-KRISP-K026161/2021_EPI_ISL_5942745_2021-07-20 hCoV-19/Mozambique/CERI-KRISP-K026164/2021_2021-07-31_2021-11-05	
G27	2	2	0	100.00	0.00	SouthAfrica	2021-07-12 ; 2021-07-19	AY.6	Mozambique/CERI-KRISP-K026160/2021_EPI_ISL_5942712_2021-07-20 Mozambique/CERI-KRISP-K026149/2021_EPI_ISL_5942711_2021-07-19	
G28	2	2	0	100.00	0.00	UK	2021-06-30 ; 2021-07-10	AY.6	Mozambique/CERI-KRISP-K025885/2021_EPI_ISL_5425772_2021-07-10 Mozambique/CERI-KRISP-K025874/2021_EPI_ISL_5425726_2021-07-10	
G29	2	2	0	100.00	0.00	UK	2021-07-09 ; 2021-07-26	AY.6	Mozambique/CERI-KRISP-K027127/2021_EPI_ISL_6511759_2021-07-26 Mozambique/CERI-KRISP-K027124/2021_EPI_ISL_6511756_2021-07-26	
G30	3	3	0	100.00	0.00	India	2021-08-26 ; 2021-09-06	AY.6	Mozambique/CERI-KRISP-K030815/2021_EPI_ISL_6795180_2021-09-29 Mozambique/CERI-KRISP-K030814/2021_EPI_ISL_6795179_2021-09-21 Mozambique/CERI-KRISP-K030810/2021_EPI_ISL_6795177_2021-09-06	
G31	3	3	0	100.00	0.00	UK	before 2021-10-22	AY.6	hCoV-19/Mozambique/CERI-KRISP-K025854/2021_2021-07-07_2021-10-22 hCoV-19/Mozambique/CERI-KRISP-K025879/2021_2021-07-08_2021-10-22 hCoV-19/Mozambique/CERI-KRISP-K025856/2021_2021-07-07_2021-10-22	
G32	2	2	2	100.00	100.00	UK	2021-05-01 ; 2021-07-01	AY.6	Mozambique_COV035983 Mozambique_COV035976	
G33	2	2	0	100.00	0.00	Malawi	2021-06-24 ; 2021-07-15	B.1.617.2	Mozambique/CERI-KRISP-K021221/2021_EPI_ISL_3663609_2021-07-15 Mozambique/CERI-KRISP-K021214/2021_EPI_ISL_3663607_2021-07-14	
G34	2	2	0	100.00	0.00	undetermined	before 2021-07-02	AY.122	Mozambique/CERI-KRISP-K026136/2021_EPI_ISL_5942728_2021-07-21 Mozambique/CERI-KRISP-K026104/2021_EPI_ISL_5942720_2021-07-02	
G35	2	2	2	100.00	100.00	undetermined	before 2021-06-21	AY.122	Mozambique_COV036104 Mozambique_COV035966	
G36	2	2	2	100.00	100.00	UK	2021-07-01 ; 2021-07-19	AY.122	Mozambique_COV036020 Mozambique_COV035928	
G37	2	2	0	100.00	0.00	undetermined	before 2021-07-09	AY.122	Mozambique/CERI-KRISP-K021179/2021_EPI_ISL_3663584_2021-07-12 Mozambique/CERI-KRISP-K021168/2021_EPI_ISL_3663577_2021-07-09	
G38	2	2	0	100.00	0.00	SouthAfrica	2021-07-22 ; 2021-08-08	AY.122	Mozambique/CERI-KRISP-K027538/2021_EPI_ISL_9084392_2021-08-08 Mozambique/CERI-KRISP-K027972/2021_EPI_ISL_7417340_2021-09-25	
G39	2	2	0	100.00	0.00	undetermined	before 2021-08-25	AY.122	hCoV-19/Mozambique/CERI-KRISP-K021174/2021_2021-07-09_2021-08-25 hCoV-19/Mozambique/CERI-KRISP-K021186/2021_2021-07-14_2021-08-25	
G40	2	2	1	100.00	50.00	undetermined	before 2021-07-09	AY.122	Mozambique_COV035992 Mozambique/CERI-KRISP-K021152/2021_EPI_ISL_3663562_2021-07-09	
G41	2	2	1	100.00	50.00	SouthAfrica	2021-07-11 ; 2021-07-15	AY.45	Mozambique_COV036001 Mozambique/CERI-KRISP-K021224/2021_EPI_ISL_3663610_2021-07-15	
G42	2	2	0	100.00	0.00	SouthAfrica	before 2021-07-14	AY.45	hCoV-19/Mozambique/CERI-KRISP-K021210/2021_2021-07-14_2021-08-25 hCoV-19/Mozambique/CERI-KRISP-K021212/2021_2021-07-14_2021-08-25	

G43	2	2	2	100.00	100.00	SouthAfrica	before 2021-07-05	AY.45	Mozambique_COV035940 Mozambique_COV035952	
G44	2	2	2	100.00	100.00	SouthAfrica	before 2021-06-30	AY.45	Mozambique_COV036070 Mozambique_COV036046	
G45	2	2	0	100.00	0.00	UK	before 2021-06-29	AY.6	Mozambique/CERI-KRISP-K025929/2021_EPI_ISL_5425722_2021-06-29 Mozambique/CERI-KRISP-K025881/2021_EPI_ISL_5425754_2021-07-08	
G46	2	2	0	100.00	0.00	UK	before 2021-06-24	AY.6	Mozambique/CERI-KRISP-K026147/2021_EPI_ISL_5942763_2021-07-16 Mozambique/CERI-KRISP-K025936/2021_EPI_ISL_5425800_2021-06-24	
G47	2	2	0	100.00	0.00	UK/Wales	2021-08-10;2021-09-08	AY.6	Mozambique/CERI-KRISP-K027544/2021_EPI_ISL_9084397_2021-09-08 Mozambique/CERI-KRISP-K027961/2021_EPI_ISL_7417274_2021-09-09	
G48	2	2	0	100.00	0.00	undetermined	before 2021-06-28	AY.116	Mozambique/CERI-KRISP-K025917/2021_EPI_ISL_5425740_2021-06-28 Mozambique/CERI-KRISP-K025883/2021_EPI_ISL_5425788_2021-07-12	
G49	2	2	0	100.00	0.00	India	2021-05-13 ; 2021-07-14	undetermined	Mozambique/CERI-KRISP-K027159/2021_EPI_ISL_8799654_2021-08-07 Mozambique/CERI-KRISP-K027205/2021_EPI_ISL_8799680_2021-07-14	
**total_sampl**	**324**		**78**							

Delta unique sequences			
**Strain**	**Likely_geographical_origin**	**Likely_introduction_period**	**new_seq**
hCoV-19/Mozambique/CERI-KRISP-K021115/2021_2021-06-28_2021-08-25	India	2021-04-05;2021-06-28	no
hCoV-19/Mozambique/CERI-KRISP-K021117/2021_2021-06-30_2021-08-25	India	2021-04-05;2021-06-30	no
hCoV-19/Mozambique/CERI-KRISP-K021118/2021_2021-06-30_2021-08-25	India	2021-04-05;2021-06-30	no
hCoV-19/Mozambique/CERI-KRISP-K021119/2021_2021-06-30_2021-08-25	India	2021-04-19;2021-06-30	no
hCoV-19/Mozambique/CERI-KRISP-K021125/2021_2021-07-01_2021-08-25	India	2021-04-05;2021-07-01	no
hCoV-19/Mozambique/CERI-KRISP-K021167/2021_2021-07-12_2021-08-25	India	2021-06-11;2021-07-12	no
hCoV-19/Mozambique/CERI-KRISP-K021169/2021_2021-07-08_2021-08-25	India	2021-04-05;2021-07-08	no
hCoV-19/Mozambique/CERI-KRISP-K021175/2021_2021-07-12_2021-08-25	India	2021-04-30;2021-08-25	no
hCoV-19/Mozambique/CERI-KRISP-K021199/2021_2021-07-14_2021-08-25	India	2021-05-14;2021-08-25	no
hCoV-19/Mozambique/CERI-KRISP-K021205/2021_2021-07-15_2021-08-25	UK	2021-05-01;2021-07-15	no
hCoV-19/Mozambique/CERI-KRISP-K025876/2021_2021-07-09_2021-10-22	India	2021-04-30;2021-07-09	no
hCoV-19/Mozambique/CERI-KRISP-K025896/2021_2021-07-09_2021-10-22	India	2021-05-04;2021-07-14	no
hCoV-19/Mozambique/CERI-KRISP-K025904/2021_2021-07-02_2021-10-22	India	2021-04-05;2021-07-02	no
hCoV-19/Mozambique/CERI-KRISP-K025931/2021_2021-07-02_2021-10-22	undetermined	Before 2021-07-02	no
hCoV-19/Mozambique/CERI-KRISP-K026083/2021_2021-07-03_2021-11-05	undetermined	Before 2021-07-03	no
hCoV-19/Mozambique/CERI-KRISP-K026118/2021_2021-07-16_2021-11-05	undetermined/South Africa	Before 2021-07-16	no
hCoV-19/Mozambique/CERI-KRISP-K026124/2021_2021-07-19_2021-11-05	undetermined	Before 2021-07-19	no
hCoV-19/Mozambique/CERI-KRISP-K026133/2021_2021-07-20_2021-11-05	undetermined	Before 2021-07-20	no
hCoV-19/Mozambique/CERI-KRISP-K026144/2021_2021-07-15_2021-11-05	USA	2021-07-10;2021-07-15	no
hCoV-19/Mozambique/CERI-KRISP-K027529/2021_2021-08-02_2022-01-25	India	2021-04-12;2021-08-02	no
hCoV-19/Mozambique/CERI-KRISP-K027973/2021_2021-09-08_2021-12-09	undetermined	Before 2021-09-08	no
hCoV-19/Mozambique/CERI-KRISP-K027980/2021_2021-09-02_2021-12-09	UK	2021-05-01;2021-09-02	no
hCoV-19/Mozambique/CERI-KRISP-K030798/2021_2021-10-07_2021-11-27	South Africa	2021-09-04;2021-10-07	no
Mozambique_COV035923	undetermined	Before 2021-06-14	**yes**
Mozambique_COV035924	South Africa	2021-06-29;2021-07-07	**yes**
Mozambique_COV035925	undetermined/South Africa	Before 2021-06-28	**yes**
Mozambique_COV035926	South Africa	2021-05-26;2021-07-26	**yes**

Mozambique_COV035929	undetermined	Before 2021-08-12	**yes**
Mozambique_COV035937	South Africa	2021-06-15;2021-06-28	**yes**
Mozambique_COV035950	undetermined/South Africa	Before 2021-07-09	**yes**
Mozambique_COV035953	undetermined	Before 2021-07-09	**yes**
Mozambique_COV035957	UK	2021-07-01;2021-07-08	**yes**
Mozambique_COV035958	undetermined	Before 2021-07-08	**yes**
Mozambique_COV035959	undetermined	Before 2021-07-08	**yes**
Mozambique_COV035962	undetermined/South Africa	Before 2021-10-07	**yes**
Mozambique_COV035965	undetermined/South Africa	Before 2021-08-09	**yes**
Mozambique_COV035967	undetermined	Before 2021-08-10	**yes**
Mozambique_COV035984	undetermined	Before 2021-07-01	**yes**
Mozambique_COV035986	UK	2021-07-01;2021-07-02	**yes**
Mozambique_COV035991	UK	2021-05-01;2021-07-13	**yes**
Mozambique_COV035993	UK	2021-07-01;2021-07-27	**yes**
Mozambique_COV035997	undetermined	Before 2021-07-13	**yes**
Mozambique_COV036007	undetermined	Before 2021-07-19	**yes**
Mozambique_COV036010	undetermined	Before 2021-07-12	**yes**
Mozambique_COV036013	South Africa	2021-06-17;2021-07-14	**yes**
Mozambique_COV036016	undetermined	Before 2021-07-19	**yes**
Mozambique_COV036017	undetermined	Before 2021-07-13	**yes**
Mozambique_COV036022	undetermined	Before 2021-08-11	**yes**
Mozambique_COV036024	Portugal	2021-07-02;2021-07-22	**yes**
Mozambique_COV036029	undetermined	Before 2021-06-30	**yes**
Mozambique_COV036035	undetermined	Before 2021-08-11	**yes**
Mozambique_COV036037	undetermined	Before 2021-08-12	**yes**
Mozambique_COV036044	undetermined	Before 2021-07-30	**yes**
Mozambique_COV036049	undetermined/South Africa	Before 2021-07-31	**yes**
Mozambique_COV036050	undetermined	Before 2021-07-31	**yes**
Mozambique_COV036061	undetermined/South Africa	Before 2021-06-30	**yes**
Mozambique_COV036081	undetermined	Before 2021-07-22	**yes**

Mozambique_COV036082	undetermined	Before 2021-07-13	**yes**
Mozambique_COV036084	undetermined	Before 2021-07-30	**yes**
Mozambique_COV036085	undetermined/South Africa	Before 2021-07-30	**yes**
Mozambique_COV036091	South Africa	2021-05-16;2021-07-12	**yes**
Mozambique_COV036097	undetermined/South Africa	Before 2021-08-31	**yes**
Mozambique_COV036100	India	2021-04-19;2021-07-05	**yes**
Mozambique_COV036109	undetermined	Before 2021-07-27	**yes**
Mozambique_COV036110	undetermined/South Africa	Before 2021-07-27	**yes**
Mozambique/CERI-KRISP-K021127/2021_EPI_ISL_3663557_2021-07-01	India	2021-04-26;2021-07-01	no
Mozambique/CERI-KRISP-K021151/2021_EPI_ISL_3663561_2021-07-06	undetermined	Before 2021-07-06	no
Mozambique/CERI-KRISP-K021155/2021_EPI_ISL_3663565_2021-07-07	India	2021-04-05;2021-07-07	no
Mozambique/CERI-KRISP-K021158/2021_EPI_ISL_3663568_2021-07-09	Bahrain	2021-04-08;2021-07-09	no
Mozambique/CERI-KRISP-K021159/2021_EPI_ISL_3663569_2021-07-09	undetermined/UK	Before 2021-07-09	no
Mozambique/CERI-KRISP-K021164/2021_EPI_ISL_3663573_2021-07-12	undetermined	Before 2021-07-12	no
Mozambique/CERI-KRISP-K021165/2021_EPI_ISL_3663574_2021-07-09	undetermined	Before 2021-07-09	no
Mozambique/CERI-KRISP-K021176/2021_EPI_ISL_3663581_2021-07-13	India	2021-04-26;2021-07-13	no
Mozambique/CERI-KRISP-K021177/2021_EPI_ISL_3663582_2021-07-10	India	2021-04-05;2021-07-10	no
Mozambique/CERI-KRISP-K021178/2021_EPI_ISL_3663583_2021-07-13	India	2021-05-19;2021-07-19	no
Mozambique/CERI-KRISP-K021190/2021_EPI_ISL_3663588_2021-07-08	UK	2021-05-01;2021-07-08	no
Mozambique/CERI-KRISP-K021193/2021_EPI_ISL_3663590_2021-07-13	India	2021-04-19;2021-07-13	no
Mozambique/CERI-KRISP-K021194/2021_EPI_ISL_3663591_2021-07-10	India	2021-04-05;2021-07-10	no
Mozambique/CERI-KRISP-K025853/2021_EPI_ISL_5425648_2021-05-07	undetermined/UK	Before 2021-05-07	no
Mozambique/CERI-KRISP-K025858/2021_EPI_ISL_5425649_2021-07-07	UK	2021-05-01;2021-07-07	no
Mozambique/CERI-KRISP-K025861/2021_EPI_ISL_5425680_2021-08-07	undetermined	Before 2021-08-07	no
Mozambique/CERI-KRISP-K025865/2021_EPI_ISL_5425792_2021-07-10	undetermined	Before 2021-057-10	no
Mozambique/CERI-KRISP-K025875/2021_EPI_ISL_5425701_2021-07-08	undetermined	Before 2021-07-08	no
Mozambique/CERI-KRISP-K025889/2021_EPI_ISL_5425756_2021-07-09	UK	2021-05-24;2021-07-09	no
Mozambique/CERI-KRISP-K025898/2021_EPI_ISL_5425779_2021-07-07	UK	2021-05-01;2021-07-07	no
Mozambique/CERI-KRISP-K025905/2021_EPI_ISL_5425714_2021-07-06	undetermined/South Africa	Before 2021-07-06	no
Mozambique/CERI-KRISP-K025909/2021_EPI_ISL_5425658_2021-07-02	undetermined	Before 2021-07-02	no

Mozambique/CERI-KRISP-K025912/2021_EPI_ISL_5425639_2021-06-25	India	2021-04-30;2021-06-28	no
Mozambique/CERI-KRISP-K025916/2021_EPI_ISL_5425650_2021-06-28	India	2021-04-30;2021-06-28	no
Mozambique/CERI-KRISP-K025918/2021_EPI_ISL_5425789_2021-06-28	undetermined	Before 2021-06-28	no
Mozambique/CERI-KRISP-K025920/2021_EPI_ISL_5425710_2021-07-02	Israel	2021-06-28;2021-07-02	no
Mozambique/CERI-KRISP-K025922/2021_EPI_ISL_5425723_2021-06-30	undetermined	Before 2021-06-30	no
Mozambique/CERI-KRISP-K025923/2021_EPI_ISL_5425760_2021-07-05	undetermined/South Africa	Before 2021-07/05	no
Mozambique/CERI-KRISP-K025926/2021_EPI_ISL_5425641_2021-07-05	undetermined/UK	Before 2021-07-05	no
Mozambique/CERI-KRISP-K025927/2021_EPI_ISL_5425721_2021-06-30	undetermined/UK	Before 2021-06-30	no
Mozambique/CERI-KRISP-K025933/2021_EPI_ISL_5425693_2021-06-25	undetermined	Before 2021-06-25	no
Mozambique/CERI-KRISP-K026080/2021_EPI_ISL_5942708_2021-07-05	undetermined	Before 2021-07-05	no
Mozambique/CERI-KRISP-K026085/2021_EPI_ISL_5942696_2021-07-05	India	2021-04-30;2021-07-05	no
Mozambique/CERI-KRISP-K026090/2021_EPI_ISL_5942674_2021-07-06	undetermined	Before 2021-07-06	no
Mozambique/CERI-KRISP-K026109/2021_EPI_ISL_5942786_2021-07-10	South Africa	2021-07-07;2021-07-10	no
Mozambique/CERI-KRISP-K026112/2021_EPI_ISL_5942710_2021-07-19	undetermined	Before 2021-07-19	no
Mozambique/CERI-KRISP-K026131/2021_EPI_ISL_5942698_2021-07-16	undetermined/South Africa	Before 2021-07-16	no
Mozambique/CERI-KRISP-K026137/2021_EPI_ISL_5942773_2021-07-19	India	2021-04-30;2021-07-19	no
Mozambique/CERI-KRISP-K026140/2021_EPI_ISL_5942707_2021-07-15	undetermined/South Africa	Before 2021-07-14	no
Mozambique/CERI-KRISP-K026143/2021_EPI_ISL_5942815_2021-07-15	undetermined	Before 2021-07-15	no
Mozambique/CERI-KRISP-K026145/2021_EPI_ISL_5942741_2021-07-15	Brazil	2021-07-14;2021-07-15	no
Mozambique/CERI-KRISP-K026148/2021_EPI_ISL_5942671_2021-07-19	Brazil	2021-07-14;2021-07-19	no
Mozambique/CERI-KRISP-K026166/2021_EPI_ISL_5942752_2021-07-26	Zambia/Malawi	2021-05-29;2021-07-26	no
Mozambique/CERI-KRISP-K026167/2021_EPI_ISL_5942825_2021-07-31	undetermined	Before 2021-07-31	no
Mozambique/CERI-KRISP-K027119/2021_EPI_ISL_6511754_2021-07-27	UK	2021-07-01;2021-07-27	no
Mozambique/CERI-KRISP-K027123/2021_EPI_ISL_6511755_2021-07-27	India	2021-06-01;2021-07-27	no
Mozambique/CERI-KRISP-K027125/2021_EPI_ISL_6511757_2021-07-26	undetermined	Before 2021-07-26	no
Mozambique/CERI-KRISP-K027128/2021_EPI_ISL_6511760_2021-07-26	India	2021-04-05;2021-07-26	no
Mozambique/CERI-KRISP-K027129/2021_EPI_ISL_6511761_2021-07-26	India	2021-04-05;2021-07-26	no
Mozambique/CERI-KRISP-K027130/2021_EPI_ISL_6511762_2021-07-26	undetermined/UK	Before 2021-07-26	no
Mozambique/CERI-KRISP-K027132/2021_EPI_ISL_6511764_2021-07-28	India	2021-04-05;2021-07-28	no
Mozambique/CERI-KRISP-K027133/2021_EPI_ISL_6511765_2021-07-27	UK	2021-07-01;2021-07-27	no

Mozambique/CERI-KRISP-K027134/2021_EPI_ISL_6511766_2021-07-26	South Africa	2021-06-29;2021-07-26	no
Mozambique/CERI-KRISP-K027135/2021_EPI_ISL_8799642_2021-07-26	undetermined	Before 2021-07-26	no
Mozambique/CERI-KRISP-K027136/2021_EPI_ISL_8799643_2021-07-27	India	2021-04-19;2021-07-27	no
Mozambique/CERI-KRISP-K027137/2021_EPI_ISL_8799644_2021-07-27	undetermined	Before 2021-07-27	no
Mozambique/CERI-KRISP-K027138/2021_EPI_ISL_8799645_2021-07-27	Uganda	2021-06-15;2021-07-27	no
Mozambique/CERI-KRISP-K027139/2021_EPI_ISL_8799646_2021-07-27	India	2021-04-05;2021-07-27	no
Mozambique/CERI-KRISP-K027141/2021_EPI_ISL_8799648_2021-07-27	India	2021-04-05;2021-07-27	no
Mozambique/CERI-KRISP-K027143/2021_EPI_ISL_8799650_2021-07-12	India	2021-06-01;2021-07-12	no
Mozambique/CERI-KRISP-K027151/2021_EPI_ISL_8799651_2021-07-08	South Africa	2021-06-17;2021-07-08	no
Mozambique/CERI-KRISP-K027158/2021_EPI_ISL_8799653_2021-10-07	India	Before 2021-10-07	no
Mozambique/CERI-KRISP-K027172/2021_EPI_ISL_8799661_2021-07-13	UK	2021-05-01;2021-07-13	no
Mozambique/CERI-KRISP-K027178/2021_EPI_ISL_8799667_2021-07-21	undetermined/UK	Before 2021-07-21	no
Mozambique/CERI-KRISP-K027187/2021_EPI_ISL_8799675_2021-07-13	undetermined	Before 2021-07-13	no
Mozambique/CERI-KRISP-K027200/2021_EPI_ISL_8799679_2021-07-14	India	2021-05-07;2021-07-14	no
Mozambique/CERI-KRISP-K027528/2021_EPI_ISL_9084388_2021-08-04	France	2021-07-05;2021-08-04	no
Mozambique/CERI-KRISP-K027537/2021_EPI_ISL_9084391_2021-08-11	undetermined	Before 2021-08-11	no
Mozambique/CERI-KRISP-K027553/2021_EPI_ISL_9084400_2021-08-11	undetermined	Before 2021-08-11	no
Mozambique/CERI-KRISP-K027945/2021_EPI_ISL_7417350_2021-09-02	undetermined/UK	Before 2021-09-02	no
Mozambique/CERI-KRISP-K027969/2021_EPI_ISL_7417295_2021-09-25	South Africa	2021-08-13;2021-09-25	no
Mozambique/CERI-KRISP-K027970/2021_EPI_ISL_7417303_2021-09-24	Portugal	2021-08-09;2021-09-04	no
Mozambique/CERI-KRISP-K027975/2021_EPI_ISL_7417221_2021-09-01	undetermined/South Africa	Before 2021-09-01	no
Mozambique/CERI-KRISP-K027983/2021_EPI_ISL_7417061_2021-09-03	South Africa	2021-08-19;2021-09-03	no
Mozambique/CERI-KRISP-K027988/2021_EPI_ISL_7417314_2021-09-13	undetermined	Before 2021-09-13	no
Mozambique/CERI-KRISP-K027990/2021_EPI_ISL_7417073_2021-09-13	India	2021-04-24;2021-09-13	no
Mozambique/CERI-KRISP-K030819/2021_EPI_ISL_6795181_2021-08-31	India	2021-06-16;2021-08-31	no
Mozambique/CERI-KRISP-K030826/2021_EPI_ISL_6795184_2021-09-01	Malawi	2021-09-01;2021-09-01	no
Mozambique/COV-2090-A1713/2021_EPI_ISL_5822687_2021-06-21	undetermined/UK	Before 2021-06-21	no
Mozambique/COV-2092-A1715/2021_EPI_ISL_5822575_2021-06-21	undetermined/UK	Before 2021-06-21	no
Mozambique/COV-2095-A1718/2021_EPI_ISL_5822578_2021-06-21	undetermined/UK	Before 2021-06-21	no
Mozambique/COV-2096-A1719/2021_EPI_ISL_5822579_2021-06-21	India	2021-04-30;2021-06-28	no

Mozambique/COV-2097-A1720/2021_EPI_ISL_5822580_2021-06-21	UK	2021-05-01;2021-06-21	no
Mozambique/COV-2100-A1723/2021_EPI_ISL_5822582_2021-06-18	UK	2021-05-01;2021-06-18	no
Mozambique/COV-2146-A1793/2021_EPI_ISL_5822588_2021-06-21	South Africa	2021-05-26;2021-06-21	no
Mozambique/COV-2159-A1806/2021_EPI_ISL_5822589_2021-06-17	undetermined	Before 2021-06-17	no
Mozambique/COV-2167-A1814/2021_EPI_ISL_5822592_2021-06-21	UK	2021-05-01;2021-06-21	no
Mozambique/COV-2169-A1816/2021_EPI_ISL_5822593_2021-06-16	India	2021-04-30;2021-06-28	no
Mozambique/COV-2176-A1823/2021_EPI_ISL_5822690_2021-06-17	undetermined/UK	Before 2021-06-17	no
Mozambique/COV-2181-A1828/2021_EPI_ISL_5822598_2021-06-17	undetermined/UK	Before 2021-06-17	no
Mozambique/COV-2182-A1829/2021_EPI_ISL_5822599_2021-06-14	undetermined	Before 2021-06-14	no
Mozambique/COV-2187-A1834/2021_EPI_ISL_5822688_2021-06-22	undetermined/UK	Before 2021-06-22	no
Mozambique/COV-2190-A1837/2021_EPI_ISL_5822600_2021-06-18	undetermined/UK	Before 2021-06-18	no
Mozambique/COV-2191-A1838/2021_EPI_ISL_5822601_2021-06-16	UK	2021-06-01;2021-06-06	no
Mozambique/COV-2195-A1842/2021_EPI_ISL_5822693_2021-06-18	India	2021-04-30;2021-06-28	no
Mozambique/COV-2212-A1859/2021_EPI_ISL_5822611_2021-06-18	undetermined/UK	Before 2021-06-18	no
Mozambique/COV-2216-A1863/2021_EPI_ISL_5822613_2021-06-21	undetermined/UK	Before 2021-06-21	no
Mozambique/COV-2218-A1865/2021_EPI_ISL_5822615_2021-06-21	undetermined/UK	Before 2021-06-21	no
Mozambique/COV-2220-A1867/2021_EPI_ISL_5822675_2021-06-21	undetermined/UK	Before 2021-06-21	no
Mozambique/COV-2230-A1878/2021_EPI_ISL_5822623_2021-06-21	undetermined/UK	Before 2021-06-21	no
Mozambique/COV-2237-A1885/2021_EPI_ISL_5822630_2021-06-21	undetermined/UK	Before 2021-06-21	no
Mozambique/COV-2244-A1892/2021_EPI_ISL_5822634_2021-06-21	UK	2021-05-01;2021-06-21	no
Mozambique/COV-2254-A1902/2021_EPI_ISL_5822636_2021-06-17	South Africa	2021-06-15;2021-06-17	no
Mozambique/COV-2255-A1903/2021_EPI_ISL_5822637_2021-06-17	undetermined/South Africa	Before 2021-06-17	no
Mozambique/COV-2260-A1908/2021_EPI_ISL_5822641_2021-06-24	undetermined	Before 2021-06-24	no
Mozambique/COV-2310-A1958/2021_EPI_ISL_5822655_2021-06-22	UK	2021-05-01;2021-06-22	no
**total_Mozambican_seqs**	**171**		
**new_seqs**	**42**		

Delta unclassified sequences	
**Sample_name**	**new_seq**
hCoV-19/Mozambique/CERI-KRISP-K021116/2021_2021-06-29_2021-08-25	no
hCoV-19/Mozambique/CERI-KRISP-K021123/2021_2021-07-02_2021-08-25	no
hCoV-19/Mozambique/CERI-KRISP-K021142/2021_2021-07-05_2021-08-25	no
hCoV-19/Mozambique/CERI-KRISP-K021180/2021_2021-07-13_2021-08-25	no
hCoV-19/Mozambique/CERI-KRISP-K021183/2021_2021-07-13_2021-08-25	no
hCoV-19/Mozambique/COV-2157-A1804/2021_2021-05-05_2021-11-01	no
hCoV-19/Mozambique/COV-2314-A1962/2021_2021-06-16_2021-11-01	no
Mozambique_COV035932	**yes**
Mozambique_COV035947	**yes**
Mozambique_COV035949	**yes**
Mozambique_COV035975	**yes**
Mozambique_COV035977	**yes**
Mozambique_COV035985	**yes**
Mozambique_COV035987	**yes**
Mozambique_COV035994	**yes**
Mozambique_COV035998	**yes**
Mozambique_COV036000	**yes**
Mozambique_COV036003	**yes**
Mozambique_COV036012	**yes**
Mozambique_COV036019	**yes**
Mozambique_COV036026	**yes**
Mozambique_COV036033	**yes**
Mozambique_COV036036	**yes**
Mozambique_COV036047	**yes**
Mozambique_COV036064	**yes**
Mozambique_COV036071	**yes**
Mozambique_COV036079	**yes**

Mozambique/CERI-KRISP-K021120/2021_EPI_ISL_3663550_2021-07-01	no
Mozambique/CERI-KRISP-K021207/2021_EPI_ISL_3663603_2021-07-13	no
Mozambique/CERI-KRISP-K025914/2021_EPI_ISL_5425640_2021-06-28	no
Mozambique/CERI-KRISP-K026146/2021_EPI_ISL_5942719_2021-07-15	no
Mozambique/CERI-KRISP-K027142/2021_EPI_ISL_8799649_2021-07-27	no
Mozambique/COV-2086-A1709/2021_EPI_ISL_5822571_2021-06-17	no
Mozambique/COV-2088-A1711/2021_EPI_ISL_5822572_2021-06-16	no
Mozambique/COV-2091-A1714/2021_EPI_ISL_5822574_2021-06-21	no
Mozambique/COV-2094-A1717/2021_EPI_ISL_5822577_2021-06-17	no
Mozambique/COV-2101-A1724/2021_EPI_ISL_5822676_2021-06-21	no
Mozambique/COV-2133-A1780/2021_EPI_ISL_5822583_2021-06-17	no
Mozambique/COV-2136-A1783/2021_EPI_ISL_5822586_2021-06-17	no
Mozambique/COV-2166-A1813/2021_EPI_ISL_5822591_2021-06-21	no
Mozambique/COV-2179-A1826/2021_EPI_ISL_5822596_2021-06-21	no
Mozambique/COV-2207-A1854/2021_EPI_ISL_5822608_2021-06-18	no
Mozambique/COV-2214-A1861/2021_EPI_ISL_5822612_2021-06-21	no
Mozambique/COV-2258-A1906/2021_EPI_ISL_5822698_2021-06-24	no
Mozambique/COV-2259-A1907/2021_EPI_ISL_5822640_2021-06-24	no
Mozambique/COV-2263-A1911/2021_EPI_ISL_5822644_2021-06-06	no
Mozambique/COV-2278-A1926/2021_EPI_ISL_5822647_2021-06-25	no
Mozambique/COV-2281-A1929/2021_EPI_ISL_5822649_2021-06-21	no
Mozambique/COV-2292-A1940/2021_EPI_ISL_5822653_2021-05-31	no
Mozambique/COV-2356-A2005/2021_EPI_ISL_5822650_2021-05-27	no
Mozambique_COV035970	**yes**
Mozambique_COV036021	**yes**
Mozambique_COV036034	**yes**
Mozambique/CERI-KRISP-K021154/2021_EPI_ISL_3663564_2021-07-07	no
Mozambique/CERI-KRISP-K021156/2021_EPI_ISL_3663566_2021-07-12	no
Mozambique/CERI-KRISP-K021197/2021_EPI_ISL_3663594_2021-07-10	no
Mozambique/CERI-KRISP-K021201/2021_EPI_ISL_3663598_2021-07-07	no

Mozambique/CERI-KRISP-K026165/2021_EPI_ISL_5942667_2021-07-30	no
Mozambique/COV-2178-A1825/2021_EPI_ISL_5822595_2021-06-17	no
Mozambique/COV-2227-A1874/2021_EPI_ISL_5822620_2021-06-21	no
Mozambique/COV-2264-A1912/2021_EPI_ISL_5822659_2021-06-05	no
Mozambique/COV-2265-A1913/2021_EPI_ISL_5822660_2021-06-18	no
Mozambique/COV-2266-A1914/2021_EPI_ISL_5822664_2021-06-17	no
Mozambique/COV-2357-A2006/2021_EPI_ISL_5822651_2021-05-29	no
**total_Mozambican_seqs**	**64**
**new_seqs**	**23**

Benchmarking customized approach vs RASP		
**Transmission group**	**Sample of origin of the introduction**	**Distance to sample of origin (# SNPs)**
G02	SouthAfrica/VIDA-KRISP-V001012/2020_EPI_ISL_940882_2020-12-21	2
G03	SouthAfrica/NICD-N2063/2021_EPI_ISL_2662470_2021-01-26	6
G04	SouthAfrica/VIDA-KRISP-K008043/2021_EPI_ISL_940852_2021-01-07	4
G05	England/QEUH-CAF17D/2020_OD913545.1_2020-12-15	3
G06	SouthAfrica/UFS-VIRO-NGS-261/2021_EPI_ISL_1818892_2021-01-11	4
G07	SouthAfrica/VIDA-KRISP-K012605/2021_EPI_ISL_2494367_2020-12-22	4
G10	Malawi/CERI-KRISPK015750/2021_EPI_ISL_2609570_2021-01-25, SouthAfrica/N[illegible]	3
G13	SouthAfrica/PFE_00003/2021_EPI_ISL_3800565_2021-01-11	5
G14	England/NORW-F24BA/2020_OU815065.1_2020-12-29 / SouthAfrica/KRISP-K007[illegible]	2
G15	hCoV-19/South_Africa/NICD-N4543/2021_2021-02-16_2021-05-13	1
G16	SouthAfrica/KRISP-K007823/2021_EPI_ISL_860592_2021-01-02	2
G17	Denmark/DCGC-28407/2021_EPI_ISL_844979_2021-01-04, SouthAfrica/PFE_000[illegible]	4
G20	SouthAfrica/KRISP-K010009/2020_EPI_ISL_1250468_2020-12-23	2
G22	SouthAfrica/NICD-N19798/2020_EPI_ISL_6906491_2020-12-28	3
G26	SouthAfrica/KRISP-K010026/2020_EPI_ISL_1250494_2020-12-21	1
G31	SouthAfrica/N4543/2021_EPI_ISL_2086954_2021-02-16	3
G32	SouthAfrica/VIDA-KRISP-K010570/2020_EPI_ISL_2360368_2020-12-09	3
G33	Zimbabwe/CERI-KRISP-K011611/2020_EPI_ISL_2492651_2020-12-17	8
G36	Malawi/KRISP-K010214/2021_EPI_ISL_1407164_2021-01-12	1
G38	SouthAfrica/N2311/2021_EPI_ISL_2582749_2020-12-28	4
G42	SouthAfrica/N00899/2020_EPI_ISL_1239287_2020-11-17	1

**Origin proportion according to RASP (%)**	
More than 95% South Africa	
More than 95% South Africa	
More than 95% South Africa	
68.13 South Africa, 15.40 United Kingdom, 12.37 Eswatini, 1.93 South Africa / United Kingdom, 1.55 South Africa / Eswatini, 0.62 United Kingdom / Eswat	
More than 95% South Africa	
More than 95% South Africa	
41.81 Malawi, 40.55 South Africa, 14.21 South Africa / Malawi, 1.02 Mozambique, 3.43 from different mixed origins	
More than 95% South Africa	
92.29 South Africa, 4.34 United Kingdom/South Africa, 2.73 Mozambique, 0.64 United Kingdom	
89.22 South Africa, 3.57 South Africa / Mozambique, 2.38 South Africa / Eswatini, 1.54 Mozambique, 1.03 Eswatini, 2.26 from different mixed origins	
More than 95% South Africa	
95.85 South Africa, 1.66 South Africa / Mozambique, 0.82 South Africa / Denmark, 0.57 Mozambique, 0.28 Denmark, 0.72 from different mixed origins	
More than 95% South Africa	
More than 95% South Africa	
More than 95% South Africa	
More than 95% South Africa	
More than 95% South Africa	
91.74 South Africa, 6.27 South Africa / Zimbabwe, 1.16 Zimbabwe, 0.83 from different mixed origins	
99.63 South Africa, 0.16 South Africa/Malawi, 0.21 from different mixed origins	
More than 95% South Africa	
More than 95% South Africa	

ini

Supplementary File

Extended methods

Study design

This genomic epidemiology study covered Mozambique, a South African country with 31,616,078 inhabitants and a population density of 40 inhabitants per Km^2 in 2022.[1] The SARS-CoV-2 samples used in this study were obtained from the MozCOVID study and the National Surveillance System, which were prospective and retrospective studies, respectively (appendix, pp 1-35). In the MozCOVID study, all individuals attending the Manhiça District Hospital were pre-screened in the outpatient consultation or urgencies, by a medical doctor, and if they fulfilled the criteria for testing they were sent to the SARS-CoV-2 testing unit. In the unit, if the patient presented respiratory symptoms and resided in the Manhiça District, a medical technician of the study was notified and asked the patient to be part of the study and a questionnaire was filled (appendix, pp 36-39). All individuals not meeting the inclusion criteria for the study were tested in the normal circuit for the national surveillance. In addition, only subjects with their first documented SARS-CoV-2 infection were recruited. The consents for the study research were then administered by a medical technician of the study. Additional cases also living in Manhiça District were recruited from the National Surveillance Program. Medical technicians and nurses used tablets to collect demographic data which, after revision by a medical doctor to check inconsistencies and errors, were introduced in RedCap.

A laboratory requisition was filled in a paper form and then introduced in a sample management software (ServoLab), where all laboratory results were introduced, including CT values. Questionnaires from the National Surveillance System were not available for inclusion in this study.

qPCR for SARS-CoV-2 detection

Nasopharyngeal swabs were collected from all patients to detect SARS-CoV-2 by Real-Time Polymerase Chain Reaction (qPCR), using either Abbot RealTime SARS-CoV-2 with dual target assay for the RdRp and N genes (Abbot Molecular Inc., Illinois, USA) or TaqMan™ 2019 nCoV Assay Kit v1 with orf1ab, spike (S) gene, nucleocapsid (N) gene, RNase P) target assay (Thermo Fisher Scientific, Massachusetts, USA) according to the manufacturer's instructions. From positive samples (N=2,259), only those with cycle threshold less than 27 were selected for sequencing (N=384).

NGS sequencing

RNA was retro-transcribed into cDNA, then the SARS-CoV-2 complete genome amplification was performed in two multiplex PCR, accordingly to openly available protocol developed by the ARTIC network using the V3 multiplex primers scheme.[2] Two resulting amplicon pools were combined and used for library preparation. Genomic libraries were constructed with the Nextera DNA Flex Sample Preparation kit (Illumina Inc., San Diego, USA) according to the manufacturer's protocol with five cycles for indexing PCR. Whole genome sequencing was carried out in the Illumina MiSeq platform (Illumina Inc., San Diego, USA) using 2×200 cycles paired-end run. Library preparation and sequencing was performed at Instituto de Biomedicina de Valencia (IBV, CSIC). Sequences with low genome coverage (<0.90, N=99) and replicates from the same patient were excluded (N=5). We obtained 280 good quality new SARS-CoV-2 sequences after the inclusion criteria. In addition, we included 652 Beta and Delta public sequences from Mozambique (Figure 2). Inclusion criteria and sample source are detailed in Figure 3A.

Genomic analysis

Sequence data was analysed following the SeqCOVID-Spain consortium pipeline already published,[3] and available in the lab repository.[4] Briefly: 1) human reads were removed with Kraken ; 2) fastq files were filtered with fastp v 0.20.1 5 (arguments: --cut tail, --cut-window-size, --cut-mean-quality , -max_len1 ,-max_len2); 3) bwa and IVAR v1.2 were used for mapping, variant calling and consensus generation (variant calling cut-offs: minimum quality for SNP calling=20, minimum frequency to call a SNP=0·05, minimum depth for calling a SNP=20, consensus construction cut-offs: minimum quality

for consensus calling=20, minimum frequency to consider fixed a SNP=0·8, minimum position depth=30 (ambiguous base otherwise)). The Pangolin tool (version v.3.1.16) was run on the obtained consensus sequences for lineage assessment (Supplementary Figure 1) using the nomenclature of PANGO-designation database (v.1.2.93, release 2021-11-09).[5,6] Reads were mapped against the SARS-CoV-2 reference genome Wuhan-Hu-1 (GenBank: MN908947.3). Graphics were performed with *ggplot2* package from R. Scripts were coded in R and python (libraries: doParallel, foreach, treeio, stringr, castor, writexl).

Phylogenetic placement of sequences using UShER
UShER is a phylogenetic tool that allows rapid sample placement in a pre-existing phylogeny using a maximum parsimony score.[7] We downloaded a global pre-built or existing phylogenetic tree (eTree) generated by The University of California Santa Cruz Genomic Institute stored at their FTP server, on February 9, 2022.[8] The phylogeny included 7,657,161 global SARS-CoV-2 sequences, including all variants described until February 9, 2022. Since we aimed to have all sequenced samples in the phylogeny, we searched for sequences deposited in GISAID belonging to those lineages present in our dataset that were not included in the eTree.[9] The lineages of interest were B.1.351, or Beta, and the following Delta lineages: AY.10, AT.19, AY.29, AY.37, AY.6, B.1.617.2, AY.111, AY.112, AY.116, AY.121, AY.122, AY.25.3, AY.38, AY.43, AY.45, AY.65, AY.75, AY.87, AY.88, and AY.91. We downloaded a total of 1,971 and 85,560 missing Beta and Delta sequences, respectively. We then performed the phylogenetic placement of these sequences, along with the new ones obtained in this study, by applying the methodology described by Turakhia et al.,[10] also available at the UShER wiki.[11] Briefly, separately for Beta and Delta lineages' samples we aligned all consensus sequences against the SARS-CoV-2 Wuhan-Hu-1 reference genome (GenBank: MN908947.3) using a script developed by Robert Lanfear.[12] Then, we added the reference genome to the alignment and obtained the variants in VCF format using faToVcf with the argument "-maskSites", so we can mask those positions suspected to be problematic for phylogenetic inference.[13] Finally we used both the phylogeny and the VCF as inputs for UShER to perform the placement. Once we had both phylogenies, we used MatUtils to convert them into a format suitable for their visualisation on Taxonium,[14,15] so we could choose the clades enriched in Beta and Delta. We cut these clades off their corresponding tree by using *ape* package from R. Eventually, we obtained a Beta subtree with 45,831 samples (373 from Mozambique, 112 of them were generated in this study), and a Delta subtree with 4,403,383 samples (559 from Mozambique, 143 of them were generated in this study).

Identification of transmission groups and unique sequences
We used the classification proposed by López et al.[3] where transmission groups were defined as monophyletic clades enriched in Mozambican sequences (i.e containing minimum 60% of Mozambican sequences), and a basal/ancestral sequence also from Mozambique. They likely represent strains in transmission within Mozambique and, in some cases, later exported to other countries. Unique sequences were defined as sequences that do not share an immediate common ancestor with any other Mozambican sequence, but are genetically differentiated from the closest sequences by at least one SNP, and no more than 10. Any sequence not meeting those criteria was defined as unclassified (Supplementary Figure 2). We developed a custom code that scans Beta and Delta subtrees and classifies all the sequences in one of these categories.[16] Ambiguous sequences were manually inspected.

Phylogenetic reconstruction of Beta (lineage B.1.351)
We evaluated the accuracy of our approach by comparing the results obtained by the phylogenetic placement versus using a phylogeny reconstructed with IQ-TREE.[17] All sequences in GISAID classified as B.1.351 as of December 14, 2021 (n=34,051) were aligned against the reference genome (MN908947.3) using Robert Lanfear's global_alignment_profile.sh script as aforementioned.[12] As described in the literature, some positions within the SARS-CoV-2 genome are suspected to be problematic for phylogenetic inference. To avoid spurious phylogenetic relations,

these positions were masked using mask_alignment.sh script, also designed by Robert Lanfear. Later, we used the multisequence alignment file to run IQ-TREE as detailed in the MozCOVID_commands file in Git repository). We reconstructed a maximum-likelihood (ML) phylogeny, rooted with the SARS-CoV-2 sequence obtained in Wuhan on 24/12/2019 (GISAID ID: EPI_ISL_402123).

Introduction identification and origin

To investigate the potential origin of the introductions, we sought the closest non-Mozambique sequence in the phylogeny for each transmission group and unique sequence, separated by minimum 1 SNP and no more than 10 SNPs; and with a diagnosis date happening within 3 months prior to the first case of the transmission group or unique sequence. If there was more than one possible country of origin, but all possibilities were African countries, we defined the origin as African. Introductions were considered as undetermined if the closest sequence had been collected earlier than 3 months, or if many origins at the same minimal distance were equally possible.

Benchmark between IQ-TREE and UShER

To further assess the robustness between both methods, we compared the topologies of the Beta subtree obtained with UShER against the Beta phylogeny reconstructed with IQ-TREE by performing the Shimodaira-Hasegawa test (SH test) with IQ-TREE (as detailed in the MozCOVID_commands file in Git repository),[18] which tests whether the Maximum-Likelihood score (ML score) is significantly different between a set of trees, given a reference alignment.

Origin of introduction benchmark

With the aim of further evaluating the accuracy of our methodology in identifying introductions from huge phylogenies, we performed a comparison using RASP4 software.[19] By choosing among different statistical methods, the user is able to reconstruct the ancestral state for the nodes of a given phylogeny, which can be used to estimate the geographical origin of a transmission group only with the phylogeny and the geographical origin of the tips as input. In this case we used the Bayesian Binary Markov Chain Monte Carlo model with 500,000 cycles and 10 chains, as previously described.[20] For this comparison we used the Beta variant phylogeny. Since the high saturation of the phylogeny and the presence of numerous polytomies hinders any classical statistical analysis due to the low divergence between samples and the unbearable amount of sequences, we opted for a small-scale analysis using the identified transmission groups. For each of these groups, we tried to obtain subtrees containing all samples collected within 3 months before, up to 300 sequences, since RASP execution time increases exponentially according to the number of samples.

Extended results

Benchmarking UShER against IQ-TREE to estimate accuracy of phylogenetic placements

The first aim of our study is to compare the Mozambique sequences generated against all deposited sequences to minimise the sampling bias associated with SARS-CoV-2 phylogeographic studies. Large-scale phylogenies, in the order of millions of sequences, can be approached using recently developed phylogenetic placement methods. Our approach involved the phylogenetic placement implemented in UShER. First we placed all sequences from Beta (n=1,971) and Delta (n=85,560) variants obtained until February 9, 2022 that were not present in the backbone phylogeny provided by UShER (n=7,657,161), including the 280 generated in this study. As a total we analysed ≈46,000 Beta sequences and ≈4.4M Delta sequences. To test the accuracy of UShER placements we compared the transmission groups, unique and unclassified sequences identified for Beta maximum-likelihood phylogeny constructed with IQ-TREE as our gold standard. Overall, from the 361 common Beta sequences (Supplementary Figure 3) from Mozambique (12 out of the 373 sequences in UShER were not included in IQ-TREE, and another UShER sequence also present in IQ-TREE was discarded since it is located in a clade with a mixture of lineages), we obtain a match of 129 unique, 57

unclassified, and 133 in-transmission sequences between both methods (Supplementary Figure 3), accounting for a total of 319 (88·4%) sequences equally classified between IQ-TREE and UShER.

The script identified 39 transmission groups in the IQ-TREE phylogeny and 42 in the UShER phylogeny. Both methods share 29 groups with identical samples (74·3% of coincidence). There are 5 more groups that have a > 60% of similarity, whose discrepancies are due to the addition of one or a few samples in one method in comparison with the other. Finally, 3 IQ-TREE transmission groups have no equivalent in the UShER phylogeny. If we consider all Mozambique samples in clusters for both IQ-TREE and UShER phylogenies, we obtain that 133 of them are equally classified as part of a transmission group.

Our script identified 143 unique sequences from Mozambique in the IQ-TREE phylogeny and 139 in the UShER phylogeny. Both sets share a total of 129 samples, accounting for 90.20% of coincidence. Of the remaining 14 IQ-TREE sequences, 5 are catalogued as unclassified by UShER and 9 appear in clusters.

Regarding the unclassified samples, our script identifies 64 unclassified samples in the IQ-TREE phylogeny and 77 in the case of UShER. These two sets share 57 of the sequences, which accounts for a coincidence of 89·1%. Of the 7 remaining IQ-TREE samples, 3 appear in a group and 4 are unique introductions.

SH test p-value was 0·251 for UShER tree, as detailed in Supplementary Table 1, meaning that UShER phylogeny is not significantly different to IQ-TREE phylogeny. Furthermore, log-Likelihood of UShER tree was higher, which suggests that phylogenies reconstructed with this tool are more accurate.

Origin of introductions benchmark results

We managed to obtain subtrees for 21 of the 42 transmission groups (Appendix 3, Supp Table 8) as the other 21 were surrounded by polytomies that surpassed the limit of 300 sequences, forcing us to preselect the sequences used. The country of origin of the introductions was equally identified for 16 of the 21 groups (76·2% of match). It is important to highlight that RASP cannot take the date of the samples into account for its analysis, but only their phylogenetic relationship and the geographical origin given as input. Thus, since some of the groups come from a country with only one or few samples in the tree, RASP will not be able to consider it as a potential origin, even if it’s the most probable one due to genetic distance and time, which may explain the mismatches. All samples identified as potential origins of the introductions laid in a maximum of 10 SNPs of difference with the transmission group.

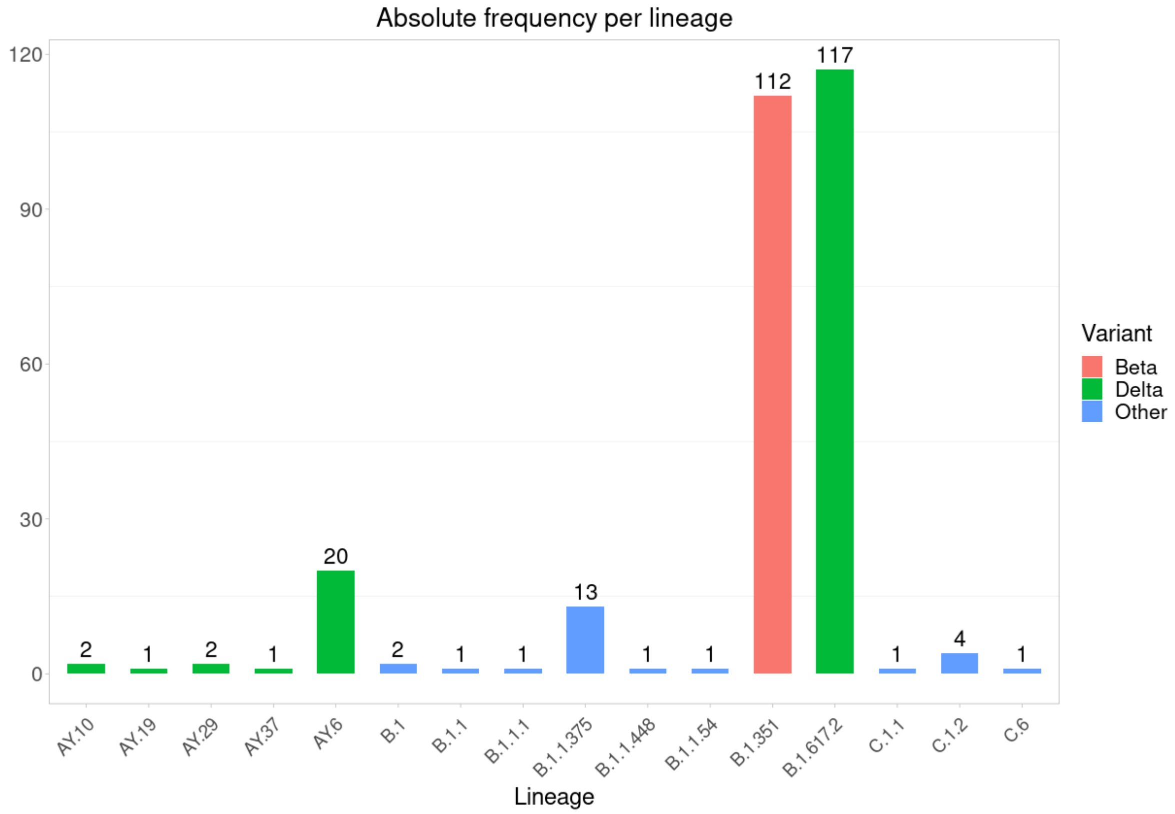

Supplementary Figure 1. SARS-CoV-2 lineage distribution in the samples sequenced from Mozambique. Y axis represents absolute frequencies.

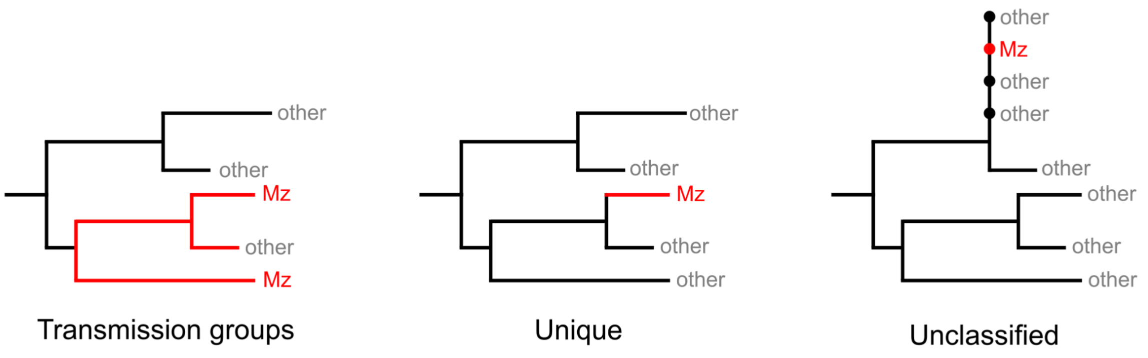

Supplementary Figure 2. Schema describing the definition used for transmission groups, unique and unclassified sequences. Mz, Mozambique

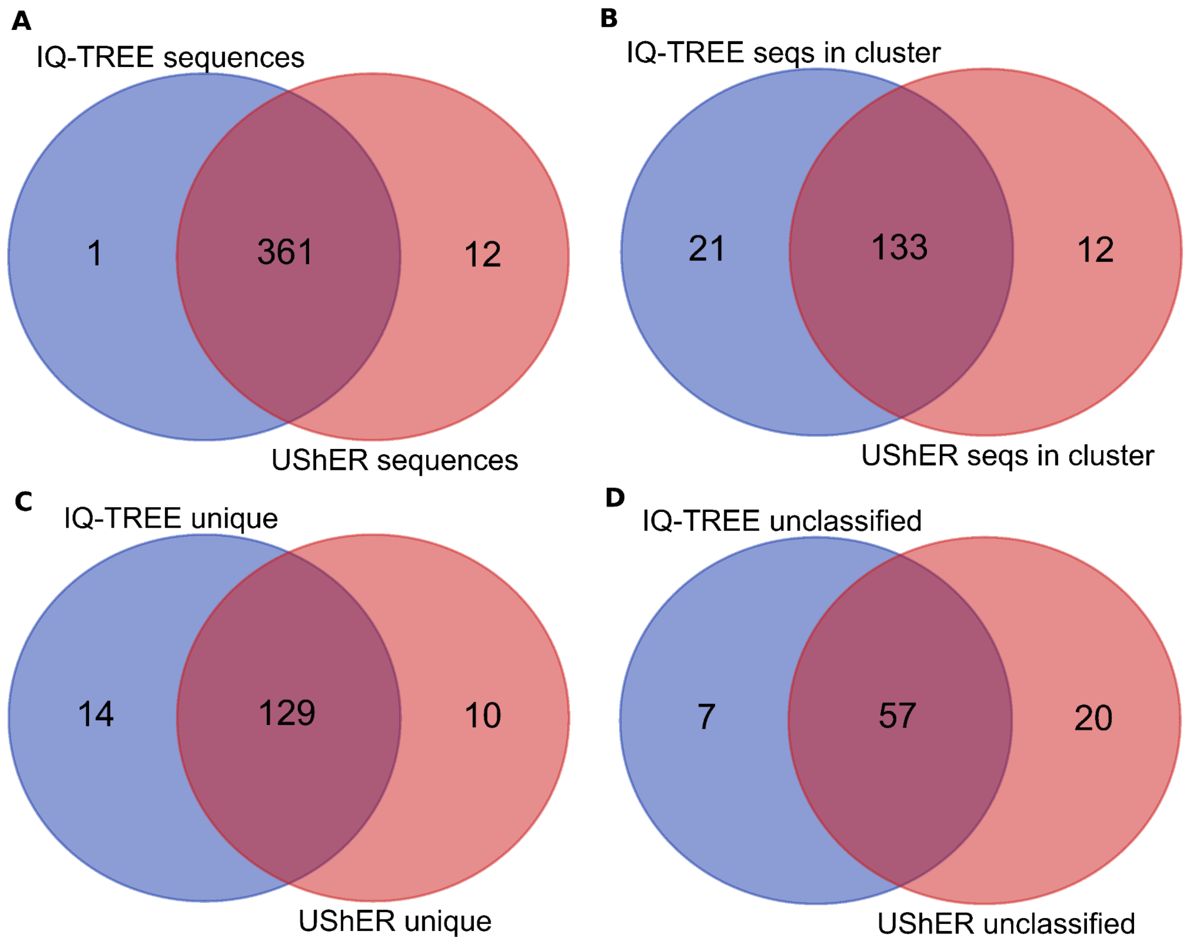

Supplementary Figure 3. IQ-TREE vs UShER results for each type of introduction

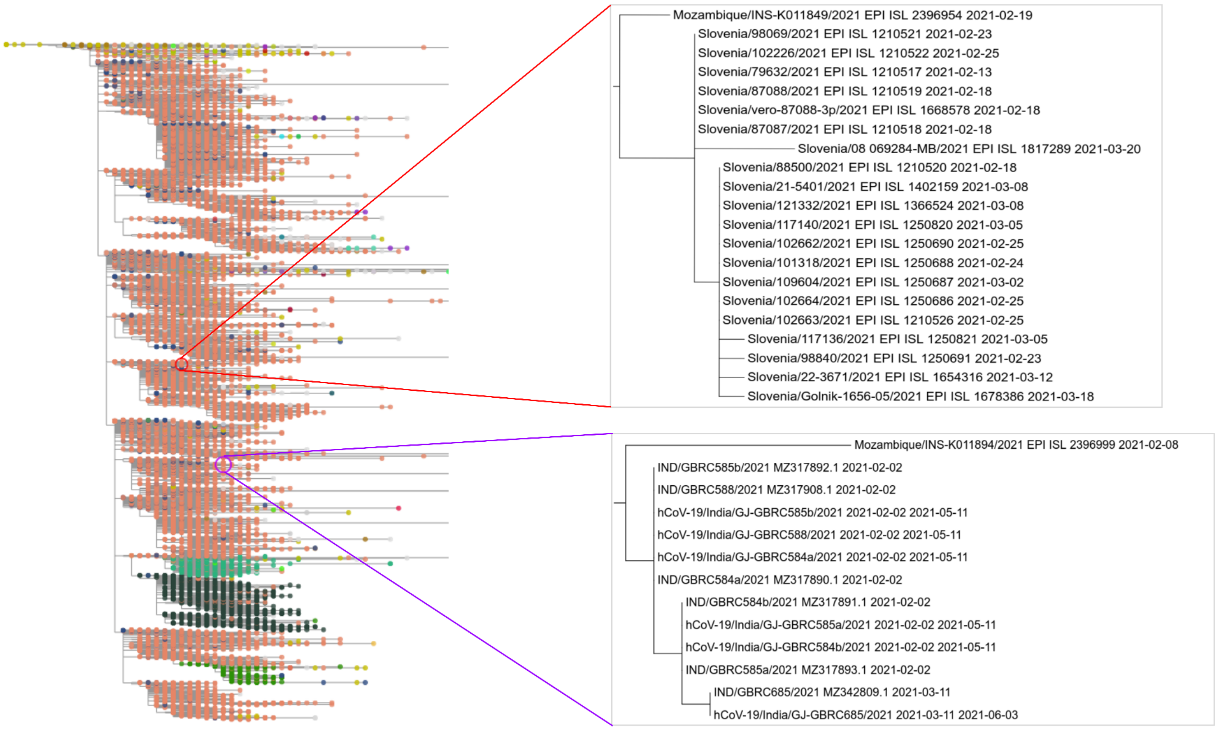

Supplementary Figure 4. Possible Beta exportations from Mozambique to Slovenia and India

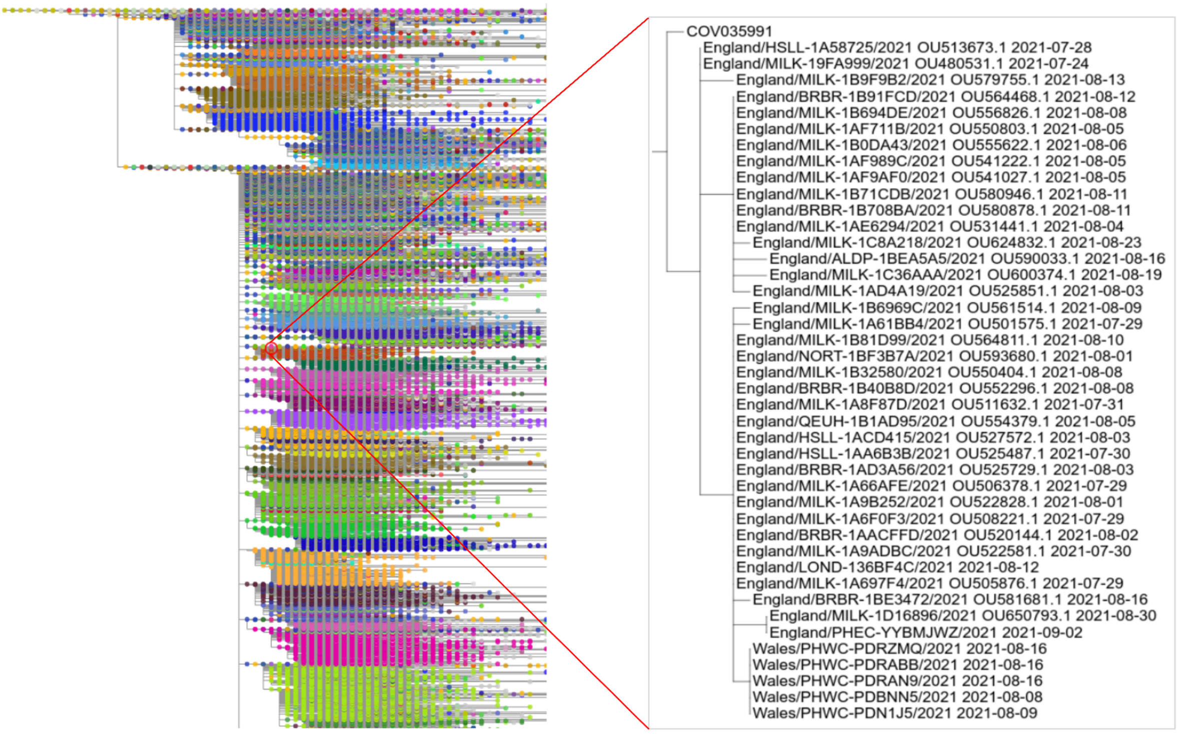

Supplementary Figure 5. Possible Delta exportation from Mozambique to the United Kingdom

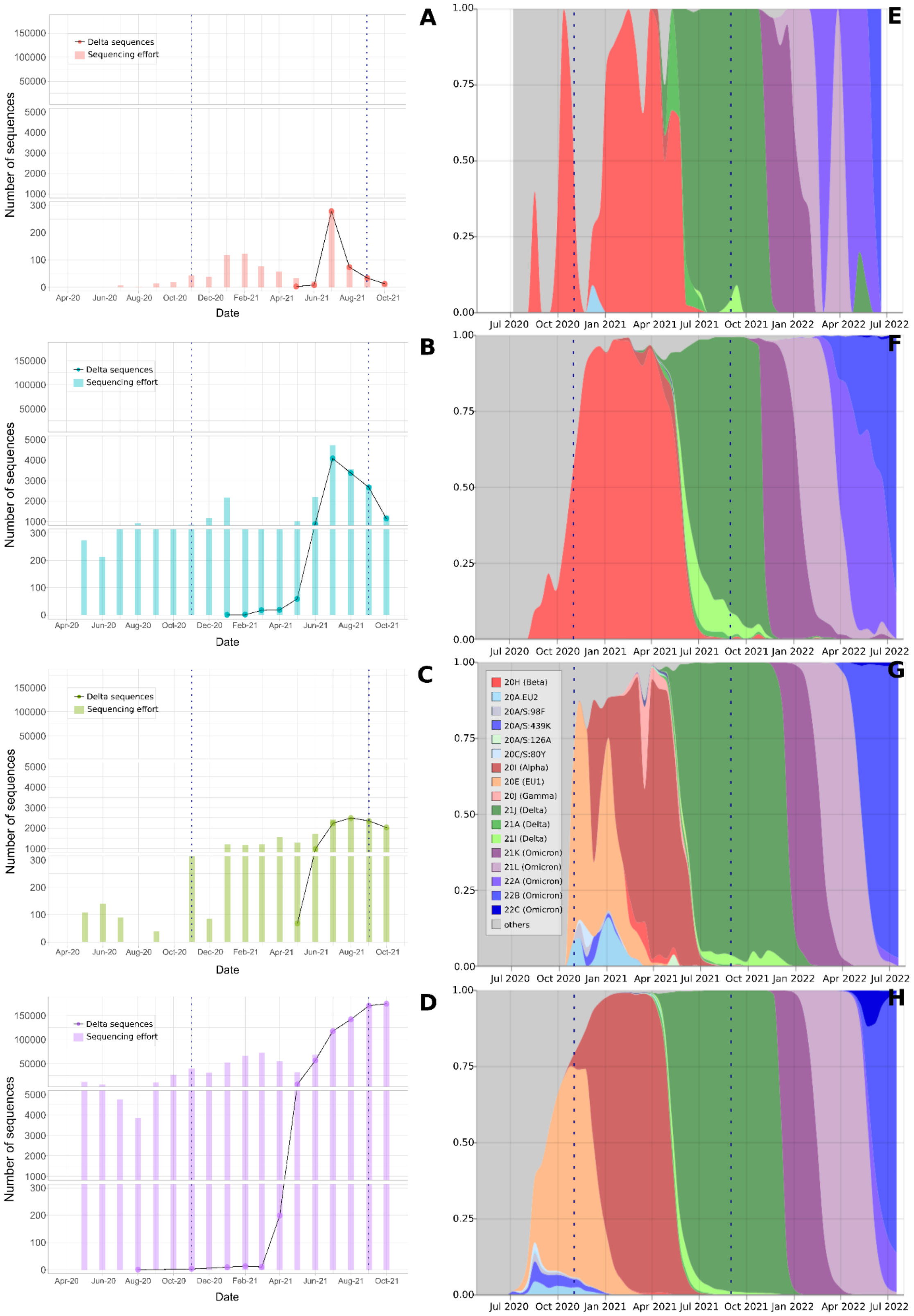

Supplementary Figure 6. Sequencing efforts and frequency of SARS-CoV-2 variants between July 2020 and August 2022. A-D**.** Sequencing effort in terms of the number of total sequences generated by different countries (bars) and the number of sequences belonging to the Delta variant (line). **E-H.** Variants' profiles as frequency of the different variants circulating in each country between July 2020 - August 2022. **A-E.** Mozambique. **B-F.** South Africa. **C-G.** Portugal. **D-H.** UK. Dotted lines indicate the study period. E-H plots have been obtained from https://covariants.org/.

Supplementary table 1. Shimodaira-Hasegawa test results for IQ-TREE and UShER phylogenies

Tree	logL	p-SH
IQ-TREE	-550,169·7985	1
UShER	-552,089·4476	0·251

References

1 INE Destaques — Instituto Nacional de Estatistica. http://www.ine.gov.mz/ (accessed January 30, 2023).

2 Quick J. nCoV-2019 sequencing protocol v3 https://www.protocols.io/view/ncov-2019-sequencing-protocol-v3-locost-bh42j8ye/v3).pdf (accessed August 25, 2020).

3 López MG, Chiner-Oms Á, García de Viedma D, *et al.* The first wave of the COVID-19 epidemic in Spain was associated with early introductions and fast spread of a dominating genetic variant. *Nat Genet* 2021; **53**: 1405–14.

4 SARS-CoV2-Mapping. GitLab. https://gitlab.com/fisabio-ngs/sars-cov2-mapping.

5 O'Toole Á, Scher E, Underwood A, *et al.* Assignment of Epidemiological Lineages in an Emerging Pandemic Using the Pangolin Tool. Virus Evolution. 2021. DOI:10.1093/ve/veab064.

6 Rambaut A, Holmes EC, O'Toole Á, *et al.* A dynamic nomenclature proposal for SARS-CoV-2 lineages to assist genomic epidemiology. *Nat Microbiol* 2020; **5**: 1403–7.

7 Turakhia Y, Thornlow B, Hinrichs AS, *et al.* Ultrafast Sample placement on Existing tRees (UShER) enables real-time phylogenetics for the SARS-CoV-2 pandemic. *Nat Genet* 2021; **53**: 809–16.

8 ftp://hgdownload.soe.ucsc.edu/apache/cgi-bin/hgPhyloPlaceData/wuhCor1/ (accessed February 9, 2022).

9 Shu Y, McCauley J. GISAID: Global initiative on sharing all influenza data - from vision to reality. *Euro Surveill* 2017; **22**. DOI:10.2807/1560-7917.ES.2017.22.13.30494.

10 Turakhia Y, Thornlow B, Hinrichs AS, *et al.* Ultrafast Sample placement on Existing tRees (UShER) enables real-time phylogenetics for the SARS-CoV-2 pandemic. *Nat Genet* 2021; **53**: 809–16.

11 UShER Wiki. https://usher-wiki.readthedocs.io/en/latest/ (accessed February 9, 2022).

12 Lanfear, R. https://github.com/roblanf/sarscov2phylo (accessed February 9, 2022).

13 Issues with SARS-CoV-2 sequencing data. https://virological.org/t/issues-with-sars-cov-2-sequencing-data/473 (accessed February 9, 2022).

14 Sanderson T. Taxonium: a web-based tool for exploring large phylogenetic trees. bioRxiv. 2022; published online June 3. DOI:10.1101/2022.06.03.494608.

15 matUtils. https://usher-wiki.readthedocs.io/en/latest/matUtils.html (accessed March 1, 2022).

16 MozCOVID. https://gitlab.com/tbgenomicsunit/mozcovid.

17 Minh BQ, Schmidt HA, Chernomor O, *et al.* IQ-TREE 2: New Models and Efficient Methods for Phylogenetic Inference in the Genomic Era. *Mol Biol Evol* 2020; **37**: 1530–4.

18 Shimodaira H, Hasegawa M. Multiple comparisons of log-likelihoods with applications to phylogenetic inference. *Mol Biol Evol* 1999; **16**: 1114–6.

19 Yu Y, Harris AJ, Blair C, He X. RASP (Reconstruct Ancestral State in Phylogenies): a tool for historical biogeography. *Mol Phylogenet Evol* 2015; **87**. DOI:10.1016/j.ympev.2015.03.008.

20 López MG, Dogba JB, Torres-Puente M, *et al.* Tuberculosis in Liberia: high multidrug-resistance burden, transmission and diversity modelled by multiple importation events. *Microbial Genomics* 2020; **6**: e000325.
